# GWAS meta-analysis of cerebrospinal fluid Alzheimer’s biomarkers reveals loci regulating lipids, brain volume and autophagy

**DOI:** 10.1038/s41467-026-71682-8

**Published:** 2026-04-21

**Authors:** Jigyasha Timsina, Chenyang Jiang, Daniel L. McCartney, Feifei Tao, Maria Carolina Dalmasso, Jenna Najar, Federica Anastasi, Olena Ohlei, Raquel Puerta Fuentes, Chenyu Yang, Joseph Bradley, Daniel Western, Muhammad Ali, Ciyang Wang, Chengran Yang, Ying Wu, Menghan Liu, John Budde, Julie Williams, Rebecca Mahoney, Atahualpa Castillo Morales, Timothy J. Hohman, Logan Dumitrescu, Ting-Chen Wang, Niccolo’ Tesi, Silke Kern, Margda Waern, Ingmar Skoog, Argonde van Harten, Yolande A. L. Pijnenburg, Wiesje M. van der Flier, Pascual Sánchez-Juan, Eloy Rodriguez-Rodriguez, Luca Kleineidam, Oliver Peters, Anja Schneider, Fahri Küçükali, Céline Bellenguez, Benjamin Grenier-Boley, Sami Heikkinen, Itziar de Rojas, Dan Rujescu, Norbert Scherbaum, Lucrezia Hausner, Emrah Düzel, Timo Grimmer, Jens Wiltfang, Rik Vandenberghe, Sebastiaan Engelborghs, Stefanie Heilmann-Heimbach, Matthias Schmid, Thomas Tegos, Nikolaos Scarmeas, Oriol Dols-Icardo, Fermin Moreno, Jordi Pérez-Tur, María J. Bullido, Raquel Sánchez-Valle, Victoria Álvarez, Pablo García-González, Pablo Mir, Luis M. Real, Gerard Piñol-Ripoll, Jose María García-Alberca, Harro Seelaar, Inez Ramakers, Janne Papma, Marc Hulsman, Christoph Laske, Stefan Teipel, Josef Priller, Robert Perneczky, Katharina Buerger, Markus M. Nöthen, Piotr Lewczuk, Johannes Kornhuber, Harald Hampel, Ina Giegling, Oliver Goldhardt, Janine Diehl-Schmid, Victor Andrade, Michael MT. Heneka, Lutz Frölich, Jonathan Vogelgsang, Caroline Graff, Hakan Thonberg, Abbe Ullgren, Goran Papenberg, Jean-François Deleuze, Carole Dufouil, Michael Wagner, Frank Jessen, Henne Holstege, Cornelia van Duijn, Thibaud Lebouvier, Olivier Hannon, Ville Leinonen, Hilkka Soininen, Sanna-Kaisa Herukka, Vilmantas Giedraitis, Malin Löwenmark, Lena Kilander, Patricia Genius, Blanca Rodríguez, Emma S. Luckett, Arcadi Navarro, Amanda Cano, Marta Marquié, Kaj Blennow, Henrik Zetterberg, Alberto Lleo, Mercè Boada, Agustin Ruiz, Virginia Man-Yee Lee, Vivianna M. Van Deerlin, Yuetiva Deming, Sterling C. Johnson, Corinne D. Engelman, Pau Pastor, Ignacio Alvarez, Elaine R. Peskind, Amanda J. Heslegrave, Andrew J. Saykin, Kwangsik Nho, Suzanne E. Schindler, John C. Morris, David M. Holtzman, Eric McDade, Alan E. Renton, Alison Goate, Laura Ibanez, Matthias Riemenschneider, Marilyn S. Albert, Simon M. Laws, Tenielle Porter, Eleanor K. O’Brien, Leslie M. Shaw, Betty M. Tijms, Martin Ingelsson, Pieter Jelle Visser, Mikko Hiltunen, Kristel Sleegers, Craig W. Ritchie, Rebecca Sims, Michael Belloy, Jean-Charles Lambert, Natalia Vilor-Tejedor, Maria Victoria Fernández, Qingqin S. Li, Michael W. Nagle, Riccardo E. Marioni, Alfredo Ramirez, Lars Bertram, Sven J. van der Lee, Carlos Cruchaga

**Affiliations:** 1 Department of Psychiatry, Washington University School of Medicine, St. Louis, MO USA; 2 NeuroGenomics and Informatics Center, Washington University School of Medicine, St. Louis, MO USA; 3 Alzheimer Center Amsterdam, Neurology, Vrije Universiteit Amsterdam, Amsterdam UMC Location VUmc, Amsterdam, The Netherlands; 4 Amsterdam Neuroscience, Neurodegeneration, Amsterdam, The Netherlands; 5 Centre for Genomic and Experimental Medicine, Institute of Genetics and Cancer, The University of Edinburgh, Edinburgh, UK; 6 Human Genetics and Causal Biology, Eisai Center for Genetics-Guided Dementia Discovery, Eisai Inc, Cambridge, MA USA; 7 Division of Neurogenetics and Molecular Psychiatry, Department of Psychiatry and Psychotherapy, Faculty of Medicine and University Hospital Cologne, University of Cologne, Cologne, Germany; 8 Estudios en Neurociencias y Sistemas Complejos (ENyS), CONICET-HEC-UNAJ, Buenos Aires, Argentina; 9 Department of Psychiatry and Neurochemistry, Institute of Neuroscience and physiology, the Sahlgrenska Academy at the University of Gothenburg, Gothenburg, Sweden; 10 Barcelonaβeta Brain Research Center (BBRC), Pasqual Maragall Foundation, Barcelona, Spain; 11 Centre for Genomic Regulation (CRG), Barcelona Institute of Science and Technology (BIST), Barcelona, Spain; 12 Hospital del Mar Research Institute, Barcelona, Spain; 13 Lübeck Interdisciplinary Platform for Genome Analytics, University of Lübeck, Lübeck, Germany; 14 Ace Alzheimer Center Barcelona, Universitat Internacional de Catalunya, Barcelona, Spain; 15 Department of Neurology, Washington University School of Medicine, St. Louis, MO USA; 16 UK Dementia Research Institute at Cardiff, Cardiff, UK; 17 Vanderbilt Memory and Alzheimer’s Center, Vanderbilt University Medical Center, Nashville, TN USA; 18 Delft Bioinformatics Lab, Delft University of Technology, Delft, The Netherlands; 19 Neuropsychiatric Epidemiology Unit, Department of Psychiatry and Neurochemistry, Institute of Neuroscience and Physiology, the Sahlgrenska Academy, Centre for Ageing and Health (AGECAP) at the University of Gothenburg, Gothenburg, Sweden; 20 Department of Neuropsychiatry, Region Västra Götaland, Sahlgrenska University Hospital, Gothenburg, Sweden; 21 Reina Sofia Alzheimer Center, CIEN Foundation, ISCIII, Madrid, Spain; 22 Centro de Investigación Biomédica en Red de Enfermedades Neurodegenerativas (CIBERNED), Instituto de Salud Carlos III, Madrid, Spain; 23 Neurology Service, Marqués de Valdecilla University Hospital, Santander, Spain; 24 Department of Cognitive Disorders and Old Age Psychiatry, University Hospital Bonn, Bonn, Germany; 25 German Center for Neurodegenerative Diseases (DZNE), Bonn, Germany; 26 German Center for Neurodegenerative Diseases (DZNE), Berlin, Germany; 27 Charité–Universitätsmedizin Berlin, corporate member of Freie Universität Berlin, Humboldt-Universität zu Berlin, and Berlin Institute of Health, Institute of Psychiatry and Psychotherapy, Berlin, Germany; 28 Department of Biomedical Sciences, University of Antwerp, Antwerp, Belgium; 29 Complex Genetics of Alzheimer’s Disease Lab, VIB Center for Molecular Neurology, VIB, Antwerp, Belgium; 30 Univ. Lille, Inserm, CHU Lille, Institut Pasteur de Lille, LabEx DISTALZ-U1167-RID-AGE Facteurs de Risque et Determiants moléculaires des maladies liées au vieillissement, Lille, France; 31 Institute of Biomedicine, University of Eastern Finland, Kuopio, Finland; 32 Martin-Luther-University Halle-Wittenberg, University Clinic and Outpatient Clinic for Psychiatry, Psychotherapy and Psychosomatics, Halle (Saale), Germany; 33 LVR-University Hospital Essen, Department of Psychiatry and Psychotherapy, Medical Faculty, University of Duisburg-Essen, Essen, Germany; 34 Department of Geriatric Psychiatry, Central Institute for Mental Health Mannheim, Faculty Mannheim, University of Heidelberg, Heidelberg, Germany; 35 German Center for Neurodegenerative Diseases (DZNE), Magdeburg, Germany; 36 Institute of Cognitive Neurology and Dementia Research (IKND), Otto-von-Guericke University, Magdeburg, Germany; 37 Center for Cognitive Disorders, Department of Psychiatry and Psychotherapy, Technical University of Munich, TUM School of Medicine and Health, Munich, Germany; 38 Department of Psychiatry and Psychotherapy, University Medical Center Goettingen, Goettingen, Germany; 39 German Center for Neurodegenerative Diseases (DZNE), Goettingen, Germany; 40 Medical Science Department, iBiMED, Aveiro, Portugal; 41 Department of Neurology, UZ Leuven, Leuven, Belgium; 42 Department of Neurology, Universitair Ziekenhuis Brussel and Neuroprotection and Neuromodulation (NEUR) Research Group, Center for Neurosciences (C4N), Vrije Universiteit Brussel (VUB), Brussels, Belgium; 43 Institute of Human Genetics, University of Bonn, School of Medicine & University Hospital Bonn, Bonn, Germany; 44 Institute of Medical Biometry, Informatics and Epidemiology, University Hospital of Bonn, Bonn, Germany; 45 1st Department of Neurology, Medical school, Aristotle University of Thessaloniki, Thessaloniki, Greece; 46 1st Department of Neurology, Aiginition Hospital, Medical School, National and Kapodistrian University of Athens, Athens, Greece; 47 Depatment of Neurology, Columbia University, New York, NY USA; 48 Sant Pau Memory Unit, Institut de Recerca Sant Pau, Department of Neurology, Hospital de la Santa Creu I Sant Pau, Universitat Autònoma de Barcelona, Barcelona, Spain; 49 Department of Neurology, Hospital Universitario Donostia, San Sebastian, Spain; 50 Neurosciences Area, Instituto Biodonostia, San Sebastian, Spain; 51 Unitat de Genètica Molecular, Institut de Biomedicina de València-CSIC, Valencia, Spain; 52 Centro de Biología Molecular Severo Ochoa (UAM-CSIC), Madrid, Spain; 53 Instituto de Investigacion Sanitaria ‘Hospital la Paz’ (IdIPaz), Madrid, Spain; 54 Universidad Autónoma de Madrid, Madrid, Spain; 55 Alzheimer’s disease and other cognitive disorders unit. Service of Neurology. Hospital Clínic of Barcelona. Institut d’Investigacions Biomèdiques August Pi i Sunyer, University of Barcelona, Barcelona, Spain; 56 Laboratorio de Genética. Hospital Universitario Central de Asturias, Oviedo, Spain; 57 Instituto de Investigación Sanitaria del Principado de Asturias (ISPA), Av. del Hospital Universitario, Asturias, Spain; 58 Unidad de Trastornos del Movimiento, Servicio de Neurología y Neurofisiología. Instituto de Biomedicina de Sevilla (IBiS), Hospital Universitario Virgen del Rocío/CSIC/Universidad de Sevilla, Seville, Spain; 59 Unidad Clínica de Enfermedades Infecciosas y Microbiología. Hospital Universitario de Valme, Sevilla, Spain; 60 Depatamento de Especialidades Quirúrgicas, Bioquímica e Inmunología. Facultad de Medicina. Universidad de Málaga, Málaga, Spain; 61 Unitat Trastorns Cognitius, Hospital Universitari Santa Maria de Lleida, Lleida, Spain; 62 Institut de Recerca Biomedica de Lleida (IRBLLeida), Lleida, Spain; 63 Alzheimer Research Center & Memory Clinic, Andalusian Institute for Neuroscience, Málaga, Spain; 64 Maastricht University, Department of Psychiatry & Neuropsychologie, Alzheimer Center Limburg, Maastricht, the Netherlands; 65 Neurology department Erasmus MC University Medical Center, Rotterdam, The Netherlands; 66 Internal Medicine Department, Erasmus MC University Medical Center, Rotterdam, The Netherlands; 67 Genomics of Neurodegenerative Diseases and Aging, Human Genetics, Vrije Universiteit Amsterdam, Amsterdam UMC location VUmc, Amsterdam, The Netherlands; 68 German Center for Neurodegenerative Diseases (DZNE), Tübingen, Germany; 69 Section for Dementia Research, Hertie Institute for Clinical Brain Research and Department of Psychiatry and Psychotherapy, University of Tübingen, Tübingen, Germany; 70 German Center for Neurodegenerative Diseases (DZNE), Rostock, Germany; 71 Department of Psychosomatic Medicine, Rostock University Medical Center, Rostock, Germany; 72 Department of Psychiatry and Psychotherapy, Charité – Universitätsmedizin Berlin, Berlin, Germany; 73 Department of Psychiatry and Psychotherapy, Klinikum rechts der Isar Technical University Munich, Munich, Germany; 74 German Center for Neurodegenerative Diseases (DZNE Munich), Munich, Germany; 75 Department of Psychiatry and Psychotherapy, University Hospital, LMU Munich, Munich, Germany; 76 Munich Cluster for Systems Neurology (SyNergy) Munich, Munich, Germany; 77 Ageing Epidemiology Research Unit, School of Public Health, Imperial College London, London, UK; 78 Institute for Stroke and Dementia Research (ISD), University Hospital, LMU Munich, Munich, Germany; 79 Institute of Human Genetics; University of Bonn, School of Medicine & University Hospital Bonn, Bonn, Germany; 80 Department of Psychiatry and Psychotherapy, Universitätsklinikum Erlangen, and Friedrich-Alexander Universität Erlangen-Nürnberg, Erlangen, Germany; 81 Department of Neurodegeneration Diagnostics, Medical University of Białystok, Białystok, Poland; 82 Sorbonne University, Alzheimer Precision Medicine (APM), AP-HP, Pitié-Salpêtrière Hospital, Boulevard de l’hôpital, Paris, France; 83 Division of General Psychiatry, Dept. of Psychiatry and Psychotherapy, Medical University of Vienna, Vienna, Austria; 84 Department of Neurodegenerative Diseases and Geriatric Psychiatry, University Hospital Bonn, Medical Faculty, Bonn, Germany; 85 Department of Psychiatry, Harvard Medical School McLean Hospital, Belmont, MA USA; 86 Division of Neurogeriatrics, Department NVS, Karolinska Institutet, Center for Alzheimer Research, Stockholm, Sweden; 87 Unit for Hereditary Dementias, Karolinska University Hospital-Solna, Stockholm, Sweden; 88 Aging Research Center, Department of Neurobiology, Care Sciences and Society, Karolinska Institutet and Stockholm University, Stockholm, Sweden; 89 Université Paris-Saclay, CEA, Centre National de Recherche en Génomique Humaine, Evry, France; 90 Inserm, Bordeaux Population Health Research Center, UMR 1219, Univ. Bordeaux, ISPED, CIC 1401-EC, Univ Bordeaux, Bordeaux, France; 91 CHU de Bordeaux, Pole santé publique, Bordeaux, France; 92 Department of Psychiatry and Psychotherapy, Faculty of Medicine and University Hospital Cologne, University of Cologne, Cologne, Germany; 93 Cluster of Excellence Cellular Stress Responses in Aging-associated Diseases (CECAD), University of Cologne, Cologne, Germany; 94 Department of Neurosciences, Leuven Brain Institute, KU Leuven, Leuven, Belgium; 95 Department of Epidemiology, ErasmusMC, Rotterdam, The Netherlands; 96 Nuffield Department of Population Health, University of Oxford, Oxford, UK; 97 Centre for Artificial Intelligence in Precision Medicine, University of Oxford, Oxford, UK; 98 Univ.Lille, Inserm, CHULille, Lille Neuroscience & Cognition, UMR-S1172, Lille, France; 99 Université de Paris, EA 4468, APHP, Hôpital Broca, Paris, France; 100 Department of Neurosurgery, Kuopio University Hospital and Institute of Clinical Medicine-Neurosurgery, University of Eastern Finland, Kuopio, Finland; 101 Department of Neurology, Institute of Clinical Medicine, University of Eastern Finland, Kuopio, Finland; 102 Department of Neurology, NeuroCenter, Kuopio University Hospital, Kuopio, Finland; 103 Department of Public Health and Caring Sciences/Clinical Geriatrics, Uppsala University, Uppsala, Sweden; 104 Department of Radiology and Nuclear Medicine, Amsterdam UMC location VUmc, Amsterdam, Netherlands; 105 Amsterdam Neuroscience, Brain Imaging, Amsterdam, The Netherlands; 106 Laboratory for Cognitive Neurology, KU Leuven, Leuven, Belgium; 107 Laboratory for Complex Genetics, KU Leuven, Leuven, Belgium; 108 Institució Catalana de Recerca i Estudis Avançats (ICREA), Barcelona, Spain; 109 Department of Experimental and Health Sciences, Institute of Evolutionary Biology (CSIC-UPF), Universitat Pompeu Fabra, Barcelona, Spain; 110 Clinical neurochemistry Laboratory, Sahlgrenska University Hospital, Mölndal, Sweden; 111 Paris Brain Institute, ICM, Pitié-Salpêtrière Hospital, Sorbonne University, Paris, France; 112 UK Dementia Research Institute at UCL, London, UK; 113 Wisconsin Alzheimer’s Disease Research Center, University of Wisconsin School of Medicine and Public Health, University of Wisconsin-Madison, Madison, WI USA; 114 Glenn Biggs Institute for Alzheimer’s & Neurodegenerative Diseases and Department of Microbiology, Immunology and Molecular Genetics, Long School of Medicine, University of Texas Health Science Center, San Antonio, TX USA; 115 Department of Pathology and Laboratory Medicine, Perelman School of Medicine at the University of Pennsylvania, Philadelphia, PA USA; 116 Department of Medicine, School of Medicine and Public Health, University of Wisconsin-Madison, Madison, WI USA; 117 Department of Population Health Sciences, School of Medicine and Public Health, University of Wisconsin-Madison, Madison, WI USA; 118 Unit of Neurodegenerative diseases, Department of Neurology, University Hospital Germans Trias i Pujol and The Germans Trias i Pujol Research Institute (IGTP) Badalona, Barcelona, Spain; 119 Veterans Affairs Northwest Mental Illness Research, Education, and Clinical Center, Seattle, WA USA; 120 Department of Psychiatry and Behavioral Sciences, University of Washington, Seattle, WA USA; 121 Department of Neurodegenerative Disease, UCL Institute of Neurology, London, UK; 122 Indiana Alzheimer’s Disease Research Center, Department of Radiology and Imaging Sciences, Indiana University School of Medicine, Indianapolis, Indiana, USA; 123 Department of Genetics, Washington University School of Medicine, St. Louis, MO USA; 124 Hope Center for Neurological Disorders, Washington University School of Medicine, St. Louis, MO USA; 125 Ronald M. Loeb Center for Alzheimer’s Disease, Icahn School of Medicine at Mount Sinai, New York, NY USA; 126 Department of Genetics and Genomic Sciences, Icahn School of Medicine at Mount Sinai, New York, NY USA; 127 Nash Family Department of Neuroscience, Icahn School of Medicine at Mount Sinai, New York, NY USA; 128 Department of Psychiatry, Saarland University, Homburg, Germany; 129 Department of Neurology, Johns Hopkins University School of Medicine, Baltimore, MD USA; 130 Centre for Precision Health, School of Medical Health Sciences, Edith Cowan University, Joondalup, WA Australia; 131 Krembil Brain Institute, University Health Network, Toronto, ON Canada; 132 Tanz Centre for Research in Neurodegenerative Diseases, Departments of Medicine and Laboratory Medicine & Pathobiology, University of Toronto, Toronto, ON Canada; 133 Department of Public Health and Caring Sciences, Molecular Geriatrics, Rudbeck Laboratory, Uppsala University, Uppsala, Sweden; 134 Department of Neurology, Alzheimer Center Amsterdam, Amsterdam Neuroscience, Vrije Universiteit Amsterdam, Amsterdam UMC, VUmc, Amsterdam, the Netherlands; 135 Department of Neurobiology, Care Sciences and Society, Division of Neurogeriatrics, Karolinska Institutet, Stockholm, Sweden; 136 Alzheimer Center Limburg, School for Mental Health and Neuroscience, Maastricht University, Maastricht, the Netherlands; 137 Scottish Brain Sciences, Scottish Gas Murrayfield Stadium, Edinburgh, UK; 138 School of Medicine, University of St Andrews, St Andrews, UK; 139 Division of Psychological Medicine and Clinical Neuroscience, School of Medicine, Cardiff University, Cardiff, UK; 140 Department of Human Genetics, Radboud University Medical Center, Nijgmegen, the Netherlands; 141 Neuroscience, Janssen Research & Development LLC, Titusville, NJ USA; 142 JRD Data Science, Janssen Research & Development LLC, Titusville, NJ USA; 143 CHDI Management Inc., Princeton, NJ USA; 144 Department of Psychiatry, Glenn Biggs Institute for Alzheimer’s and Neurodegenerative Diseases, San Antonio, TX USA; 145 Cologne Excellence Cluster on Cellular Stress Responses in Aging-Associated Disease (CECAD), University of Cologne, Cologne, Germany; 146 Section Genomics of Neurodegenerative Diseases and Aging, Human Genetics, Vrije Universiteit Amsterdam, Amsterdam UMC location VUmc, Amsterdam, The Netherlands

**Keywords:** Genome-wide association studies, Alzheimer's disease

## Abstract

Cerebrospinal fluid amyloid beta 42, total tau, and phosphorylated tau 181 are well accepted markers of Alzheimer’s disease. These biomarkers better reflect disease pathogenesis compared to clinical diagnosis. Here, we perform a genome wide association study meta-analysis including 18,948 individuals of European ancestry and identify 12 genome-wide significant loci across all three biomarkers, eight of them novel. We replicate the association of biomarkers with *APOE*, *CR1*, *GMNC/CCDC50* and *C16orf95/MAP1LC3B*. Novel loci include *BIN1* for amyloid beta and *GNA12, MS4A6A, SLCO1A2* with both total tau and phosphorylated tau 181, as well as additional loci on chr. 8, near *ANGPT1* and chr. 9 near *SMARCA2*. We also demonstrate that these variants have significant association with Alzheimer’s disease risk, disease progression and/or brain amyloidosis. The associated genes are implicated in lipid metabolism independent of *APOE*, coupled with autophagy and brain volume regulation driven by total tau and phosphorylated tau 181 dysregulation.

## Abstract (web summary)

The study identifies novel genetic variants linked to core cerebrospinal fluid Alzheimer’s Disease biomarkers using a genome-wide analysis, showing how endophenotypes can identify genes and disease mechanisms not captured by case-control studies.

## Introduction

Alzheimer’s disease (AD), a progressive neurodegenerative disease, is the most common cause of dementia worldwide^1^. Decades of research have identified mutations in *APP, PSEN1*, and *PSEN2* as the genetic basis for the familial form of AD^2,3^. For the more common sporadic AD and related dementias, genetic studies have identified more than 74 loci associated with the disease^4–8^. However, most of the genetic studies in AD are focused on clinical and symptomatic status, which can be biased due to the presence of presymptomatic subjects. Incorrect or mixed dementia diagnosis further add to these challenges. Endophenotypes are measurable traits that serve as intermediates between genetic variants and disease, providing a closer link to the disease processes involved. Since endophenotypes can often be more directly linked to genetic variation, their associations may be easier to interpret and better reflect the underlying mechanisms of disease.

Cerebrospinal fluid (CSF) Aβ42, total tau (t-tau) and phosphorylated tau (p-tau181) are critical and well-validated core AD biomarkers and endophenotypic measures^9^. A decrease in CSF Aβ42 and an increase in CSF t-tau and p-tau181 reflects the accumulation of aggregated β-amyloid into plaques the brain, with the later also capturing neurofibrillary tangle formation to some extent and can be detected before clinical symptoms. Both processes are hallmarks of AD pathology and contribute to disease mechanisms as proposed by the amyloid cascade theory^9,10^. Thus, identifying variants that modulate the levels of these biomarkers may help to prioritize genes linked with AD pathogenesis.

Previous GWAS focusing on these three CSF endophenotypes have highlighted important genetic associations^5,11,12^. Deming et al.^11^ identified five genome-wide significant loci associated with p-tau181 and three with Aβ42 from a GWAS of 3,146 participants across nine studies. They also demonstrated the role of these variants in disease risk and progression. Similarly, Jansen et al.^5^ identified two loci associated with Aβ42 and four loci associated with p-tau181. Only the *APOE* and *GMNC* loci (for p-tau181) were consistently identified in both studies. Therefore, further analysis with a larger subject size has the potential to identify novel associations and provide replication of previously reported findings.

Here, we performed a GWAS encompassing CSF measures of Aβ42, t-tau and p-tau181in 18,948 individuals, across 30 different studies. These include samples used in previous GWAS by Deming et al.^11^ and Jansen et al.^5^. We determined 12 unique significantly associated genetic variants across the three biomarkers and performed fine mapping and functional gene nomination of the loci. After adjusting for pleiotropy between the traits, many of the same genetic loci were identified suggesting robust association. In addition, we investigated the association of lead variants with AD progression, AD risk and other AD-related phenotypes. Finally, through pathway analysis, using genes linked to our variants, we investigated biological processes associated with these variants influencing our CSF biomarkers of interest.

## Results

### Meta-analysis replicates previously reported associations for CSF Aβ42, t-tau, and p-tau181

**Fig. 1:**
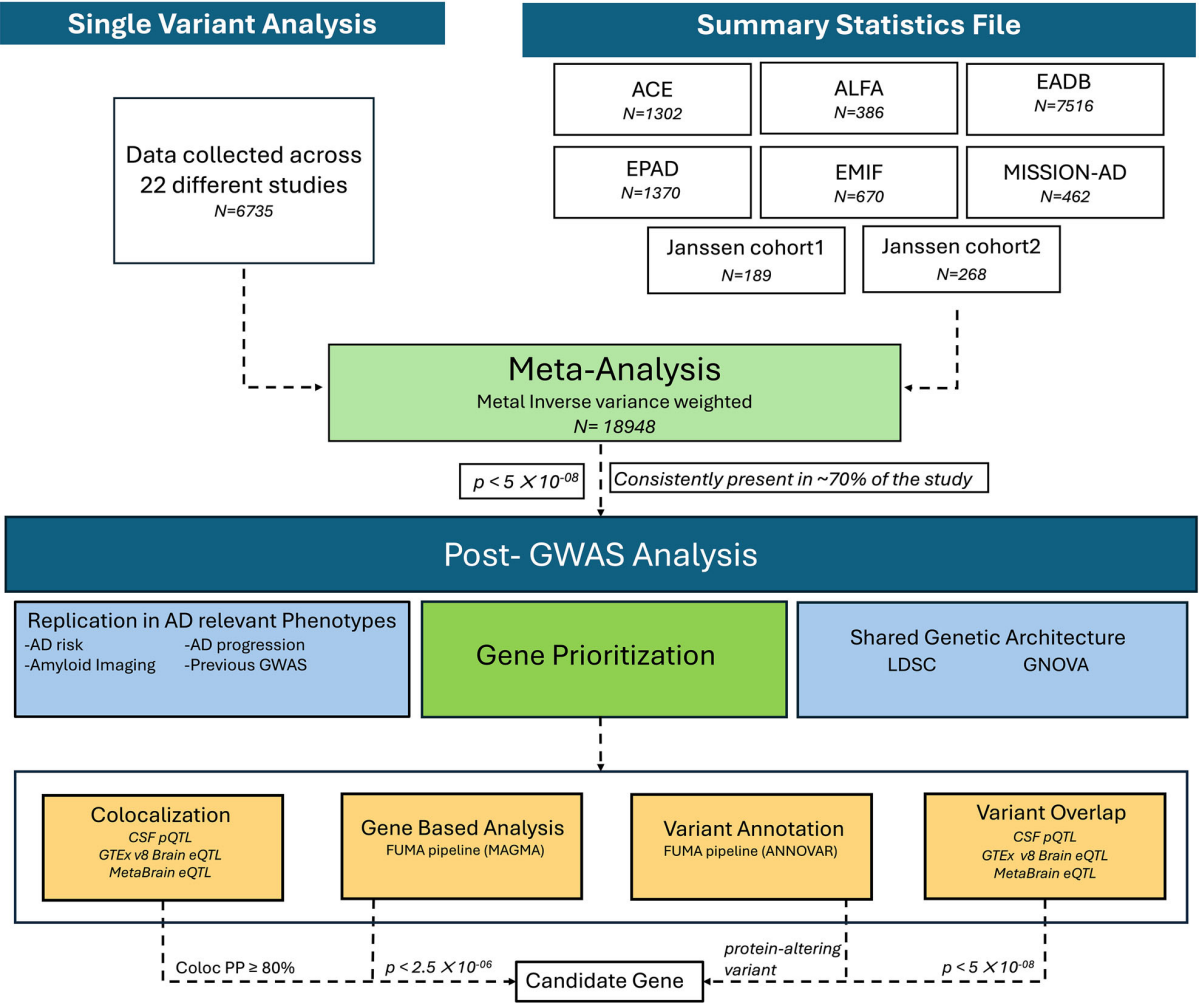
Schematic workflow of study design. We meta-analyzed data across 30 different studies. Raw biomarker and genetic data were collected from 22 cohorts for 6785 unique samples. Summary statistics were available from remaining sites. Consistent data preparation approach applied across all. Meta-analysis was performed using results from each individual GWAS using METAL. Significance defined *P* < 5 ⨉ 10^-08^. We identified replication in relevant AD traits for significant variants and checked genetic correlation with different traits. For variants that pass significance threshold, gene prioritization was done based on evidence from colocalization with quantitative trait loci (QTL) and gene/variant based functional analysis. ACE Ace Alzheimer Center Barcelona, ALFA Alzheimer’s and Families study, EADB European Alzheimer’s and Dementia Biobank, EMIF European Medical Information Framework, EPAD The European Prevention of Alzheimer’s Dementia, AD Alzheimer’s disease, LDSC LD Score Regression, GNOVA Genetic Covariance Analyzer.

**Fig. 2:**
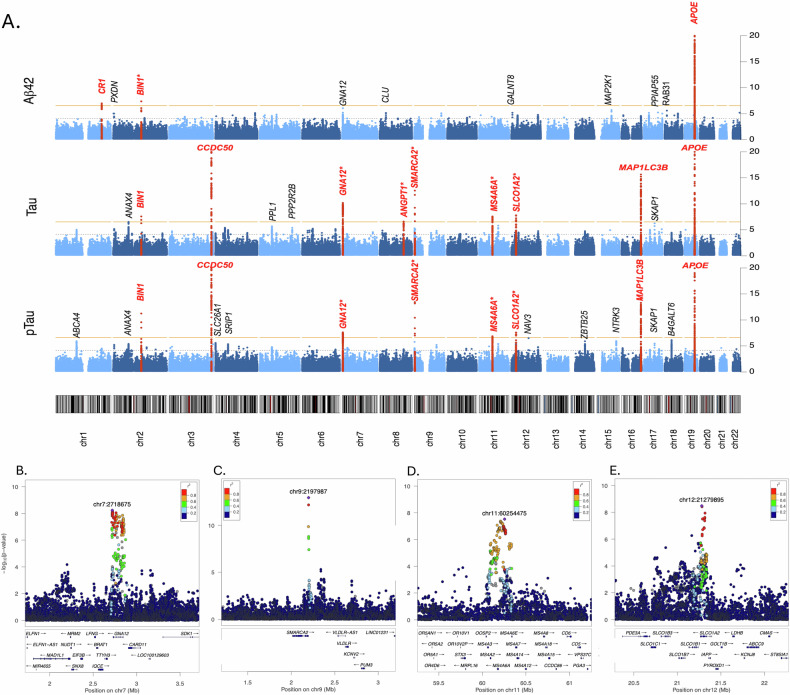
Association plots from meta-analysis. **A** Manhattan plot showing negative log10-transformed P values from the meta-analysis of all three CSF biomarkers. The horizontal lines represent the genome-wide significance threshold, *P* = 5 × 10^−8^ (orange) and suggestive threshold, *P* = 1×10^−5^ (gray). The loci highlighted red were those that passed genome wide threshold in each of these analyses. The genes represent genes prioritized by functional and conditional analysis for genome wide significant loci or the closet genes (shown in black) for those variants that were close to the threshold but did not pass significance. “*” denotes novel association for the trait. Locuszoom plots of novel loci associated with pTau near *GNA12* (**B**), SMARCA2 (**C**), within *MS4A* gene family (**D**) and *SLCO1A2* (**E**). Aβ42 meta-analysis (https://wustl.box.com/s/nfexw54o37smdq84lz1inpqduqcf7ofa), tau meta-analysis (https://wustl.box.com/s/pydeqc87yke2ejvve5mrh9quyaikgq2p) and p-tau181 meta-analysis (https://wustl.box.com/s/nmyjzql5awxu7qu57m33rkcvq1w3njj8).

We associated individual-level genetic data with CSF biomarker levels in 18,948 unrelated European ancestry individuals from 30 different studies (Table 1). Detailed study workflow is presented in Fig. 1. We identified 12 unique genome-wide significant variants across Aβ42, t-tau, and p-tau181 including one novel association for Aβ42, five for t-tau, and four for p-tau181 (Fig. 2A; Table 2, Supplementary Figs. 1–7). The genomic inflation lambda(λ) for the meta-analysis summary statistics was 1.07–1.08 (Supplementary Fig. 8). The LD Score regression intercept was approximately equal to the genomic control inflation: 1.04 for Aβ42 and 1.055 for both t-tau and p-tau181, suggesting minimal inflation due to confounding factors.

**Table 1:** Demographic information of subjects with CSF biomarkers used in meta-analysis

Cohort	Platform	N	Average Age (SD)	%Male	Average Aβ42	Average t-tau	Average ptau181	%Cases	%Controls
ADNI	xMAP	1145	73.59 ( ± 7.24)	55.9	172.84	90.8	39.54	71.27	28.65
AIBL	Innotest	209	73.2 ( ± 6.14)	50.72	810.77	285.73	57.86	27.75	72.25
BIOCARD	NA	167	60.02 ( ± 7.69)	41.32	417.73	69.33	36.07	2.40	94.61
Blennow	NA	56	83.2 ( ± 10.21)	26.79	550.91	679.54	74.14	58.93	41.97
Barcelona-1	ELISA	279	67.78 ( ± 8.33)	49.46	927.12	472.28	70.17	24.73	1.43
DIAN	Lumipulse	188	37.26 ( ± 10.12)	42.55	679.02	408.41	58.73	22.34	77.66
DOD	Elecsys	101	68.42 ( ± 3.59)	99.01	1216.85	218.96	19.11	27.72	69.31
HB	NA	100	67.35 ( ± 9.25)	55	76.08	84.95	NA	100	0
Lleo	NA	122	63.72 ( ± 9.68)	32.79	642.59	385.72	56.68	31.97	54.92
London	NA	253	68.67 ( ± 8.96)	53.36	387.66	97.32	41.93	63.36	8.30
MAP	Lumipulse	1024	69.63 ( ± 9.44)	47.56	769.38	387.31	50.21	29.10	70.80
MARS	NA	103	64.69 ( ± 8.67)	59.22	834.88	222.38	37.96	84.47	15.53
MAYO	NA	425	78.21 ( ± 6.24)	60.24	330.76	98.65	21.65	18.82	79.53
Moli	NA	228	64.68 ( ± 8.01)	38.16	554.34	471.08	79.05	67.54	21.93
NACC	Multiple	469	71.37 ( ± 9.00)	46.7	334.1	291.01	56.78	42.64	29.85
PPMIS	NA	813	62.17 ( ± 9.67)	61.87	927.67	176.67	15.52	47.60	21.89
SWEDEN	NA	289	75.15 ( ± 7.66)	37.02	262.94	783.72	106.01	100	0
UPENN	Luminex	46	72.13 ( ± 8.27)	60.87	141.84	118.21	40.74	10.87	2.17
UW	NA	343	62.41 ( ± 15.96)	51.31	141.13	62.95	56.71	34.40	59.18
VMAP	NA	135	72.39 ( ± 6.42)	68.89	704.9	424.59	61.87	37.04	52.59
WiscADRC	NA	214	64.11 ( ± 9.60)	39.25	669.25	375.87	49.6	22.89	76.17
Zetter	NA	76	73.45 ( ± 4.14)	50	619.64	431.59	76.29	39.47	23.68
ACE	Lumipulse; ELISA	1302	72.51 ( ± 8.98)	42.63	767.68	515.87	79.6	28.34	67.66
ALFA+	NeuroToolKit	386	61.14 ( ± 4.633)	38.34	1315.43	195.87	15.87	NA	100
EADB	Multiple	7516	70.96 ( ± 7.65)	46.26	637.21	561.67	75.01	60.36	38
MISSION-AD	Lumipulse	462	71.71 ( ± 7.22)	53.90	739.20	500.39	67.87	100	0
EMIF	NA	670	69.29 ( ± 8.3)	47.9	309.13	NA	NA	23.3	18.2
EPAD		1370	65.63 ( ± 7.33)	44.05	1380.84	225.73	19.76	0	100
Janssen Cohort 1	NA	189	69.42 ( ± 6.33)	45	451.29	546.46	69.16	100	0
Janssen Cohort 2	NA	268	71.27 ( ± 8.73)	48.30	661.55	707.17	104.91	100	0

*NA* Not available; *N* Number of subjects; *Age* Age at CSF draw in years; *SD* Standard Deviation.

Cases include AD cases, MCI or PD cases (if known PD cohort) only. Average Biomarker level in pg/ml or similar unit. Details on individual cohort is available as supplementary material. EADB included 12 individual cohorts; detail of which is provided in Supplementary Data 20.

**Table 2:** Significant Meta-analysis variants for CSF phenotypes, with results from most recent AD risk, AD progression and Amyloid imaging studies. *P*-values are unadjusted values from each summary statistic

								Metaanalysis		AD Risk^4^		AD Progression^18^		Amyloid Imaging^17^	
MarkerName	rsID	Trait	EA	MAF	Closest Gene	Nominated gene	Status	Effect size	P	OR	P	Effect size	P	Effect size	P
chr1:207625371:T:G	rs10779336	Aβ42	T	0.198	*CR1*	*CR1*	Novel	-0.073	3.49E-08	1.127	4.69E-31	4.08E-05	4.10E-01	0.097	4.75E-10
chr2:127135234:C:T	rs6733839	Aβ42	T	0.394	*BIN1*	*BIN1*	Reported	-0.06	8.29E-09	1.184	6.48E-90	1.18E-04	3.89E-03	0.046	2.66E-04
chr19:44908684:T:C	rs429358	Aβ42	C	0.228	*APOE*	*--*	Reported	-0.589	1.90e-674	NA	NA	4.77E-05	2.73E-01	0.62	1.81e-416
chr2:127135234:C:T	rs6733839	t-tau	T	0.394	*BIN1*	*BIN1*	Reported	0.06	5.24E-09	1.184	6.48E-90	1.18E-04	3.89E-03	0.046	2.66E-04
chr3:190953006:C:T	rs35327527	t-tau	T	0.369	*GMNC*	*CCDC50*	Reported	0.151	3.45E-48	1.009	3.00E-01	2.07E-05	6.17E-01	0.031	1.43E-02
chr7:2761908:G:A	rs798490	t-tau	A	0.298	*AMZ1-GNA12*	*GNA12*	Novel	-0.074	1.87E-11	1.02	2.51E-02	1.93E-05	6.64E-01	0.017	2.15E-01
chr8:107381731:A:G	rs1654723	t-tau	A	0.411	*ANGPT1*	*--*	Novel	0.056	4.41E-08	1.005	5.85E-01	5.89E-05	1.56E-01	-0.008	4.90E-01
chr9:2197987:A:G	rs57263785	t-tau	G	0.248	*SMARCA2*	*--*	Novel	0.087	1.12E-13	1.014	1.54E-01	6.20E-06	0.8963	0.033	2.25E-02
chr11:60207692:G:A	rs7928895	t-tau	A	0.393	*MS4A4E*	*MS4A6A*	Novel	-0.06	5.35E-09	0.928	2.08E-19	-8.97E-05	3.45E-02	-0.02	1.02E-01
chr12:21279895:C:T	rs11045930	t-tau	T	0.288	*SLCO1A2*	*SLCO1A2*	Novel	0.065	3.26E-09	1.01	2.86E-01	4.37E-05	3.23E-01	-0.004	7.98E-01
chr16:87199910:G:A	rs4843552	t-tau	G	0.409	*C16orf95*	*MAP1LC3B*	Reported	0.085	1.30E-16	0.982	3.21E-02	-4.55E-05	2.73E-01	0.003	8.29E-01
chr19:44908684:T:C	rs429358	t-tau	C	0.228	*APOE*	*--*	Reported	0.301	5.40E-161	NA	NA	4.77E-05	2.73E-01	0.62	1.81e-416
chr2:127135234:C:T	rs6733839	p-tau181	T	0.394	*BIN1*	*BIN1*	Reported	0.073	2.28E-12	1.184	6.48E-90	1.18E-04	3.89E-03	0.046	2.66E-04
chr3:190953006:C:T	rs35327527	p-tau181	T	0.369	*GMNC*	*CCDC50*	Reported	0.153	1.43E-47	1.009	3.00E-01	2.07E-05	6.17E-01	0.031	1.43E-02
chr7:2718675:A:G	rs798560	p-tau181	G	0.298	*AMZ1*	*GNA12*	Novel	−0.065	5.81E-09	1.029	1.24E-03	3.73E-05	3.95E-01	0.017	2.02E-01
chr9:2197987:A:G	rs57263785	p-tau181	G	0.248	*SMARCA2*	*--*	Novel	0.091	2.06E-14	1.014	1.54E-01	6.20E-06	0.8963	0.033	2.25E-02
chr11:60254475:G:A	rs1582763	p-tau181	A	0.365	*MS4A4E*	*MS4A6A*	Novel	-0.059	2.94E-08	0.918	1.65E-24	-6.30E-05	1.36E-01	-0.023	6.42E-02
chr12:21279895:C:T	rs11045930	p-tau181	T	0.288	*SLCO1A2*	*SLCO1A2*	Novel	0.065	6.24E-09	1.01	2.86E-01	4.37E-05	3.23E-01	-0.004	7.98E-01
chr16:87199910:G:A	rs4843552	p-tau181	G	0.409	*C16orf95*	*MAP1LC3B*	Reported	0.081	7.28E-15	0.982	3.21E-02	-4.55E-05	2.73E-01	0.003	8.29E-01
chr19:44908684:T:C	rs429358	p-tau181	C	0.228	*APOE*		Reported	0.318	3.27E-174	NA	NA	4.77E-05	2.73E-01	0.62	1.81e-416

Significance level defined as *p *< 5E-08 for GWAS study and *p* < 0.05 for AD risk and progression. Status denotes if the loci is novel or has been reported previously for the biomarker. Direction of effect has been harmonized across all summary statistics in reference to the listed effect allele.

*EA* effect allele, *MAF* minor allele frequency.

The *APOE* ε4 variant (rs429358) was the most significant variant identified across all three endophenotypes (Fig. 2A; Table 2). In addition to the *APOE* ε4 variant, rs7412, which determines *APOE* ε2 allele, and rs5117 mapped to *APOC1* within the *APOE* region were identified as independent signals for the three biomarkers.

We also replicated the previously reported *CR1* association with CSF Aβ42 (Fig. 2; Supplementary Fig. 1). The rs10779336[T] variant in the *CR1* gene region was associated with CSF Aβ42 levels and was in high linkage disequilibrium (LD) with the lead variant from Jansen et al.^5^ study (rs4844610[A], *r*^2^ = 0.95, Supplementary Data 1, 2). For CSF t-tau and p-tau181, the previously reported *GMNC* locus on chromosome 3 and the *C16orf95* locus on chromosome 16 were also genome wide significant in this study (Supplementary Figs. 2, 4, 5, 7)^5,11^. The lead variant rs35327527 within the *GMNC* locus from the meta-analysis, is in high LD with previously reported variants (rs35055419 *r*^2^ = 0.98; rs9877502 *r*^2^ = 0.99) and had a consistent direction of effect on CSF t-tau and p-tau181 (Supplementary Data 1). Similarly, the lead variant rs4843559 within the *C16orf95* locus also had high LD with the previously reported variant associated with p-tau181 in the region (*r*^2^ = 0.82). Lead variants reported in the previous Jansen et al.^5^ study all showed a consistent direction of effect and were significant in our analysis (Supplementary Data 2).

### Distinct and overlapping novel associations identified for each CSF endophenotype

In addition to the previously reported variants, we identified novel genomic loci associated with the CSF biomarkers: one locus for Aβ42, five loci for t-tau, and four loci for p-tau181 (Table 2, Fig. 2, Supplementary Figs. 1–7). Outside of a sub-threshold p-tau181 association at 8q23.1, all other p-tau181 loci were shared with t-tau.

The variant rs6733839[T] in the *BIN1* locus was significantly associated with CSF Aβ42 (Effect size = −0.060, SE = 0.011, *p* = 8.29 × 10^−09^, *I*^2^ = 0; Table 2; Supplementary Fig. 1). Although *BIN1* is a well-known AD risk locus and has been shown to affect CSF t-tau and p-tau181 levels, its direct association with CSF Aβ42 has not been reported previously^4–6,8^. This variant was also found to influence t-tau and p-tau181 levels in the meta-analysis with consistent direction of effects as in previous studies (Effect size_t-tau_ = 0.060, SE_t-tau_ = 0.010, *p*_t-tau_ = 5.24 × 10^−09^, *I*^2^_t-tau_ = 48.1; Effect size_ptau181_ = 0.073, SE_p-tau181_ = 0.010, *p*_ptau181_ = 2.28 × 10^−12^, *I*^2^ = 10.8; Table 2; Supplementary Figs. 2, 5; Supplementary Data 1).

The variant rs798490[A] (MAF = 0.298), located in the 7p22.3 region near *GNA12* and *AMZN1*, was associated with t-tau (Effect size = -0.074, SE = 0.011, *p* = 1.87 × 10^−11^, *I*^2^ = 0, Supplementary Fig. 2) whereas rs798560[G] (MAF = 0.298) within the same gene region, and in LD with the variant associated with t-tau (*r*^2^ = 0.810), was associated with p-tau181 levels (Effect size = −0.065, SE = 0.011, *p* = 5.81 × 10^−09^, *I*^2^ = 0; Supplementary Fig. 6). An additional novel locus on 9p24.3 (rs57263785[G] near *SMARCA2*) showed significant associations with both t-tau and p-tau181 (Effect size_t-tau_ = 0.087, SE_t-tau_ = 0.012, *p*_t-tau_ = 1.12 × 10^−13^, *I*^2^_t-tau_ = 0; Effect size_p-tau181_ = 0.091, SE _p-tau181_ = 0.012, *p*_p-tau181_ = 2.06 × 10^−14^, *I*^2^_p-tau181_ = 0; Table 2, Supplementary Figs. 3, 6). The *MS4A* gene region on chromosome 11 is an important AD risk locus and has been well documented to modulate soluble triggering receptor expressed on myeloid cells 2 (sTREM2) in CSF^13^. We observed two distinct variants that were in LD (*r*^2^ = 0.795) associated with t-tau and p-tau181, in this region (rs7928895[A]: Effect size_t-tau_ = -0.060, SE_t-tau_ = 0.010, *p*_t-tau_ = 5.35 × 10^−09^, *I*^2^_t-tau_ = 0; rs1582763[A]: Effect size_p-tau181_ = −0.059, SE _p-tau181_ = 0.011, *p*_p-tau181_ = 2.94 × 10^−08^, *I*^2^_p-tau181_ = 0; Supplementary Figs. 4, 7). Another novel association for both t-tau and p-tau181 was identified on chromosome 12 (rs11045930[T] within *SLCO1A2;*Effect size_t-tau_ = 0.065, SE_t-tau_ = 0.011, *p*_t-tau_ = 3.26 × 10^−09^, *I*^2^_t-tau_ = 0; Effect size_p-tau181_ = 0.065, SE_p-tau181_ = 0.011, *p*_p-tau181_ = 6.24 × 10^−09^, *I*^2^_p-tau181_ = 0; Supplementary Figs. 4, 7). Finally, a novel genetic association on 8q23.1 near *ANGPT1* and unique to t-tau was also identified (rs1654723[A]: Effect size = 0.056, SE_t-tau_ = 0.010, *p* = 4.41 × 10^−08^, *I*^2^_t-tau_ = 0, MAF = 0.411; Supplementary Fig. 3). The variant showed a suggestive association with p-tau181 (*p* = 6.17 × 10^−06^). No additional independent signals were observed in any of the novel loci. The H1/H2 haplotype in the chromosome 17 that contains *MAPT* gene, which encodes tau, did not have any association with tau or ptau-181 (Supplementary Fig. 9).

### Shared genetic influences across traits

We then analyzed all three biomarkers together using Multi-Trait Analysis of GWAS (MTAG) to identify additional signals that may have been missed by single-trait GWAS^14^. Orthogonal pairwise conjunctional false discovery rate (pleioFDR) based approach was also used in combination with MTAG to identify true pleiotropic signals^15,16^.

Prior to pleiotropy test, analysis of genetic correlation between the traits showed a highly significant genetic correlation between t-tau and p-tau181 (*p* = 6.99 × 10^−303^), however, correlation of these two traits with Aβ42 was not significant (p_Aβ42 vs t-tau_ = 0.25 and p_Aβ42 vs p-tau181_ = 0.27). Phenotypic correlation, assessed using available individual level data, were in expected direction with a significant negative correlation of Aβ42 with both t-tau and p-tau (Supplementary Fig. 10). The median phenotypic correlation between the traits pair were −0.28 and −0.22 respectively. Similarly, we observed significant positive phenotypic correlation between t-tau and p-tau181 with a median correlation of 0.91 and an average of 0.83 (Supplementary Fig. 10).

We identified seven genome-wide significant loci for Aβ42, nine for t-tau and eight for p-tau181 after combining results from both MTAG and pleioFDR (Supplementary Data 3, Supplementary Fig. 11). Association of chromosome 3, 7, 9, 12, and 16 were found for both t-tau and p-tau181. These loci were consistent with association identified in meta-analysis. Association of *APOE* with CSF Aβ42 was also identified but its association with t-tau and p-tau was not observed using MTAG. This could potentially be due to the very significant association of this locus with Aβ42 (*p* = 10^−600^ vs *p* = 10^−100^ in t-tau/p-tau181 from METAL) resulting in MTAG treating it as trait-specific association or because the effect of *APOE* on t-tau and p-tau181 is not independent of Aβ42. However, previous research has shown that although part of *APOE*-t-tau/ptau181 interaction is mediated through Aβ42, some variants within the region affect tau/ptau-181 independently^12^. Associations of chromosome 7, 9 and 16 with Aβ42 were novel but were same as those identified for t-tau and p-tau-181. pleio-FDR showed significant pleiotropy of these loci between Aβ42 and t-tau and ptau181 (chromosome 7 p_Aβ42vst-tau_ = 8.71 × 10^−05^, p_Aβ42vspTau181_ = 9.14 × 10^−05^; chromosome 9 p_Aβ42vst-tau_ = 0.031, p_Aβ42vspTau181_ = 0.033; chromosome 16 p_Aβ42vst-tau_ = 1.25 × 10^−04^, p_Aβ42vspTau181_ = 1.31 × 10^−04^; Supplementary Data 3). These findings suggest that there may be some interaction between Aβ42 and t-tau/ptau181 in these loci.

In addition to these known association, there were some novel loci identified by MTAG. These included: a locus on chromosome 2 near *GMCL1*, a locus on chromosome 5 near *MTREX* and a locus on chromosome 6 near *PLEKHG1* associated with Aβ42, t-tau and p-tau181. An additional locus near *PPP2R2B* in chromosome 5 was associated with t-tau only. For loci that were unique to MTAG, we investigated the effect of the lead variant in these loci on other AD-related traits using publicly available resources and conducted follow-up colocalization analysis for any loci that showed significant effect^4,17,18^. The chromosome 6 variant associated with all three biomarkers and the chromosome 5 variant associated with t-tau only were not associated with any of the AD-related traits. However, both chromosome 2 and 5 variants were nominally associated with brain amyloidosis (*p*_chr2_ = 0.023; 0.016; *p*_chr5_ = 0.022). Colocalization analyses were then performed for these two loci with CSF protein quantitative trait loci (pQTLs) and brain expression quantitative trait loci (eQTLs) to identify potential functional genes (Supplementary Data 4). The signal at the chromosome 2 locus colocalized with eQTLs for either *GMCL1*, *SNRNP27* or *ANXA4* depending on brain region. In addition, associations with t-tau and p-tau181 also colocalized with CSF pQTL for *ANXA4* (Supplementary Data 4). Because *ANXA4* had colocalization evidence from both GTEx v8 Brain eQTL and CSF pQTL for tau-181 and ptau-181, we nominate the gene as most likely functional gene for the locus. *ANAX* family of genes have been shown to increase in brain of AD patients and play a role in Aβ clearance and also contribute to the localization of tau in the axon^19,20^. We did not observe colocalization of Aβ42 association with pQTL for *ANXA4* (PP.H4 = 0.536), thus *GMCL1* is most likely the functional for Aβ42 association at chromosome 2 based on evidence of colocalization from GTEx v8 Brain eQTL and MetaBrain. Overexpression of *GMCL1* has been linked to certain type of cancers however there is no direct evidence linking it to AD^21^. In light of these findings, we hypothesize that the chromosome 2 locus has trait specific regulatory mechanisms. Similarly, chromosome 5 locus colocalized with brain eQTLs for *CCNO* and *MCIDAS* gene. In t-tau and ptau-181, colocalization was observed in both GTEx v8 Brain eQTL and MetaBrain eQTL but in Aβ42 this was only found in GTEx v8 Brain eQTL. *CCNO* had evidence from both GTEx and MetaBrain; thus, *CCNO* is likely the functional of the two genes. The *CCNO* gene has been reported to control other genes in AKT signaling cascade which is an important disease mechanism in AD^20,22^.

Overall, after accounting for pleiotropy between the biomarkers, we observed signals previously identified in our meta-analysis as well as few novel signals that point to additional disease mechanism.

### Cross-ancestry comparisons

We then tested whether the variants identified in participants of European ancestry were also associated in other populations.

The analysis in the non-European population revealed the association of *APOE* locus (rs429358) with Aβ42 (Effect size = −0.695, SE = 0.079, *p* = 8.02 × 10^−17^; Supplementary Data 1, Supplementary Fig. 12). The *APOE* variant, observed to be associated with both t-tau and p-tau181 in main analysis, did not pass genome-wide significance but showed trends toward association (*p*_t-tau_ = 3.17 × 10^−03^, *p*_p-tau181_ = 6.25 × 10^−04^). Similarly, the variant rs10779336 in the *CR1* locus on chromosome 1 (*p* = 1.24 × 10^−02^ for Aβ42) and the variant rs6733839 in the *BIN1* locus (*p*_t-tau_ = 5.53 × 10^−02^; *p*_p-tau181_ = 7.42 × 10^−02^) had associations with *p *< 0.05 (Supplementary Data 1). The trend of association in signals observed in this exploratory analysis, despite the limited sample size, suggests potentially meaningful genetic effects in non-European samples as well that need further investigation in larger cohorts.

### Diagnosis stratified analysis

We also performed stratified GWAS in Alzheimer disease (AD) and healthy control (CO) to see if any meta-analysis finding were driven by either diagnosis group. A GWAS including an interaction with status was also performed to assess effect of the diagnosis status on the biomarkers.

In the AD-specific analyses, significant associations were found for chromosome 19 for Aβ42, t-tau and p-tau181. Within CO, in addition to the chromosome 19 association with the three biomarkers, association of *GMNC* locus on chromosome 3 with t-tau and p-tau181 were also identified (Supplementary Fig. 13, Supplementary Data 5). Even if this signal was not genome-wide in AD cases, it showed a suggestive association and additional analyses indicated that the effect size of this variant in AD cases or controls is not significantly different. We also identified some novel loci when stratified by disease status. A locus on chromosome 3 (rs71306566[C]), in the *AADACL2-AS1* gene region, showed association with Aβ42 in AD and a locus on chromosome 5 (rs75840268[T]), located near *EPB41L4A* in CO. The rs71306566 variant had consistent direction in cases and controls and was significantly different in AD compared to both meta-analysis (*p* = 8.32 × 10^-05^) and CO groups (*p* = 0.002; Supplementary Data 5). This locus did not have any significant effect on AD risk (*p* = 0.318), progression (*p* = 0.789) or brain amyloidosis (*p *= 0.107). Similarly, rs75840268 had significantly different effect size between CO and AD (*p *= 6.44 × 10^−06^) and with the meta-analysis (*p* = 2.8 × 10^−04^) with an opposite direction of effect between AD and CO (Supplementary Figs. 13, 14). This variant showed a nominal association with AD risk (*p *= 0.027) and brain amyloidosis (*p* = 0.010). We did not observe any significant colocalization of this locus with CSF pQTLs or brain eQTLs and therefore could not implicate a functional gene. The rs192462264[A] variant on chromosome 7 was close to genome wide significance in AD but failed to pass the threshold (*p* = 1.178 × 10^-07^; Supplementary Fig. 13).

In the SNP-Diagnosis interaction analysis, no variants reached genome-wide significance threshold (Supplementary Fig. 15). This finding could be potentially due to limited statistical power to detect minor difference in effect between the diagnosis group or could be because the variants identified in our meta-analysis modulate biomarker levels consistently across both diagnosis group.

### Comparison between endophenotypes versus disease status as phenotype of interest

Because endophenotypes are thought to have a higher effect size compared to disease status, we also performed sensitivity analysis by running identical GWAS model in same set of samples (*N* = 5128 [AD = 2365; CO = 2763]) to ensure that the statistics are free of methodological differences. Because our biomarkers were z-scored, we calculated t-statistics for each regression coefficient to make them directly comparable. Overall, the effect size across endophenotype and diagnosis appears to be highly correlated (Supplementary Fig. 16). However, we also observed that for variants associated with genes that are known to directly influence the endophenotypes, these association showed up to 20-fold increase in magnitude of effect when compared to disease GWAS (Supplementary Data 6). For example, variant in *APOE* gene which is a direct regulator of Aβ42 levels had a z-score of −29.587 compared to 15.589 in diagnosis-based analysis with a p-value in order of 10^−177^ compared to 10^−54^. Similarly, the *MS4A6A* variant had 1.5 times higher effect size in t-tau and p-tau181 compared to diagnosis (Supplementary Data 6). *MAP1LC3B* variant which is associated with t-tau and p-tau181 had almost 20 times higher effect on these endophenotypes compared to diagnosis status. So, although effect sizes were highly correlated and comparable between endophenotypes and diagnosis status, we found that depending on involvement of variants in mechanism relevant to the endophenotypes, these effects are significantly increased. This highlights the potential of endophenotype-based study to capture the biological underpinnings of complex disease more robustly, which would otherwise be diluted when focusing on diagnosis status only.

### Gene prioritization for significant loci

Using a previously validated 50-point weighted presence-absence scoring matrix^4^, we performed a functional gene nomination for our significant loci. This approach utilizes evidence from variant annotation, colocalization, QTL overlap, and gene-based analysis as described in the methods section (Supplementary Data 7). All genes within 1 Mb up and downstream of the lead variant were evaluated (Supplementary Data 8).

We were able to nominate a functional gene for nine significant loci (Table 3). *CR1* was nominated as the candidate gene at the chromosome 1 locus associated with CSF Aβ42 based on evidence from colocalization as well as annotation and gene-based analysis (Table 3, Supplementary Data 8, 9, 10). The locus colocalized with eQTL for *CR1* in the caudate nucleus (PP.H4 = 0.986), cerebral cortex (PP.H4 = 0.982), putamen (PP.H4 = 0.977), nucleus accumbens (PP.H4 = 0.953) and frontal cortex (PP.H4 = 0.894; Supplementary Data 9). Another gene within the complement cascade, *CR2*, also passed our gene nomination threshold (≥4) for prioritization but was not nominated as functional because it scored lower overall than *CR1* (Supplementary Data 8). The chromosome 2 locus, associated with all three CSF biomarkers, is the well-known *BIN1* AD risk locus, and consistently *BIN1* was nominated by the prioritization approach with no other gene in the locus scoring the required minimum (Supplementary Data 8). *CCDC50*, which is in the same 3q28 chromosomal region as *OSTN* and *GMNC*, was nominated as the functional gene for the lead variant in chromosome 3 based on the evidence of colocalization between the t-tau and p-tau181 associations and CSF pQTL for CCDC50 (Table 3, Supplementary Data 9). Within chromosome 7, we had two signals (one for t-tau and the other for p-tau181; rs798490[A] and rs798560[G], respectively, *r*^2^ = 0.810). *GNA12* was prioritized as the candidate gene for both based on the presence of a protein altering variant (rs798488 [p.Met1Val]; *r*^2^ = 0.957, Supplementary Data 11, 12) and the gene-based association test (MAGMA *p* = 7.46 × 10^−10^; Table 3). In this same region *AMZ1* scored second in the prioritization algorithm. The t-tau and p-tau181 signal colocalized (PP.H4 > 0.8) with *AMZ1* eQTLs in anterior cingulate cortex, cerebellar hemisphere, cortex, hypothalamus, cerebellum and nucleus accumbens (Supplementary Data 9).

**Table 3:** Table showing the highest scoring genes and their respective scores in loci identified by meta-analysis

MarkerName	Gene Name	Total	Protein Altering Variant	MAGMA	Closest Gene	pQTL-Coloc	GTEx-Coloc	MB-coloc	pQTL-Sig Locus	GTEx-sig Locus	MB-sig Locus	pQTL-Lead	GTEx-Lead	MB-Lead
chr1:207625371:T:G	*CR1*	48	19	0	1	7	4	4	2	1.5	1.5	3	2.5	2.5
	*CR2*	13.5	0	0	0	7	0	0	2	1.5	0	3	0	0
chr2:127135234:C:T	*BIN1*	4	0	0	1	0	0	0	0	1.5	1.5	0	0	0
chr3:190953006:C:T	*CCDC50*	7	0	0	0	7	0	0	0	0	0	0	0	0
chr7:2761908:G:A	*GNA12*	23.5	19	2	1	0	0	0	0	1.5	0	0	0	0
	*AMZ1*	18	0	2	0	0	4	4	0	1.5	1.5	0	2.5	2.5
chr7:2718675:A:G	*GNA12*	20.5	19	0	0	0	0	0	0	1.5	0	0	0	0
	*AMZ1*	19	0	2	1	0	4	4	0	1.5	1.5	0	2.5	2.5
chr8:107381731:A:G	*ANGPT1*	2.5	0	0	1	0	0	0	0	0	1.5	0	0	0
chr9:2197987:A:G	*CARM1P1*	1.5	0	0	0	0	0	0	0	1.5	0	0	0	0
	*KCNV2*	1.5	0	0	0	0	0	0	0	0	1.5	0	0	0
	*VLDLR*	1.5	0	0	0	0	0	0	0	1.5	0	0	0	0
chr11:60254475:G:A	*MS4A6A*	21	19	2	0	0	0	0	0	0	0	0	0	0
chr11:60207692:G:A	*MS4A6A*	21	19	2	0	0	0	0	0	0	0	0	0	0
chr12:21279895:C:T	*SLCO1A2*	19	0	2	1	0	4	4	0	1.5	1.5	0	2.5	2.5
	*RECQL*	4	0	0	0	0	4	0	0	0	0	0	0	0
chr16:87199910:G:A	*MAP1LC3B*	10	0	0	0	7	0	0	0	1.5	1.5	0	0	0
	*C16ORF95*	3.5	0	2	0	0	0	0	0	0	1.5	0	0	0

*MB* MetaBrain, *MAGMA* Multi-marker Analysis of GenoMic Annotation, *pQTL* protein Quantitative trait loci.

Genes highlighted in bold are those that were nominated as likely functional in the loci. Details of weighted scoring criteria is presented in Supplementary Data 7.

The lead variant on chromosome 11 was shown to be in the *MS4A* gene region (Supplementary Figs. 3, 6). We identified two different lead variants for t-tau and p-tau181, but they were in LD (rs7928895[A], rs1582763[A], *r*^2^ = 0.795, respectively). Rs1582763 was previously reported to be associated with sTREM2 and mapped to the *MS4A4A* gene^13^. However, we nominated *MS4A6A* as the candidate gene for both these variants (Table 3, Supplementary Data 8), which was driven by the presence of protein altering variants (rs7232 [p.Thr185Ser]; Supplementary Data 11, 12) and having passed the significance threshold in the gene-based analysis (MAGMA *p* = 1.53 × 10^−07^).

**Fig. 3:**
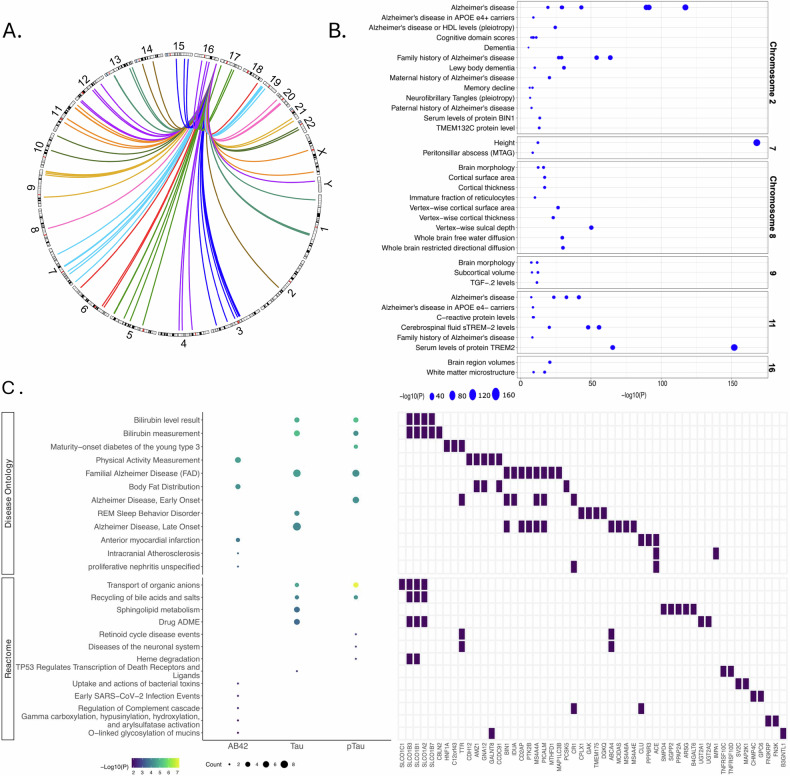
Downstream analysis of prioritized genes. **A** Circos plot showing trans pQTL that colocalized with our chromosome 16 GWAS loci. **B** Previously reported trait associated with our lead variants from GWAS catalog. *p*-values correspond to unadjusted *p* from the underlying GWAS catalog summary statistics. **C** Reactome pathway and disease ontology analysis of genes associated with variants that passed suggestive significance threshold in each of our the phenotype. Significance threshold was unadjusted *p* < 0.05. Y-axis represents top 5 pathways/terms associated with each phenotype. The tile plot shows genes involved in these pathways. Source data for this figure are shown in Supplementary Data 13, 17 and 18.

We nominated *SLCO1A2* as the candidate gene for the chromosome 12 locus based on colocalization with eQTLs in the cerebellum based on GTEx v8 Brain eQTL and MetaBrain eQTL (Table 3, Supplementary Data 9). *MAP1LC3B* was nominated as the candidate gene at the chromosome 16 locus because it colocalized with a CSF pQTL (Table 3, Supplementary Data 9). In addition, it also had a significant QTL in both GTEx Brain eQTL and MetaBrain eQTL (Table 3). *C16orf95*, which was previously reported to be a functional gene in this locus, was the second highest scoring gene but failed to pass the nomination threshold of 4 (Supplementary Data 8)^5^. It scored a total of 3.5 based on significance in gene-based analysis (MAGMA *p* = 5.53 × 10^−16^) and presence of significant GTEx brain eQTL associated with the gene (Supplementary Data 8). We observed that the *MAP1LC3B* locus colocalized with trans-pQTL signals for 80 unique genes including *NECTIN*, *NLGN1*, *CNTFR*, *ATP1B2* (Fig. 3A, Supplementary Data 13).

We were unable to prioritize candidate genes for the loci on chromosomes 8 and 9 because none of the tested genes passed the scoring threshold. The closest genes to the lead variants in these loci were *ANGPT1* and *SMARCA2* based on FUMA analysis. *ANGPT1* was in fact the highest scoring gene on the chromosome 8 locus even though it scored <4 in the weighted scoring approach (Table 3).

### Significant genetic loci show association with AD risk and progression

We aimed to determine whether the lead genetic variants associated with CSF AD endophenotypes have also been implicated in other AD-related phenotypes. We queried publicly available resources to identify associations with AD risk, brain amyloidosis, and AD progression^4,17,18^. Variants near *CR1* associated with Aβ42 were also significantly associated with increased AD risk (OR = 1.127, confidence interval=1.104-1.150, *p* = 4.69 × 10^−31^; Table 2) and brain amyloidosis (Effect size = 0.097, SE = 0.016, *p* = 4.75 × 10^−31^; Table 2). Similarly, the lead GWAS variant on chromosome 2 near *BIN1* was also associated with increased AD risk (OR = 1.184, confidence interval=1.164-1.203, *p* = 6.48 × 10^−90^; Table 2) as well as accelerated AD progression (Effect size=1.18×10^−04^; SE = 4.07 × 10^−05^, *p *= 3.89 × 10^−03^, Table 2). Lead variants in the *GNA12* locus associated with t-tau and p-tau181 were also significant for increased AD risk (OR = 1.020, confidence interval=1.002-1.038, *p* = 2.51 × 10^−02^ for t-tau variant; OR = 1.029; confidence interval=1.011-1.047, *p* = 1.24 × 10^−03^ for p-tau181 variant; Table 2). However, they did not appear to effect disease progression or brain amyloidosis significantly (Table 2). Two distinct variants were associated with t-tau and p-tau181 on chromosome 11 within the *MS4A* gene region (LD *r*^2^ = 0.795). A recent study reported variants within *MS4A4A* (rs10897026) and *MS4A6A* (rs72918674) that modulate sTREM2 levels^23^. The lead variants from our meta-analysis were in high LD with the reported functional variants in *MS4A6A* (*r*^2^_t-tau vs MS4A6A_ = 0.931; *r*^2^_p-tau181 vs MS4A6A_ = 0.755) but not with the *MS4A4A* variant (*r*^2^_t-tau vs MS4A4A_ = 0.068; r^2^_p-tau181 vs MS4A4A_ = 0.145). In line with their associations with lower CSF t-tau and p-tau181 levels, both alleles from the analysis were also significantly associated with reduced risk of AD (t-tau variant OR = 0.928, confidence interval=0.913-0.943, *p* = 2.08×10^-19^; p-tau181 variant OR = 0.918, confidence interval=0.903-0.933, *p* = 1.65×10^-24^; Table 2), with 96% and 99% probability of being the causal variants within the locus for AD risk as well, based on colocalization evidence (Supplementary Data 14). However, only the allele associated with reduced t-tau levels was found to be significantly associated with slowing disease progression (Effect size = -8.97×10^-05^; SE = 4.24×10^-05^, *p* = 3.45×10^-02^; Table 2). Our chromosome 16 lead variant in the *MAP1LC3B* region that was found to be significantly associated with both t-tau and p-tau181 in the meta-analysis was also significantly associated with p-tau181 in previously published CSF GWAS study (Z = 6.61, *p* = 3.85×10^-11^; Supplementary Data 1)^5^. However, the allele associated with increased CSF t-tau and p-tau181 allele appears to decrease the risk of AD (OR = 0.982, confidence interval=0.966-0.998, *p* = 3.21×10^-02^). Chromosome 9 loci, a known schizophrenia risk loci near *SMARCA2*, did not show significant association with AD risk or progression but was associated with brain amyloidosis (Effect size=0.033; SE = 0.014, *p* = 2.25×10^-02^; Table 2)^24^. Lead variants on *SLCO1A2* and chromosomes 8 loci did not show significant associations with any other phenotypes evaluated. As mentioned in earlier sections, *ANAX4/ GMCL1* and *CCNO* loci, identified in pleiotropy adjusted analysis, were nominally associated with brain amyloidosis (p_chr2_ = 0.023; 0.016; p_chr5_ = 0.022). Chromosome 5 loci associated with t-tau in CO, identified in diagnosis stratified analysis, showed nominal association with AD risk (*p* = 0.027) and brain amyloidosis (*p* = 0.010). In sum, many of the lead variants showed converging evidence across CSF biomarkers, AD risk and progression.

### Shared genetic architecture with other traits of interest

**Fig. 4:**
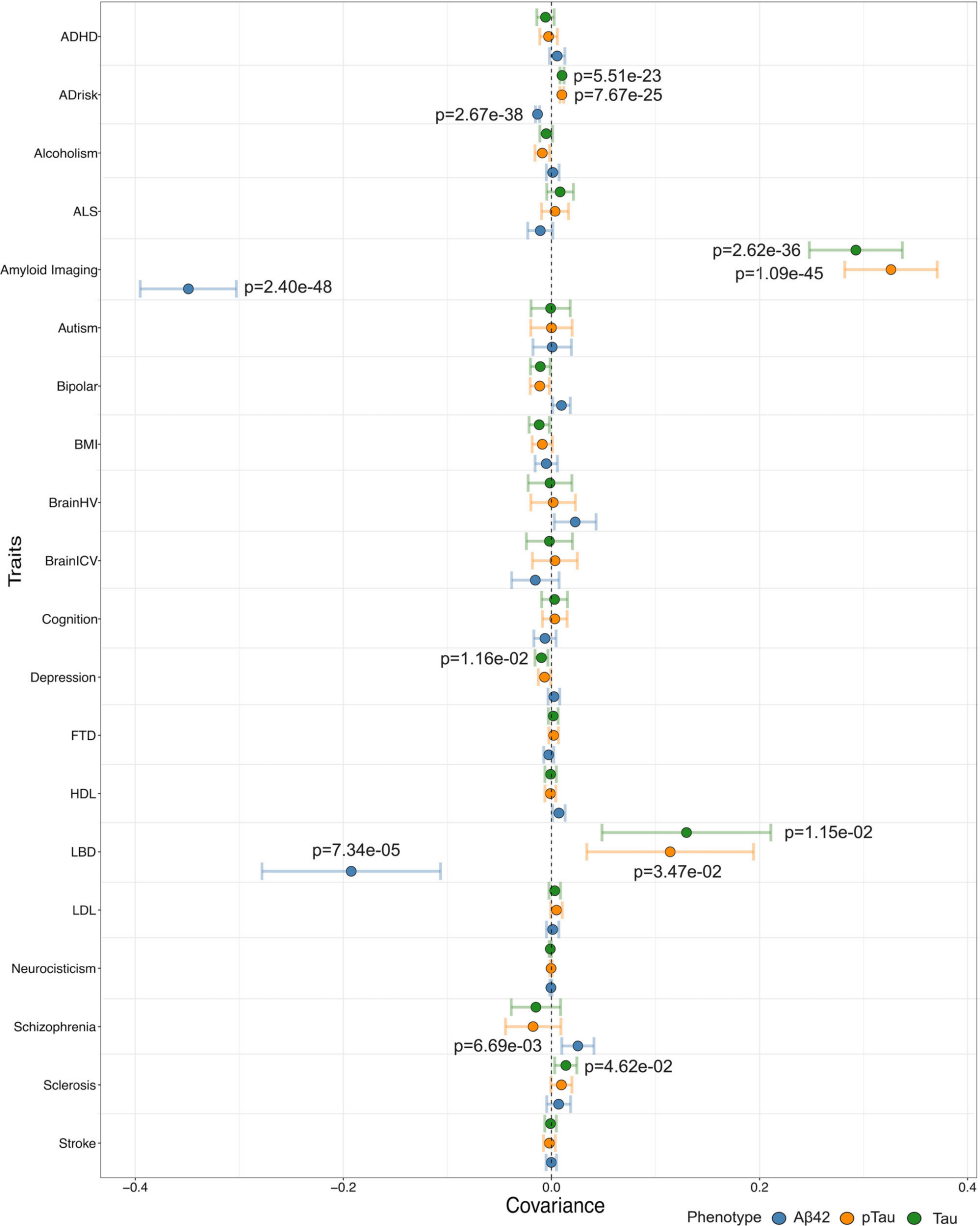
Genetic covariance with other traits. Dot and whisker plot showing the covariance coefficient for each phenotype and across 20 different traits: Attention deficit hyperactivity disorder (ADHD; n = 225,534), Alzheimer’s Disease risk (n = 788,989), Alcoholism (n = 3,383,199), Amyotrophic lateral sclerosis (ALS, n = 152,268), Amyloid Imaging (N = 11,556), Autism (n = 46,351), Bipolar disorder (n = 413,466), Basal Metabolic Index (BMI, n = 484,680), Brain hippocampal volume (n = 26,814), Brain intra-cranial volume (n = 26,577), Cognitive Performance (n = 257,828), Depression (n = 1,035,760), Frontotemporal Dementia (FTD, n = 19,993), High Density Lipoprotein (HDL, n = 99,900), Lewy Body Dementia (LBD, n = 7372), Low Density Lipoprotein (LDL, n = 95,454), Neurocisticism (n = 449,484), Schizophrenia (n = 320,404), Sclerosis (n = 115,803), Stroke (n = 521,612). Data is presented as point estimate of gentic covariance coefficients and error bars show 95% confidence interval (CI) around those estinates. Color of the dots represent each phenotype. False discovery rate (FDR) adjusted *p*-value is shown for that covariance that passed significance threshold (FDR P < 0.05). Source data for this figure is shown in Supplementary Data 15.

The interplay among similar genetic factors often contributes to multiple phenotypic effects^25^. Thus, by examining the relationships between complex polygenic traits we can better understand their genetic underpinnings and identify shared biological pathways. We evaluated the genetic covariance of the three CSF endophenotypes with AD risk and 19 other human-health related traits using GNOVA (Fig. 4, Supplementary Data 15). As additional check, these estimates were also confirmed using Linkage Disequilibrium Score Regression (LDSC; Supplementary Fig. 17).

We found significantly negative genetic covariance of CSF Aβ42 with AD risk (r_g=_−0.013, SE = 0.001, p_FDR_ = 2.67×10^-38^) and brain amyloidosis (r_g=_−0.349, SE = 0.024, p_FDR_ = 2.40×10^-48^). Similarly, we observed significant positive genetic covariance of CSF t-tau and p-tau181 with AD risk (r_g-t-tau=_0.01, SE = 0.001, p_FDR_ = 5.51×10^−23^; r_g-p-tau181=_0.01, SE = 0.001, p_FDR_ = 7.67×10^-25^) and brain amyloidosis (r_g-t-tau_ = 0.293, SE = 0.023, p_FDR_ = 2.62×10^-36^; r_g-ptau181_ = 0.326, SE = 0.023, p_FDR_ = 1.09×10^−45^). These findings are consistent with established directions of effect on AD risk for these biomarkers^9^.

We also observed genetic covariance between these CSF endophenotypes and other brain disorders, with all three of the endophenotypes showing significant covariance with Lewy Body Dementia (LBD; r_g-Aβ42_ = −0.192, SE = 0.044, p_FDR_ = 7.34×10^−05^; r_g-t-tau=_ 0.130, SE = 0.041, p_FDR_ = 1.15×10^−02^; r_g-p-tau181_ = 0.114, SE = 0.041, p_FDR_ = 3.47×10^−02^). Schizophrenia showed significant covariance with Aβ42 (r_g_=0.025, SE = 0.008, p_FDR_ = 6.69×10^−03^). Similarly, depression and multiple sclerosis were found to have significant covariance with t-tau (r_g-depression_ = -0.01, SE = 0.003, p_FDR-depression_ = 1.16×10^−02^; r_g-sclerosis-_=0.014, SE = 0.005, p_FDR-sclerosis_ = 4.62×10^-02^) but not Aβ42 or p-tau181. Circulating HDL levels are reported to be associated with decreased AD risk, and we observed nominally significant and positive covariance with CSF Aβ42 (r_g-_=0.007, SE = 0.003, *p* = 1.96×10^−02^) in our analysis, although this failed to pass the FDR-corrected significance threshold. Hippocampal volume was nominally associated with Aβ42 (r_g-_=0.023, SE = 0.010, *p* = 2.58×10^-02^; p_FDR_ = 0.077) but not with t-tau and p-tau181. Exclusion of the *APOE* region had no effect on the pair-wise genetic covariance (Supplementary Data 15). We observed significant and strong correlation between estimates from GNOVA and LDSC with coefficients being 0.975 (*p* = 2.9×10^−13^), 0.85 (*p* = 2.05×10^−06^), and 0.816 (*p* = 1.14×10^−05^) for Aβ42, t-tau and p-tau181 respectively (Supplementary Fig. 17). When these comparisons were repeated without including the APOE gene region, similar strong and significant correlation were observed (r_Aβ42_ = 0.86, p_Aβ42_ = 1.04×10^-06^; r_t-tau_ = 0.741, p_t-tau_ = 1.83×10^−04^; r_p-tau181_ = 0.701, p_p-tau181_ = 5.77×10^-04^). In summary, our findings indicate that common genetic factors may influence both the CSF biomarkers and other neurological disorders, with AD-related traits showing stronger associations.

### CSF Aβ42 is causally associated with increased risk of AD

Many of our lead variants from meta-analysis were also significantly associated with AD risk, so we sought to investigate causal link between the three biomarkers and disease risk.

Mendelian randomization (MR) analyses were performed using the biomarkers as exposure and AD risk^4^ as outcome variable. MR was performed using five methods; MR Egger, Weighted Median, Inverse Variance Weighted (IVW), Simple Mode, and Weighted Mode. All five methods identified a significant negative association between Aβ42 and AD risk indicating that higher levels of Aβ42 are causally associated with a reduced risk of Alzheimer’s disease (Supplementary Data 16). Specifically, the IVW method demonstrated an effect size of -1.806 and a highly significant p-value (*p* = 1.14×10^−16^). On the other hand, t-tau and p-tau181 did not have any significant association with AD risk in any of the methods tested. Additionally, pleiotropy check done as part of the MR pipeline did not show any significant pleiotropy between the biomarkers and AD risk (p_Aβ42_ = 0.379; p_Tau_ = 0.380; p_p-tau181_ = 0.520) showing that these results are not biased due to genetic pleiotropy. Overall, our results from MR indicate that while CSF Aβ42 is directly linked to AD risk, CSF t-tau and p-tau181 do not directly influence risk of AD disease.

### PheWAS analyses

We then queried the GWAS catalog for our lead variants (excluding chromosome 19) to identify other associated phenotypes; and identified 63 trait associations that were reported for six of them (Fig. 3B, Supplementary Data 17). The majority of the associations were for the variant in the *BIN1* locus (N = 26). These included AD, dementia, cognition or similar traits (Supplementary Data 17). For the variant in the *MS4A* gene region, we found associations with 10 traits that all pointed to involvement in sTREM2 levels and AD. Lead variants on chromosomes 8, 9 and 16 were predominantly reported to be related to brain matter, with each related to a different brain region (N = 10, 5, and 3, respectively; Supplementary Data 17). Variants on chromosome 8 seem to be associated with cortical region thickness and surface area whereas variants on chromosome 9 were associated with subcortical volume. Similarly, the *MAP1LC3B* variant was found to be associated with white matter volume. At the time of the search, neither the *CR1* variant associated with Aβ42 nor the *SLCO1A2* variant associated with t-tau and p-tau181 had any reported trait associations. However, this may be a result of limitations in current catalog coverage.

### Pathway analysis implicates lipid metabolism dysregulation independent of *APOE*

To better understand the biological mechanisms behind the combined effects of significant GWAS loci on the endophenotypes, we performed pathway analysis using the genes linked to independent variants in suggestively significant loci (<10^-05^) by FUMA. Genes in the *APOE* locus were not included as they tend to bias results towards lipid metabolism. Genes associated with Aβ42 were involved in immune system pathways (regulation of complement cascade, *p* = 8.53×10^-03^), post-translational protein modification (Gamma carboxylation, hypusinylation, hydroxylation, and arylsulfatase activation, *p* = 1.04×10^-02^; O-linked glycosylation, *p* = 1.54 × 10^-02^) and infection pathways (Uptake and actions of bacterial toxins, *p* = 3.78×10^-03^; Early SARS-CoV-2 Infection Events, *p* = 5.64 × 10^-03^; Bacterial Infection Pathways, *p* = 1.98 × 10^-02^; Fig. 3C, Supplementary Data 18). *CR1* and *CLU* genes were the key driver of immune-related pathways in our analysis. *FN3KRP, FN3K, GALNT8, B3GNTL1, PPP6R3* and *DYNLL1* were some of the major genes involved in post translational modification pathways. Of these genes, *DYNLL1* has been previously reported to be dysregulated in AD brain^26^. Proper post-translational modification is necessary for protein homeostasis and dysregulation of these processes may result in the accumulation of amyloid plaques and t-tau tangles.

We observed that genes associated with t-tau levels showed enrichment for lipid metabolism pathways even though genes within the *APOE* locus were excluded from this analysis (Sphingolipid metabolism, *p* = 3.1×10^-04^; Recycling of bile acids and salts, *p* = 1.16×10^-04^; Sphingolipid catabolism, *p* = 1.53×10^-03^; Bile acid and bile salt metabolism, *p* = 1.82×10^-03^, Fig. 3C, Supplementary Data 18). *SLCO1A2, SLCO1B3, SLCO1B1, B4GALT6, SMPD4, SGPP2, PPAP2A* and *ARSG* were involved in these pathways. Other pathways that showed enrichment in genes associated with t-tau included those linked to cellular transport (Transport of organic anions, *p* = 2.41×10^-05^; Transport of vitamins, nucleosides, and related molecules, *p* = 1.49×10^-03^), disease (Diseases of the neuronal system, *p* = 2.89×10^-03^; Retinoid cycle disease events, *p* = 2.51×10^-03^; Diseases associated with visual transduction, *p* = 2.51×10^-03^) and drug kinetics (Drug ADME, *p* = 1.49 ×10^-03^; Fig. 3C).

Pathways enriched in p-tau181-associated genes were highly correlated with those observed in t-tau with varying degrees of significance (Fig. 3C). The most enriched disease ontology terms highlighted associations with liver function markers (Bilirubin level result, p_tau_ = 1.50×10^-05^, p_ptau181_ = 6.32×10^-06^; Bilirubin measurement, p_tau_ = 6.61×10^-06^, p_ptau181_ = 6.02×10^-05,^ Supplementary Data 18). Familial AD was also one of the top enriched disease terms in the analysis (p_tau_=3.20×10^-05^, p_ptau181_ = 5.83×10^-05^). Disease ontology terms enriched for genes associated with Aβ42 highlighted lifestyle factors (Physical Activity Measurement, *p* = 3.02×10^-05^; Body Fat Distribution, P = 5.11×10^-05^; Fig. 3C). REM Sleep Behavior Disorder, a significant lifestyle factor associated with neurodegeneration^27^, was also significantly enriched (p_tau_=6.48×10^-05^; p_ptau181_ = 7.05×10^-04^; Fig. 3C, Supplementary Data 18).

Our *MAP1LC3B* locus colocalized with trans-pQTL signals for 80 unique genes in multiple chromosomes (Supplementary Data 13). Pathway analysis of all genes that had a QTL colocalize with this locus showed enrichment of neuronal development pathways (synapse assembly, *p* = 9.45×10^-09^; axon development, *p* = 3.60×10^-^^07^; neuron projection morphogenesis, *p* = 5.49×10^-07^; cell morphogenesis involved in neuron differentiation, *p* = 6.89×10^-07^; Supplementary Fig. 18, Supplementary Data 19). This suggests that chromosome 16 locus may be contributing to the disease process through changes in neural development. Overall, we identified critical roles of immune system regulation, protein modification, lipid metabolism, and neural development pathways in AD pathophysiology.

## Discussion

Previous GWAS for CSF Aβ42, t-tau, and p-tau181 as AD endophenotypes have identified loci associated with the disease and highlighted potential disease-relevant mechanisms^5,11,12,28^. In this study, we performed a large meta-analysis using data from a total of 18,948 subjects, making this study the largest to date for these endophenotypes. We identified 12 genome-wide significant variants across all three biomarkers, eight of which were novel in their respective association. Compared to diagnosis-based GWAS, we found that endophenotype based study captures the biology of the disease more robustly. Majority of these loci were also observed in cross-trait comparison accounting for pleiotropy between the biomarkers. These variants were not only associated with CSF level of the three biomarkers but also showed significant association with AD risk, disease progression and brain amyloidosis (Table 2). Some of these associations were only nominally significant, however, it is important to note that we had predefined hypothesis on the expected effect of these variants. We also observed significant genetic covariance with AD risk, brain amyloidosis and Lewy body dementia for the three biomarkers. In fact, evidence from MR analysis showed that CSF Aβ42 is associated with risk of AD.

Consistent with what has been reported previously, *APOE* locus was the most significant association in all three biomarkers^5,11,12^. Association of *APOE* with Aβ42 was the only association identified in samples from both European and non-European ancestry. Three independent signals in the *APOE* gene: ɛ4 and ɛ2 and an additional variant, rs5117 mapped to *APOC1* gene, were found to be associated with the three CSF biomarkers The rs5117 variant was reported to be associated with brain amyloidosis previously^17^. We identified *APOE* to be associated with all three biomarkers in our meta-analysis, but cross- trait analysis showed it to be Aβ42 specific. However, previous research has shown that although part of *APOE*-t-tau/ptau181 interaction is mediated through Aβ42, some variants within the region affect tau/ptau-181 independently^12^. In addition, we also confirmed previously reported associations near *CR1* with Aβ42 along with identifying novel association near *BIN1* with Aβ42. *BIN1* is one of the most important risk locus for AD^4,8^ and we observed strong colocalization of our *BIN1* locus with previously reported *BIN1* locus associated with AD risk (PP.H4 = 1; Supplementary Data 14)^4^. Further, the variant associated with CSF Aβ42 was also significantly associated with disease progression and brain amyloidosis (Table 2). Similarly, *CR1* locus colocalized with pQTL and eQTL signals from CSF and brain respectively. This evidence shows that *CR1* contributes to the disease process through both its transcriptional and translational regulation. In addition to the *CR1* gene, we propose *CR2* as an additional functional gene in this region as it was the second-highest scoring gene and passed our nomination threshold. We have previously reported an association between CR2 protein levels and AD risk^29^. In addition, *CR2*, like *CR1*, is a member of the complement system and has a role in inflammation and immune responses, both of which are associated with AD pathology^30^. Because of the similarities between the two genes, they may be acting together to influence CSF Aβ42, and by extension, AD. Not surprisingly, Pathway analysis of loci associated Aβ42 levels highlighted dysregulation of immune system pathways mostly driven by the *CR1* and *CLU* genes.

Previously reported associations on chromosomes 3 and 16 with both t-tau and p-tau181 were also confirmed^5,11^. However, we propose *CCDC50* and *MAP1LC3B* as functional at these loci respectively. *CCDC50* has been reported to be highly expressed in the brain and plays a role in protein clearance^31,32^. Ye et al.^31^ demonstrated aggregation of endogenous *CCDC50* in human cell lines with mutant MAPT/t-tau species^31^. Their study also demonstrated that *CCDC50* readily binds to and isolates these “aggregation-prone” t-tau species promoting their degredation^31^. These observations provide support that *CCDC50* plays an integral role in the autophagic degradation of t-tau protein aggregation, which is the hallmark of AD and other neurodegenerative diseases. Similarly, *MAP1LC3B* is a major marker of autophagy^33^. Research has shown that astrocytic knockdown of *MAP1LC3B* in APP/PS1 mouse models worsened cognitive impairments while increasing t-tau phosphorylation at serine 202 and threonine 205^34^. Both loci have previously been reported to be associated with ventricular volume^5,35,36^. In addition to *CCDC50*’s role in the tau pathology, Ye et al.^31^ also demonstrated that *ccdc50* knockout in mouse models led to development of hydrocephalus^31^. Further, PheWAS analysis of the lead variant in *MAP1LC3B* locus identified its associations with brain volume and white matter microstructure (Fig. 3B). In the same context, *GNA12* and *SLCO1A2* genes, which were nominated as functional for the novel chromosome 7 and 12 association respectively with both t-tau and p-tau181, have also recently been linked to be Normal pressure hydrocephalus (NPH), a condition marked by abnormal CSF buildup in brain ventricles^37^. *GNA12* has been reported to decelerate hyperphosphorylation of t-tau where as *SLCO1A2* is a known risk gene associated with progressive supranuclear palsy^38,39^. The *GNA12*, *MS4A6A* and *MAP1LC3B* loci were also found to be shared with Aβ42 via cross-trait analysis, likely pointing to an interaction between the biomarkers at these loci. Although we were unable to nominate functional gene for chromosome 8 and 9 loci, associated with t-tau and both t-tau and p-tau181 respectively, PheWAS analysis of the lead variants in these regions revealed associations with brain morphology, cortical volume and surface area, and brain water diffusion. GNOVA analysis investigating shared genetic architecture of CSF t-tau and ptau181 levels with hippocampal volume and intracranial volume was not significant. However, this finding likely reflects lack of polygenic signal across the traits without capturing the influence of the individual variants identified in our study. Overall, our findings suggest structural alteration in brain volume may be a key mechanism associated with tau mediated AD pathology.

Besides their association with intracranial volume, the *CCDC50* and *MAP1LC3B* loci shared some additional shared characteristics. The *CCDC50* locus is reported to be pleiotropic in nature^29^ and our findings suggest that the *MAP1LC3B* locus shows similar pleiotropic characteristics. It was shown to affect regulation of 80 distinct proteins across all 23 chromosomes (Fig. 3A). Majority of the genes that encoded these regulated proteins were neuronal and involved in pathways associated with synapse assembly (*FLRT3, NLGN1, SEMA4A, ADGRL3, NECTIN1, LRRC4, LRFN4*) and neuron projection (*EFNA2, EFNB2, UNC5B, NLGN1, SEMA6B, SYT1, OLFM1, FSTL4*). Further, our protein-protein interaction analysis showed that the protein product of the *CCDC50* gene was found to interact with the protein product of *MAP1LC3B* (Supplementary Fig. 19). In light of these findings, we hypothesize that these two genes play a distinct but complementary role in the regulation of t-tau and p-tau181 levels. In addition to the alteration in brain volume, disruption of the blood-brain barrier (BBB) could be another potential mechanism contributing to tau mediated disease process. *ANGPT1*, the closest gene to the chromosome 8 locus lead variant, is known to play a role in BBB integrity maintenance and *SMARCA2* near our chromosome 9 lead variant is involved in dendritic growth and morphogenesis^24,40,41^. *SLCO1A2*, functional gene at the chromosome 12 locus, encodes a sodium-independent membrane transporter and is highly expressed in human brain endothelial cells that are responsible for selective permeability of the BBB^42–44^. *SLCO1A2* has been previously reported to be associated with cortical Aβ deposition in AD^45,46^. However, its role in tau aggregation is not well understood. Induced pluripotent stem cell (iPSCs) models from familial AD showed significant downregulation of *SLCO1A2*^46^. Given the primary role of *SLCO1A2* is passive transport to the brain across the concentration gradient, we suspect that it contributes to disease process through decreased availability of substrates required for clearing tau tangle toxin in brain^46,47^. We observed that *SLCO1A2* signal from GWAS colocalized with eQTL signal from both GTEx and Metabrain in cerebellum. Cerebellum is usually though to be spared in early AD disease, with involvement being reported in later disease stage^48^. This suggests *SLCO1A2* mediated regulatory changes may occur in later disease stage in specific brain regions. In addition to all these, other potential disease mechanisms, highlighted by cross-trait analysis, included aggregation of tau in axons and disturbances in the AKT signaling cascade mediated by *ANAX4* and *CCNO* gene^20,22^.Disease stratified analysis identified a chromosome 5 signal specific to controls that was nominally associated with both AD risk and brain amyloidosis. But due to lack of any colocalization evidence, we were unable to identify functional gene in the locus. However, because this analysis was limited to samples with raw data, future studies with larger sample size may be able to better characterize this locus.

Our pathway analysis of significant loci associated with t-tau and p-tau181 showed disruption of bile and bile salt related metabolism, driven by *SLCO1A2, SLCO1B3, SLCO1B7*, and *SLCO1B1* genes. Disease ontology analysis also found bilirubin-related disease terms to be the most significant for the two phenotypes. Bile acids (BA) are potent regulators of lipids and there is growing evidence suggesting their pathogenic effect in AD through the brain-gut-microbiota (BGM) axis^49,50^. Others have reported their direct association with CSF t-tau and p-tau181 as well as PET-measured biomarkers in AD^51^. A higher level of CSF tau was associated with higher serum BA levels^51^. Disruption in lipid homeostasis is a well-researched disease mechanism in AD, however majority of the current evidence suggests these to be regulated by *APOE* gene^52^. Given our pathway analysis did not include the *APOE* gene region, we hypothesize that there is an APOE-independent axis of lipid dysregulation contributing to t-tau pathology, potentially implicating bile acid metabolism as a therapeutic target.

Another key association that may relate to lipid dysfunction in tauopathy is the novel association of variant in the *MS4A* gene region for t-tau and p-tau181 identified here. Although, the *MS4A* locus is a gene rich region, we nominated *MS4A6A* as functional at the locus based on the presence of a coding variant in the gene. *MS4A* is a well-replicated AD risk locus and genes in this region, including *MS4A6A*, are involved in sTREM2 level regulation^13^. TREM2, a member of immunoglobulin family, is expressed exclusively in microglia in Brain. Microglia are the defense cells of the brain responsible for clearing amyloid plaques and tau tangles in AD. TREM2 acts as sensor for lipids exposed during neuronal damage and are required for activation of microglial response to the injury^53^.Thus, by modulating the level of TREM2, *MS4A6A* is directly responsible for mediating both protective and aggravating microglial response to neuronal insults by tau and its species. Consistently, the *MS4A6A* variant was associated with both risk of AD and disease progression.

While we were able to identify multiple novel loci associated with the phenotypes, some limitations of the study need to be considered. Despite being the largest study for these endophenotypes, we largely focus on the European ancestry population. Our sensitivity analysis with a limited number of non-European subjects highlighted the difference in direction and strength of the association identified, in line with previous studies suggesting that findings from European ancestry cannot be directly translated to other populations^54^. Thus, future research focused on other ancestries will be needed. In addition, we highlight several biological pathways in which genes associated with the phenotypes are involved. However, without additional functional analysis we cannot comment on the directionality of these changes or how these genes might mediate the effects on the highlighted pathways. Third, our gene prioritization method focused on pQTL and eQTL resources. However, incorporating additional resources like methylation QTLs, splicing QTLs and histone QTLs could reveal additional signals that are not captured by pQTL or mQTL alone. Finally, although we performed cross-trait analysis to identify trait specific signals, additional future research to identify CSF Aβ42-independent genetic signals associated with CSF t-tau /p-tau181 will be needed. This would help us better understand the t-tau/p-tau181 dependent disease mechanism and help guide research on alternatives to anti-amyloid therapies.

In conclusion, this work highlights associations between novel loci and CSF Aβ42, t-tau and p-tau181 in addition to replicating findings from previous studies. The pathway analysis identified *APOE-*independent lipid metabolism dysregulation, immune system, and cellular process disturbances as potential underlying disease mechanisms. While future replication of the novel associations in larger studies will be needed, our study demonstrates the power of studying AD-related endophenotypes to better understand disease mechanisms underlying AD risk and disease progression.

## Methods

### Ethics statement

The Institutional Review Board of Washington University School of Medicine in St. Louis approved the study and research was performed in accordance with the approved protocols. Ethics approval for individual cohorts was obtained from their respective IRBs, with written informed consent provided by participants or their families.

### Study design

For this study, we associated individual-level genetic data with CSF biomarker levels in 18,948 unrelated European ancestry individuals from 30 different studies (Table 1). A total of 6785 participants across 22 studies were combined into a single dataset using a z-score based data standardization (mean 0 and variance 1 within each cohort) and analyzed at Washington University in St. Louis Charles F. and Joanne Knight Alzheimer Disease Research Center (Knight ADRC). The summary statistics from this dataset were then meta-analyzed with summary statistics from eight cohorts encompassing 12,163 subjects. The studies that provided summary statistics are: European Alzheimer’s and Dementia Biobank (EADB, N = 7516), The European Prevention of Alzheimer’s Dementia (EPAD, N = 1370), Ace Alzheimer Center Barcelona (ACE^55^, N = 1302), European Medical Information Framework for Alzheimer’s Disease (EMIF-AD, N = 670), MISSION-AD (N = 462, Eisai), Janssen cohorts (Janssen, *N* = 457) and Alzheimer’s and Families cohort (ALFA+ study^56^, N = 386). Notably, the EADB data is a dataset from 13 different cohorts, with details provided in Supplementary Data 20. Within Janssen cohorts, two different sets of study populations were available, one from a BACEi clinical study and the other from a bapineuzumab clinical study^57,58^. More information on these studies is available in Supplementary Materials. Data preparation and analysis approaches were comparable across all studies; related individuals were removed based on identity-by-descent (IBD) analysis (Supplementary Fig. 20) and only European ancestry samples were kept as identified by genetic PCA (Supplementary Fig. 20). Additional analysis was performed using subjects from populations other than those of European ancestry. In total, we had 416 subjects, including 209 African American, 63 Asian and 144 of mixed ancestry (Supplementary Fig. 20). Detailed demographic information on subjects included in this study is available in Table 1 and Supplementary Data 20 and 21. Self-reported gender information was collected; however, no gender specific analysis was performed. Similarly, diagnosis stratified analyses were performed in AD and CO group using samples that had both raw data and disease status available. In total, 2365 AD cases and 2763 CO were available for the diagnosis stratified analysis.

### CSF measurement and quality control

CSF Aβ42, t-tau and p-tau181 levels were measured using different platforms across different studies (Table 1). Detailed information on sample collection and measurement in each study has been described elsewhere^5,57–66^. CSF biomarker values are prone to technical limitations (e.g., ceiling values) and biological artifacts (e.g., cell lysis), thereby requiring quality control (QC) prior to analysis. To this end, we applied a consistent QC approach within each cohort and for each endophenotype separately. First, we performed log10 transformation of the raw biomarker values to approximate a normal distribution and outlier removal. Outliers were defined as any biomarker level lower than Q1-1.5*IQR and higher than Q3 + 1.5*IQR, where Q1 and Q3 are the first and the third quartile, respectively. Following this, we applied a z-score-based data standardization (mean 0 and variance 1 within each cohort) that allows us to combine data from differing sources as continuous quantitative traits. This approach has been used extensively in previous GWAS studies and has been shown to be a robust alternative to meta-analyzing individual cohort summary statistics^11,17,60,67^. Consistent QC was performed across all contributing sites.

### Genotyping and quality control

Genotyping was performed using multiple arrays across different studies (Supplementary Data 22). Despite these differences, a consistent QC and imputation pipeline was applied across all. Detailed QC approaches within each study are presented in Supplementary Data 22 and have been previously described^5,17^. Briefly, prior to imputation, we removed SNPs and individuals with low call rate were (95% threshold in EADB, MISSION-AD and Janssen cohort; 98% threshold in remaining). Autosomal SNPs that were not in Hardy–Weinberg equilibrium (*p* < 1×10^−6^) were then removed. Following this, imputation was performed using the TOPMed Imputation Server (https://imputation.biodatacatalyst.nhlbi.nih.gov/; GRCh38 Version R2 reference panel) for phasing and imputation of non-genotyped single-nucleotide polymorphisms (SNPs) and only variants with imputation quality (R_sq_ or estimated R2) of 0.3 or greater were retained. For the 22 studies, analyzed at Knight ADRC, that were included in the joint analysis, QC was performed in each study individually and then combined for downstream analysis. Duplicated and related subjects were identified using IBD analysis implemented through PLINK (v2.0)^68^. To filter subjects for cryptic relatedness, Pihat>0.2 was used as threshold (Supplementary Fig. 20). We then performed genetic PCA calculation with 1000 Genomes data as the reference cohort and retained only subjects genetically similar to the European population for downstream analysis (Supplementary Fig. 20). Variants with minor allele frequency (MAF) of 0.05% and above were kept for analysis. When the summary statistics file, from contributing sites, were provided in GRCh37 genomic coordinates, the standalone Linux-based UCSC LiftOver program was used to lift them over to GRCh38 prior to analysis. Genomic inflation was assessed for all contributing summary statistics (Supplementary Data 22). Presence of confounding bias in meta-analysis summary statistics was also evaluated using LDSC command line tool^69^.

### Statistical analysis

#### Single variant analysis

Genome-wide association analysis was performed using PLINK (v2.0)^68^. We utilized an additive linear regression model for each CSF biomarker. The default model included age, sex, 10 genetic PCs and cohort-array combination, where applicable, as covariates to account for common confounding factors in GWA studies. A similar approach was used to conduct GWAS analysis in subjects of non-European ancestry as a sensitivity analysis. Subjects from various non-European ancestries were included in these analyses without stratification by specific ancestries as their sample size is not large enough for multi-ethnic-meta-analyses at this time. Diagnosis stratified analysis was performed using samples that had raw data and disease status available. Diagnosis was based on CDR at the time of sample collection or clinically assigned status. The genome wide significance threshold of *p* < 5 × 10^−08^ was used as the cutoff to define significance.

#### Meta-analysis and conditional analysis

Following the single-variant analysis, we meta-analyzed the summary statistics for each of the endophenotypes. We utilized the inverse variance weighted meta-analyses in METAL to perform meta-analysis^70^ Only those variants that were present across 70% of the studies were kept for downstream analysis. A p-value threshold of <5 × 10^-08^ was used to define genome-wide significance. A locus was considered novel if its association with the biomarker had not been previously reported or if it was not in LD with previously reported locus in the latest CSF GWAS, samples from which are also included in this study^5^. The manhattan, forest and regional associations plots were visualized using R packages “qqman” (v0.1.9), “karyoploteR” (v1.30.0) and “meta” (v7.0), along with the stand-alone software LocusZoom (v1.40) respectively^71–74^. To identify additional independent signals within the genome-wide significant loci, we performed conditional analysis using GCTA-COJO^75^. We used the “—cojo-slct” parameter within the tool to identify independent signals. The combined subject-level data (N = 6785, European ancestry) were used as linkage disequilibrium (LD) reference with default COJO parameters (*p* < 5×10^-08^; LD window 10,000 Kb, *r*^*2*^ < 0.9) for the stepwise independent signals selection^29,36^.

#### Cross- trait analysis and pleiotropy check

To further identify shared and trait-specific signals, we applied Multi-Trait Analysis of GWAS (MTAG) using publicly available MTAG tool^14^. Meta-analysis summary statistics for each trait were used as input. rsIDs were used as SNP identifiers for the analysis. Because, MTAG assumes homogenous effects of genetic variants across trait, which is not the case in our biomarkers, we employed an orthogonal pairwise conjunctional false discovery rate analysis using previously established apprach^15,16^. A signal was considered true positive if it met two sets of criteria: (1) it had to pass genome wide significance in MTAG and (2) pass conjunctional FDR *p* < 0.05 for pleiotropy with either of the remaining two biomarker.

#### Variant annotation and gene-based analysis

Variant annotation and gene-based analysis were performed using ANNOVAR and Multi-marker Analysis of Genomic Annotation (MAGMA, v1.10) tools integrated within FUMA (Functional Mapping and Annotation)^76–78^. For FUMA, variant positions were lifted over from GRCh38 to GRCh37 at the time of this analysis. SNPs in LD with the independent significant SNPs are annotated by ANNOVAR in FUMA. Significance is defined as passing the genome-wide threshold (*p* < 5×10^-08^) and an LD threshold of *r*^2^ > 0.6 was used. For gene-based analysis, MAGMA mapped input SNPs to a total of 18,270 genes; thus, we used *p* < 2.6×10^-6^ (0.05/18720) as the significance threshold.

#### Colocalization

To determine if the lead AD CSF biomarker variants also contributed to signals from other molecular quantitative trait loci (QTL), we applied a Bayesian statistical framework implemented through the R “coloc” package^79,80^. We calculated posterior probabilities under the assumption of a single causal variant (“coloc.abf” function) i.e., the same variant is influencing both phenotypes. All variants within 1 Mb up and downstream from the lead variants and common in both GWAS and QTL summary statistics were used. Coloc requires that users provide three sets of prior probabilities; p1: prior probability that a variant is associated with trait 1, p2: prior probability that variant is associated with trait 2, p12: prior probability that a variant is associated with both traits. Default priors were used for both GWAS and QTL summary statistics (p1 = p2 = 10^-04^; p12 = 10^-05^) as this corresponds to *p* < 5×10^-08^ threshold to define a true association^79^. Colocalization was checked using the largest CSF protein QTL resource currently available as well as the MetaBrain eQTL and GTEx v08 Brain eQTL resources (downloaded from GTEx portal on 06/29/2022)^29,81^. If the two traits of interest had >80% probability (H4 > = 0.8) of having the same causal variant, we considered that there is colocalization of the two traits at this locus.

#### Gene prioritization

For each of the lead variants, we used a weighted presence-absence scoring matrix approach to nominate candidate genes, adapted from the one used by Bellenguez et al.^4^. All genes within 1 Mb upstream and downstream from the lead variant were tested. Briefly, each protein coding gene is assigned a score based on evidence from the following criteria: (A) if the index GWAS locus colocalized with a QTL for a given gene. Colocalization with QTLs was assessed against two different molecular phenotypes (protein and mRNA) from three different sources as described above. (B) If the lead variant from meta-analysis is also a significant QTL in these molecular phenotypes (C) If any variant within 1 Mb up and downstream of the lead variant from the meta-analysis passes the significance threshold (*p* < 5×10^-8^) in the QTL summary statistics, the associated gene is scored. (D) If a gene within the 1 Mb region is found significant based on MAGMA gene-based analysis. (E) If a gene is the nearest protein coding gene to the lead variant. (F) If a gene within the 1MB region has a protein-altering variant that passes suggestive significance threshold (*p* < 10^-05^) and is in LD (r^2^ > 0.6) with the lead variant. Each of these scoring criteria have a different scoring weight, details of which are presented in Supplementary Data 7. The maximum score that can be assigned to a gene is 50, and a minimum score of 4 is required for nomination of a candidate gene. If more than one gene in a region passes this nomination threshold, the gene with the highest overall score is nominated as the candidate gene. We did not repeat this scoring mechanism for the well-established lead variant (rs429358) that encoded the *APOE* ε4 variant on chromosome 19.

#### Comparison with other AD-related endophenotypes

We leveraged three resources, each examining different aspects of AD pathogenesis, to investigate whether identified lead variants associated with Aβ42, t-tau, and p-tau181 in our study had been previously implicated in any other AD-related phenotypes. We obtained the largest AD and related dementia risk GWAS and the largest GWAS of brain amyloidosis utilizing in vivo amyloid positron emission tomographic (PET) imaging data^4,5,17,18^. We also looked at variants associated with disease progression by utilizing summary statistics from an in-house AD progression (defined by rate of cognitive decline) GWAS from 7241 subjects^18^. We queried and compared the p-value for the lead variants across all these resources and compared the direction of effect sizes. Genome-wide significance threshold was used to define significance for the variants in all reference summary statistics except AD risk and progression where *p* < 0.05 was used to highlight significance. We then sought to investigate if the novel lead variants have been reported to be associated with other phenotypes. For this, we conducted a phenome-wide association (PheWAS) study using the “gwasrapidd (v0.99)” R package^82^. The “get_studies()” function within the package was used to get a studies metadata, associated with our lead variants of interest from the GWAS catalog^83^ database. Additional metadata information of interest was identified through manual query of the database. The “get_associations()” function within the package was used to extract reported p-values.

#### Mendelian randomization

We used “TwoSampleMR” package in R to perform Mendelian Randomization (MR) between Aβ42, pTau, tau, and AD risk^4,84^. For each biomarker, variants were mapped to rsIDs and imported using read_exposure_data(). Variants with p-values < 5×10^-8^ were selected for each and clumped using default parameters using clump_data(). AD risk GWAS summary statistics were extracted for overlapping variants (based on rsID) using read_outcome_data() and effects were harmonized across exposure and outcome using harmonise_data(). MR was performed using mr() using five different methods: MR Egger, Weighted Median, Inverse Variance Weighted, Simple Mode, and Weighted Mode, with inverse-variance weighted statistics being prioritized. To test for horizontal pleiotropy, the significance of the MR-Egger intercept was calculated for each comparison.

#### Genetic correlation and shared genetic architecture

We also sought to investigate whether the genetic factors associated with the endophenotypes showed a similar pattern among the measures or had any correlation with other complex traits. To answer this, we analyzed genetic covariance using GNOVA (genetic covariance analyzer)^85^. Covariance was assessed against AD risk and 19 other human health related traits. These included brain health-related traits such as brain amyloidosis, hippocampal volume and intracranial volume; metal health conditions like bipolar disorder, depression and schizophrenia; and other neurodegenerative disorders like frontotemporal dementia (FTD), amyotrophic lateral sclerosis (ALS) and multiple sclerosis, among others. Prior to analysis, summary statistics files were prepared using “munge.py” pipeline as recommended by the tool. Calculations were performed using default parameters. Covariance was evaluated both including and excluding *APOE* gene region. Significance was defined as FDR corrected *p* < 0.05. Findings from GNOVA were further verified using complimentary LDSC tool.

#### Biological inference from pathway analysis

We performed pathway analysis using the “ReactomePA” (v1.48) package in R using overrepresentation analysis^86^. Genes associated with all suggestive significant independent variants (*p* < 1 × 10^−5^) annotated by FUMA (excluding APOE region; *N*_Aβ42_ = 55; *N*_t-tau_ = 107; *N*_p-tau181_ = 85) were used as input. Genes from the *APOE* region were excluded as their strong and well documented association with lipid pathways tends to skew the results and may overshadow other important associations. To further identify diseases associated with the genes of interest, we performed enrichment analysis with DisGeNET using the “DOSE” (v3.30) R package^87,88^. A *p* < 0.05 was used as significance threshold. Protein-Protein interactions (PPI) were interrogated using STRING-db^89^ with a significant enrichment association defined by *p* < 0.05. The candidate genes from gene prioritization were interrogated to see if they were enriched for any interactions.

### Reporting summary

Further information on research design is available in the Nature Portfolio Reporting Summary linked to this article.

## Supplementary information

Supplementary Information

Peer Review File

Description of Additional Supplementary Files

Supplementary Data 1

Supplementary Data 2

Supplementary Data 3

Supplementary Data 4

Supplementary Data 5

Supplementary Data 6

Supplementary Data 7

Supplementary Data 8

Supplementary Data 9

Supplementary Data 10

Supplementary Data 11

Supplementary Data 12

Supplementary Data 13

Supplementary Data 14

Supplementary Data 15

Supplementary Data 16

Supplementary Data 17

Supplementary Data 18

Supplementary Data 19

Supplementary Data 20

Supplementary Data 21

Supplementary Data 22

Reporting Summary

## Source data

Source Data

## Data Availability

The GWAS meta-analysis summary statistics generated in this study have been deposited in the NIAGADS database under accession code NG00191 and GWAS catalog (GCST90726396 [https://www.ebi.ac.uk/gwas/studies/GCST90726396]; GCST90726397; GCST90726398). The files are also available to download from the Washington University in St. Louis NeuroGenomics and Informatics Center web portal (https://neurogenomics.wustl.edu/open-science/raw-data/). The raw data from ACE, ALFA+, EADB, and EMIF are restricted in accordance with European Union law on participant privacy (General Data Protection Regulation). EPAD, MISSION-AD and Janssen are protected data are not available due to data privacy laws. Please contact individual study for their respective summary statistics access: ACE (http://www.fundacioace.com/en; vfernandez@fundacioace.org), ALFA+ (https://www.barcelonabeta.org/en/research/alfa; fanastasi@barcelonabeta.org), EADB (s.j.vanderlee@amstrdamumc.nl), EPAD (https://ep-ad.org/index.php/open-source-data/; Riccardo.Marioni@ed.ac.uk), EMIF-AD (https://emif-catalogue.eu; lars.bertram@uni-luebeck.de), MISSION-AD(Mike_Nagle@eisai.com) and Janssen (qingqin.li@chdifoundation.org). For any other data-related questions, please contact timsinaj@wustl.edu or cruchagac@wustl.edu. Data access requests will be reviewed, and a response will be provided within one month of submission. Human Research Protection Office approval and an approved data request are needed to access the summary statistics and individual-level data. Data generated and analyzed in this study can be accessed via Aβ42 meta-analysis (https://wustl.box.com/s/nfexw54o37smdq84lz1inpqduqcf7ofa), tau meta-analysis (https://wustl.box.com/s/pydeqc87yke2ejvve5mrh9quyaikgq2p) and p-tau181meta-analysis (https://wustl.box.com/s/nmyjzql5awxu7qu57m33rkcvq1w3njj8). Data associated with Supplementary Figs. can be accessed using the following links: Non-European ancestry summary statistics (Aβ42 summary statistics, https://wustl.box.com/s/vyg6jy8utsusfgt0hq982l9sxwelyz6n; tau summary statistics, https://wustl.box.com/s/aij5urrr1p7y6xh9z3bmf3lzycukl3cn; p-tau181 summary statistics, https://wustl.box.com/s/1k8gnrcr3q0molmni9zmwh7ukofzcmil), MTAG (https://wustl.box.com/s/ktydbtjiqnfeta3kjll0ldfa8bywdrzy), Case-Control summary statistics (control Aβ42, https://wustl.box.com/s/fe7p346x1b0qbpb8x6ktr3tfe188zv1f; control tau, https://wustl.box.com/s/sw4c8a8oau6toxauwclfireuvb3xqdov; control p-tau181, https://wustl.box.com/s/nufk3uh6xtqokd24h1hupl7qed5pbuqc; case Aβ42, https://wustl.box.com/s/g3ouyb6auhbjfdevun4602iv6fth1vf9; case tau, https://wustl.box.com/s/ymjc7jwggdeiwnon6easnmcofdo5h2u7; case p-tau181, https://wustl.box.com/s/rdnhg8kn8i1u5p79h2nj2vt0lds5ewrr) and Interaction GWAS (Aβ42 summary statistics, https://wustl.box.com/s/i15maobgqjyki02crje5tdp18m0wadgy; tau summary statistics, https://wustl.box.com/s/5fdxh3ac8v4xgymu61j6xcqx2o0d2o0c; p-tau181 summary statistics, https://wustl.box.com/s/um5mydm5n78bqk5n6da4kbqrvfhkj4xg). The source data underlying Supplementary Figs. 10 and 20 is provided as a source data file. Source data underlying Supplementary Fig. 16 is available at Github (https://github.com/NeuroGenomicsAndInformatics/GWAS-project) and on Zenodo (https://zenodo.org/records/17780074). Source data for this manuscript is available in the Supplementary materials and in the Source Data file. Source data are provided with this paper.

## Code availability

We used publicly available software for all analyses in this study. This included: PLINK 2 version 2.00a3.6LM for all single variant analysis, METAL for meta-analysis, mtag (Multi-Trait Analysis of GWAS; https://github.com/JonJala/mtag) for pleiotropy analysis, TwoSampleMR R package (version 0.5.8) to perform MR and the coloc R package (version 5.2.2) to perform colocalization. Pathway analysis was performed using the clusterProfiler (version 4.8.2), org.Hs.eg.db (version 3.17.0), DOSE (version 3.26.1) and ReactomePA (version 1.44.0) R packages. LD Score Regression (LDSC; https://github.com/bulik/ldsc) and GNOVA were used for genetic covariance test. Variant annotation and gene-based analysis were performed using ANNOVAR and MAGMA as part of FUMA pipeline (https://fuma.ctglab.nl/). Detailed code used in this project is available on github (https://github.com/NeuroGenomicsAndInformatics/GWAS-project) and on Zenodo (https://zenodo.org/records/17780074).
