# Supplementary material for: GWAS meta-analysis of cerebrospinal fluid Alzheimer’s biomarkers reveals loci regulating lipids, brain volume and autophagy
Source: Nat Commun. 2026 Apr 21;17:7385. doi: 10.1038/s41467-026-71682-8 (PMC13402690; doi:10.1038/s41467-026-71682-8)
Supplement: Supplementary file 1 — Supplementary Information [file 41467_2026_71682_MOESM1_ESM.pdf]

GWAS meta-analysis of Cerebrospinal fluid Alzheimer's biomarkers reveals loci regulating lipids, brain volume and autophagy

Table of Contents

Supplementary Figures……………………………………….page 2-21.
Additional Cohort Details…………………………………….page 22-29

A.

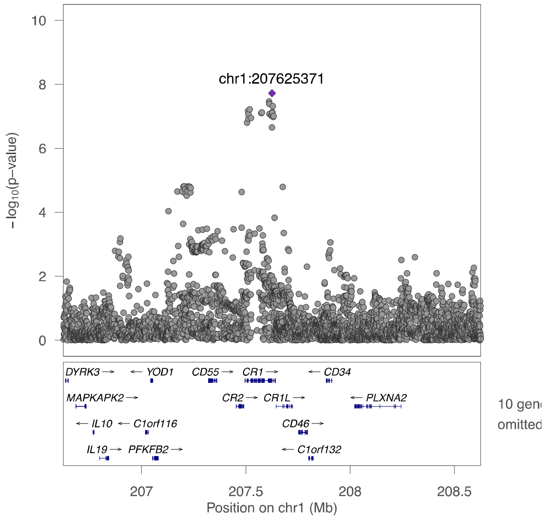

chr1:207625371:T:G

Cohort	N	BETA	SE	P
META	18024	-0.07	0.01	1.896e-08
Joint 22 Cohort	6729	-0.08	0.02	2.02e-04
EADB	7500	-0.08	0.02	8.65e-05
EPAD	1369	-0.01	0.05	8.52e-01
ACE	1301	-0.05	0.05	2.84e-01
EMIF	667	-0.08	0.06	2.40e-01
EISAI	458	-0.13	0.09	1.19e-01

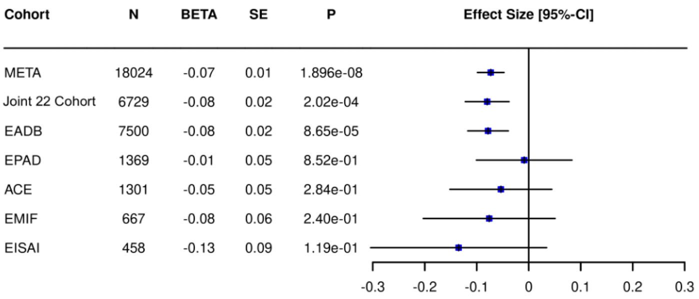

B.

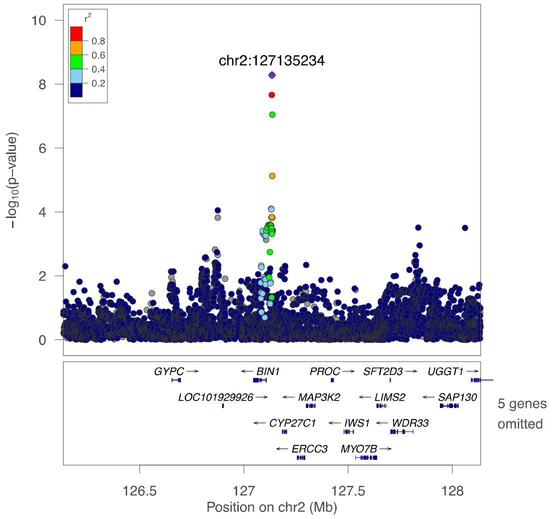

chr2:127135234:C:T

Cohort	N	BETA	SE	P
META	18865	-0.06	0.01	8.293e-09
Joint 22 Cohort	6729	-0.05	0.02	2.40e-03
EADB	7500	-0.06	0.02	1.89e-04
EPAD	1369	-0.10	0.04	7.19e-03
ACE	1301	-0.08	0.04	5.12e-02
EMIF	667	0.00	0.05	9.23e-01
EISAI	458	-0.02	0.06	8.01e-01
ALFA	384	-0.13	0.08	8.34e-02
Janssen Cohort 1 (Bapineuzumab)	268	-0.10	0.09	2.60e-01
Janssen Cohort 2 (BACEi)	189	0.04	0.11	7.19e-01

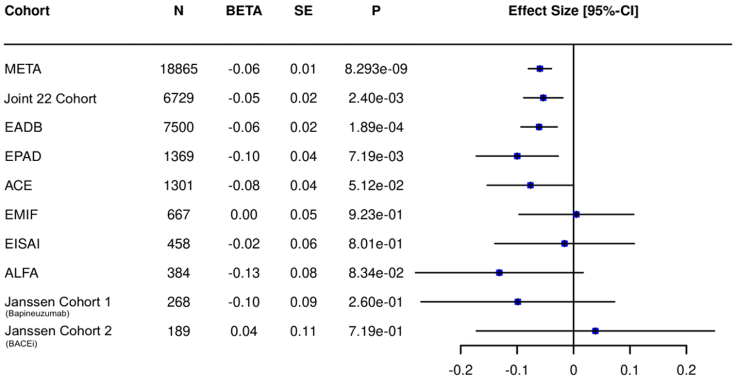

Supplementary Figure 1: *Locus zoom and forest plot for significant loci associated with Aβ42 from meta-analysis.* ***(A)*** *Variant rs1077933 located within CR1 gene region.* ***(B)*** *Variant rs6733839 located within BIN1 gene region. Forest plot shows the effect size, standard error, p-value and sample size within each cohort included in meta-analyses as well as meta-analysis result. The dot and whiskers represent Effect size and its corresponding 95% confidence interval (L95=lower end, U95=upper end) within each cohort. META represents meta-analysis result from METAL. Source Data for this figure is available on Aβ42 meta-analysis (https://wustl.box.com/s/nfexw54o37smdq84lz1inpqduqcf7ofa).*

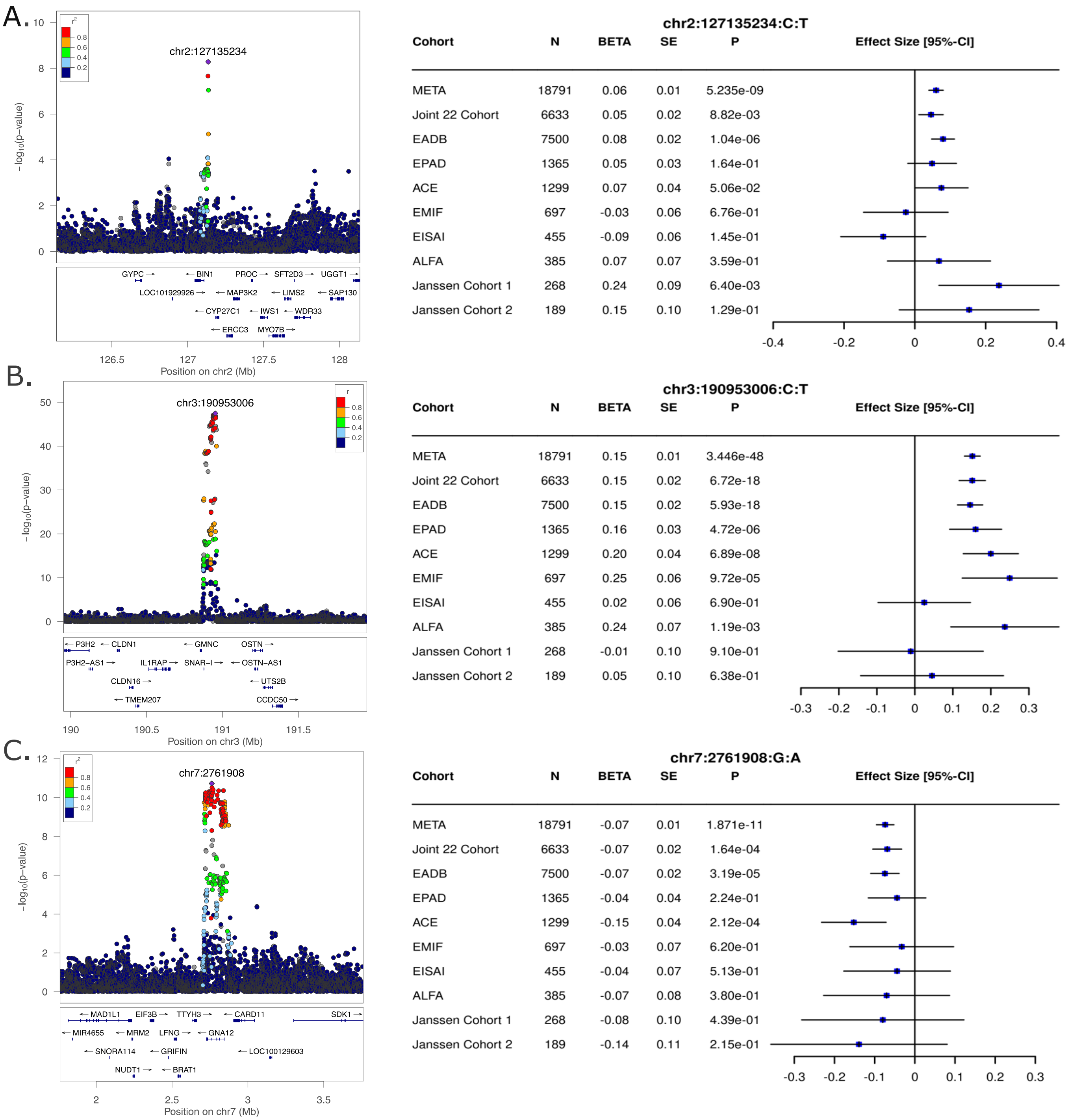

Supplementary Figure 2: *Locus zoom and forest plot for significant loci associated with t-tau from meta-analysis.* ***(A)*** *Variant rs6733839 located within BIN1 gene region.* ***(B)*** *Variant rs35327527 located within GMNC/CCDC50 gene region.* ***(C)*** *Variant rs798490 located within GNA12/AMZ1 gene region. Forest plot shows the effect size, standard error, p-value and sample size within each cohort included in meta-analyses as well as meta-analysis result. The dot and whiskers represent Effect size and its corresponding 95% confidence interval (L95=lower end, U95=upper end) within each cohort. META represents meta-analysis result from METAL. Source Data for this figure is available on* tau meta-analysis *(https://wustl.box.com/s/pydeqc87yke2ejvve5mrh9quyaikgq2p)*

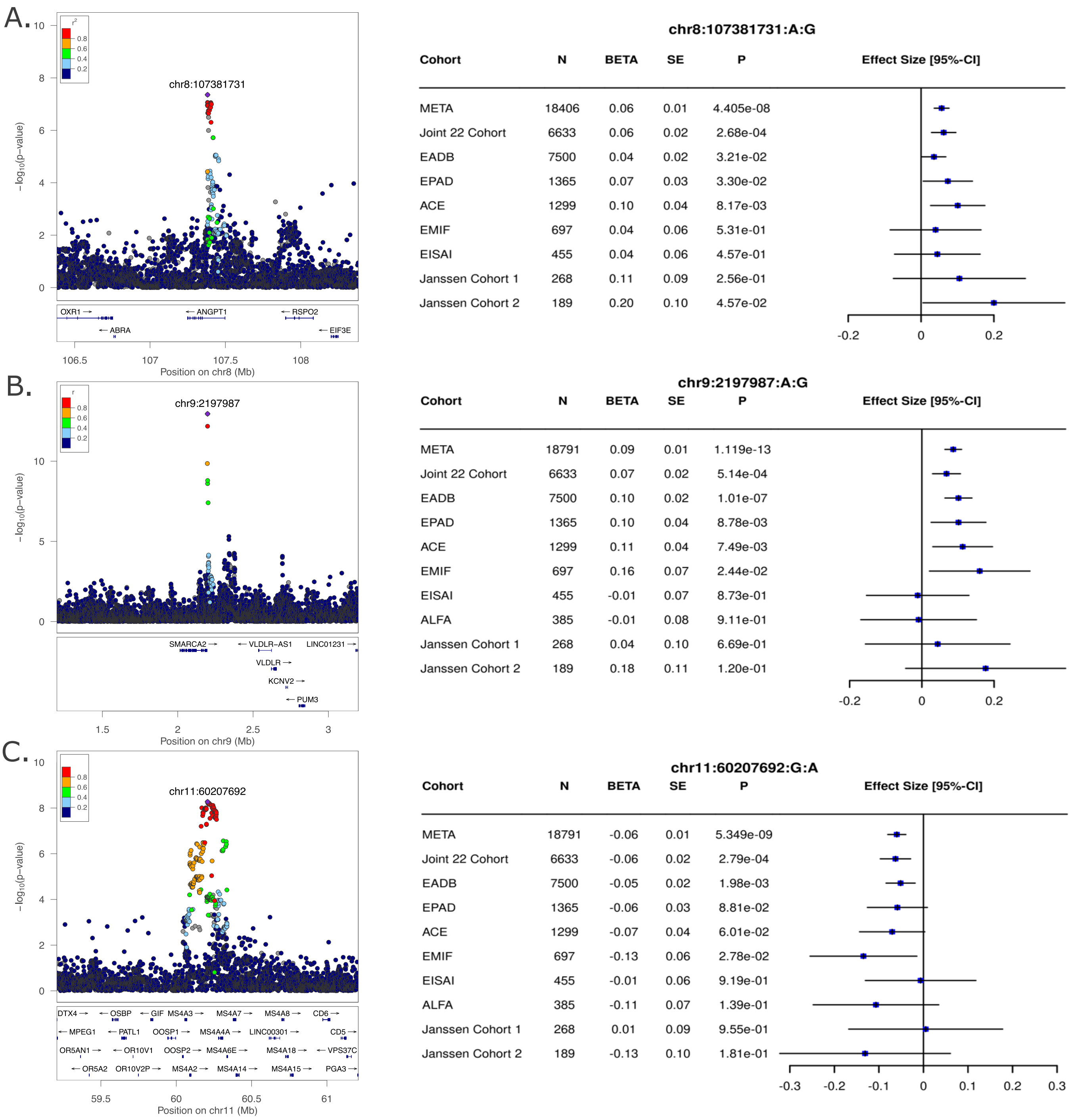

chr8:107381731:A:G

Cohort	N	BETA	SE	P
META	18406	0.06	0.01	4.405e-08
Joint 22 Cohort	6633	0.06	0.02	2.68e-04
EADB	7500	0.04	0.02	3.21e-02
EPAD	1365	0.07	0.03	3.30e-02
ACE	1299	0.10	0.04	8.17e-03
EMIF	697	0.04	0.06	5.31e-01
EISAI	455	0.04	0.06	4.57e-01
Janssen Cohort 1	268	0.11	0.09	2.56e-01
Janssen Cohort 2	189	0.20	0.10	4.57e-02

chr9:2197987:A:G

Cohort	N	BETA	SE	P
META	18791	0.09	0.01	1.119e-13
Joint 22 Cohort	6633	0.07	0.02	5.14e-04
EADB	7500	0.10	0.02	1.01e-07
EPAD	1365	0.10	0.04	8.78e-03
ACE	1299	0.11	0.04	7.49e-03
EMIF	697	0.16	0.07	2.44e-02
EISAI	455	-0.01	0.07	8.73e-01
ALFA	385	-0.01	0.08	9.11e-01
Janssen Cohort 1	268	0.04	0.10	6.69e-01
Janssen Cohort 2	189	0.18	0.11	1.20e-01

chr11:60207692:G:A

Cohort	N	BETA	SE	P
META	18791	-0.06	0.01	5.349e-09
Joint 22 Cohort	6633	-0.06	0.02	2.79e-04
EADB	7500	-0.05	0.02	1.98e-03
EPAD	1365	-0.06	0.03	8.81e-02
ACE	1299	-0.07	0.04	6.01e-02
EMIF	697	-0.13	0.06	2.78e-02
EISAI	455	-0.01	0.06	9.19e-01
ALFA	385	-0.11	0.07	1.39e-01
Janssen Cohort 1	268	0.01	0.09	9.55e-01
Janssen Cohort 2	189	-0.13	0.10	1.81e-01

Supplementary Figure 3: *Locus zoom and forest plot for significant loci associated with t-tau from meta-analysis.* ***(A)*** *Variant rs1654723 located within ANGPT1 gene region.* ***(B)*** *Variant rs57263785 located within SMARCA2 gene region.* ***(C)*** *Variant rs7928895 located within MS4A family gene region. Forest plot shows the effect size, standard error, p-value and sample size within each cohort included in meta-analyses as well as meta-analysis result. The dot and whiskers represent Effect size and its corresponding 95% confidence interval (L95=lower end, U95=upper end) within each cohort. META represents meta-analysis result from METAL. Source Data for this figure is available on tau meta-analysis (https://wustl.box.com/s/pydeqc87yke2ejvve5mrh9quyaikgq2p).*

A.

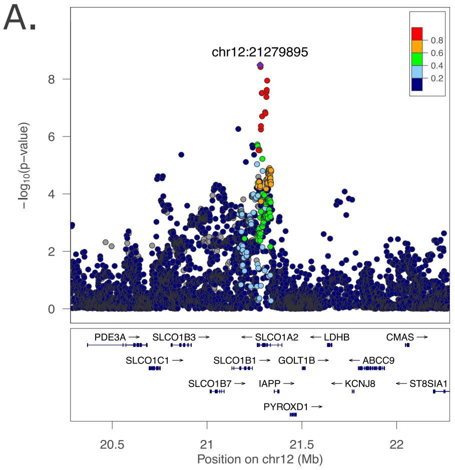

chr12:21279895:C:T

Cohort	N	BETA	SE	P
META	18645	0.07	0.01	3.26e-09
Joint 22 Cohort	6487	0.05	0.02	1.29e-02
EADB	7500	0.07	0.02	1.69e-04
EPAD	1365	0.10	0.04	6.64e-03
ACE	1299	0.09	0.04	3.71e-02
EMIF	697	0.08	0.07	2.16e-01
EISAI	455	0.06	0.07	3.84e-01
ALFA	385	0.11	0.08	1.56e-01
Janssen Cohort 1	268	0.05	0.10	6.31e-01
Janssen Cohort 2	189	0.12	0.11	2.51e-01

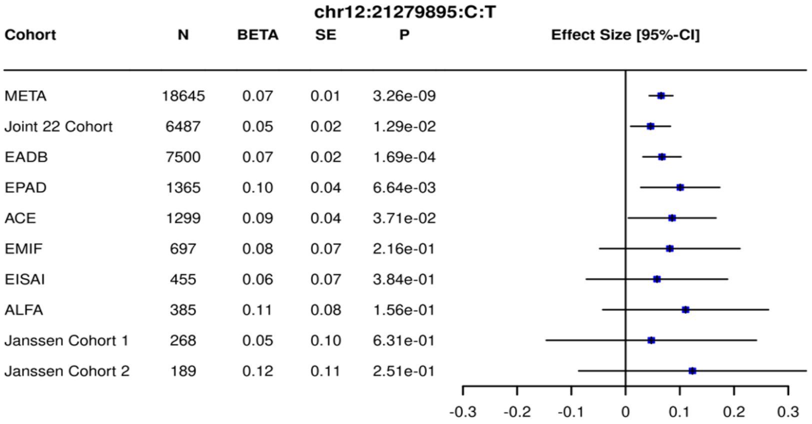

B.

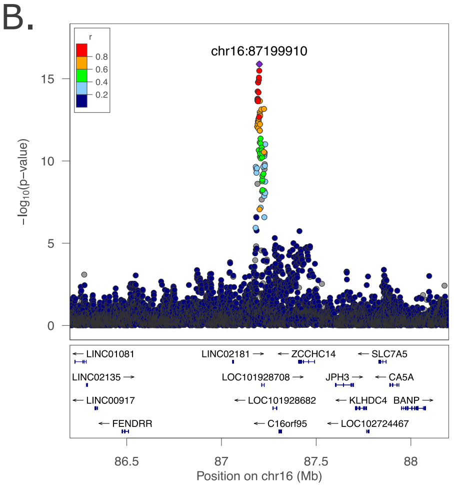

chr16:87199910:G:A

Cohort	N	BETA	SE	P
META	18406	0.09	0.01	1.302e-16
Joint 22 Cohort	6633	0.08	0.02	1.61e-06
EADB	7500	0.09	0.02	1.29e-08
EPAD	1365	0.03	0.03	3.65e-01
ACE	1299	0.13	0.04	1.00e-03
EMIF	697	0.12	0.06	5.87e-02
EISAI	455	0.01	0.06	8.77e-01
Janssen Cohort 1	268	0.17	0.08	3.71e-02
Janssen Cohort 2	189	0.05	0.10	6.53e-01

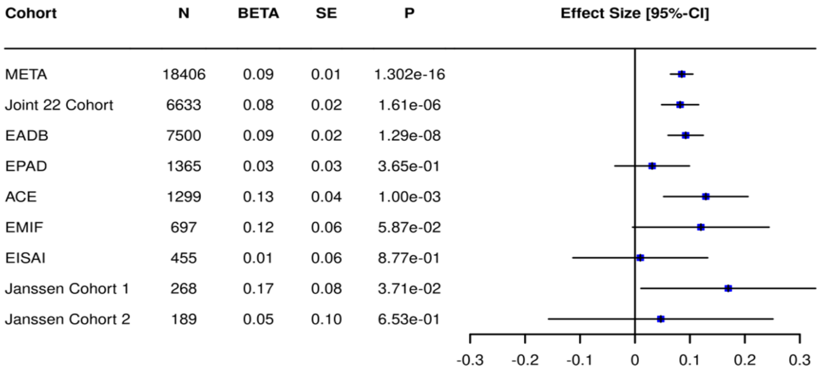

Supplementary Figure 4: *Locus zoom and forest plot for significant loci associated with t-tau from meta-analysis.* ***(A)*** *Variant rs11045930 located within SLCO1A2 gene region.* ***(B)*** *Variant rs4843552 located within C16orf95/MAP1LC3B gene region. Forest plot shows the effect size, standard error, p-value and sample size within each cohort included in meta-analyses as well as meta-analysis result. The dot and whiskers represent Effect size and its corresponding 95% confidence interval (L95=lower end, U95=upper end) within each cohort. META represents meta-analysis result from METAL. Source Data for this figure is available on* tau meta-analysis *(https://wustl.box.com/s/pydeqc87yke2ejvve5mrh9quyaikgq2p).*

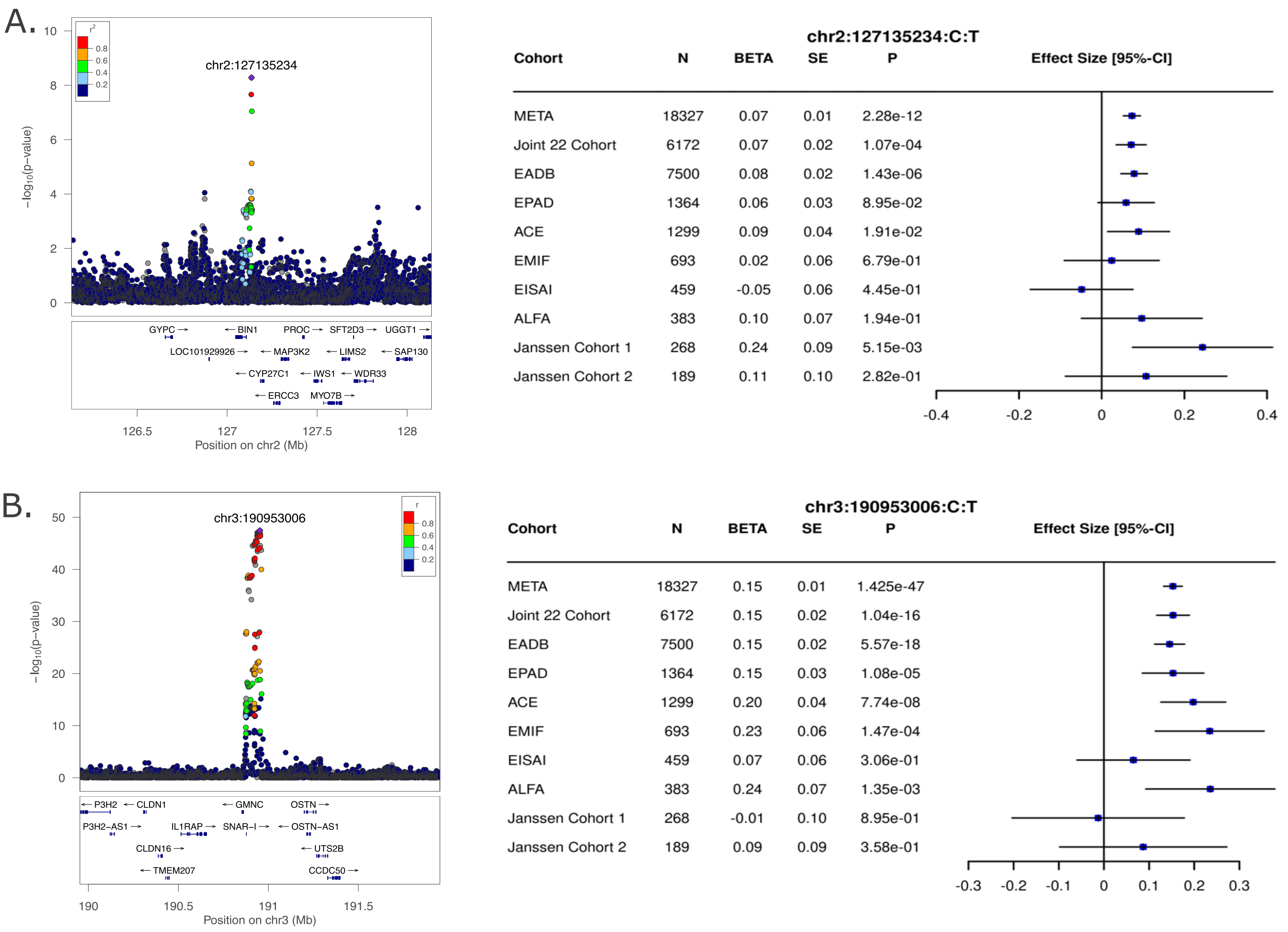

Supplementary Figure 5: *zoom and forest plot for significant loci associated with p-tau181 from meta-analysis.* ***(A)*** *Variant rs6733839 located within BIN1 gene region.* ***(B)*** *Variant rs35327527 located within GMNC/CCDC50 gene region. Forest plot shows the effect size, standard error, p-value and sample size within each cohort included in meta-analyses as well as meta-analysis result. The dot and whiskers represent Effect size and its corresponding 95% confidence interval (L95=lower end, U95=upper end) within each cohort. META represents meta-analysis result from METAL. Source Data for this figure is available on p-tau181 meta-analysis (https://wustl.box.com/s/nmyjzql5awxu7qu57m33rkcvq1w3njj8).*

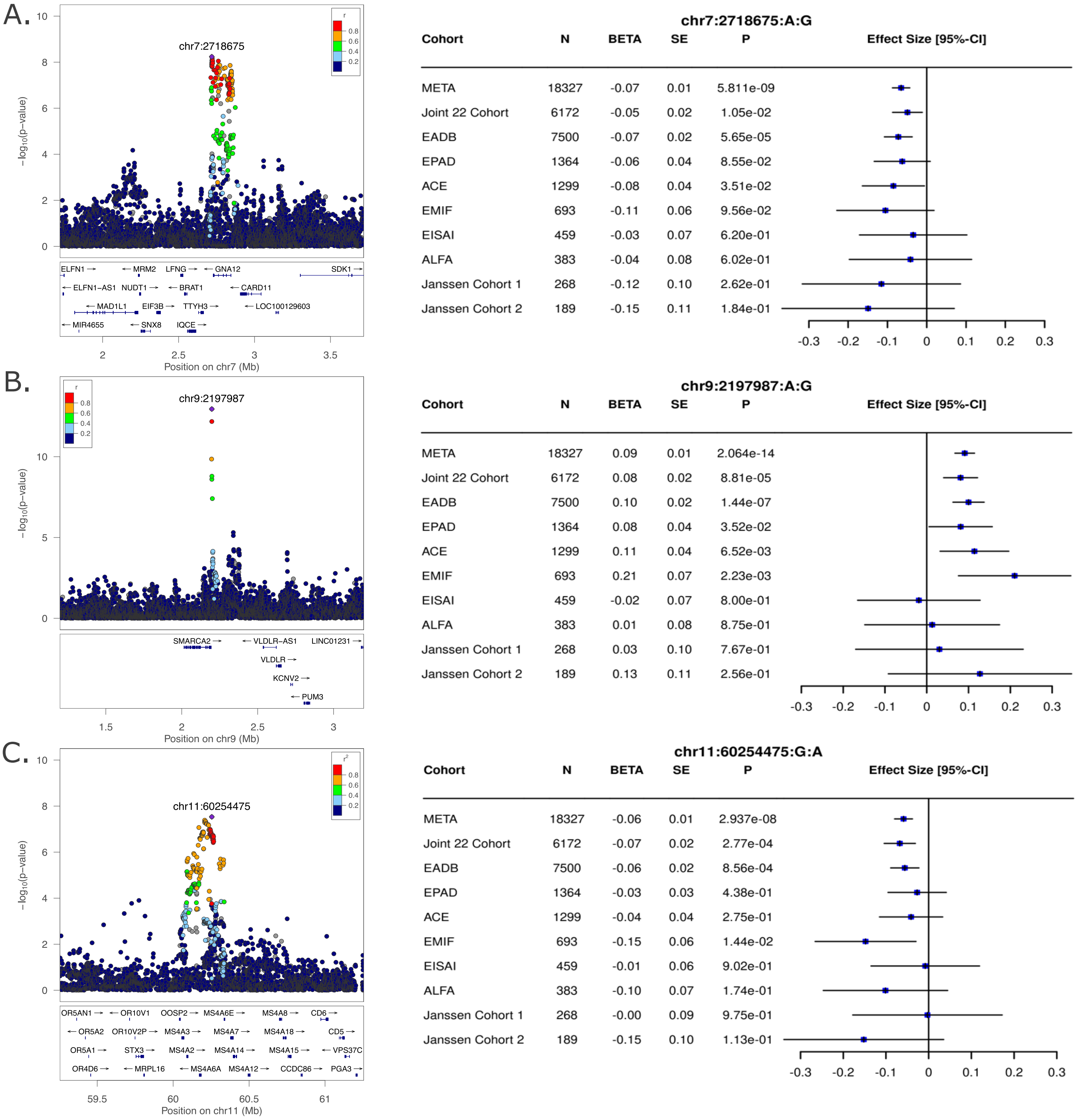

Supplementary Figure 6: *Locus zoom and forest plot for significant loci associated with p-tau181 from meta-analysis.* ***(A)*** *Variant rs798560 located within GNA12/AMZ1 gene region.* ***(B)*** *Variant rs57263785 located within SMARCA2 gene region.* ***(C)*** *Variant rs1582763 located within MS4A family gene region. Forest plot shows the effect size, standard error, p-value and sample size within each cohort included in meta-analyses as well as meta-analysis result. The dot and whiskers represent Effect size and its corresponding 95% confidence interval (L95=lower end, U95=upper end) within each cohort. META represents meta-analysis result from METAL. META represents meta-analysis result from METAL. Source Data for this figure is available on p-tau181 meta-analysis (https://wustl.box.com/s/nmyjzql5awxu7qu57m33rkcvq1w3njj8).*

A.

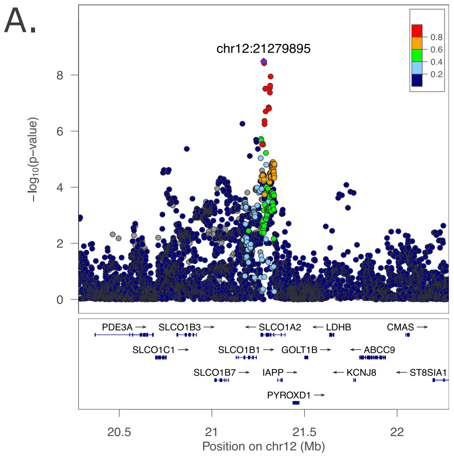

chr12:21279895:C:T

Cohort	N	BETA	SE	P
META	18183	0.07	0.01	6.241e-09
Joint 22 Cohort	6028	0.04	0.02	4.29e-02
EADB	7500	0.07	0.02	7.80e-05
EPAD	1364	0.11	0.04	4.12e-03
ACE	1299	0.08	0.04	5.00e-02
EMIF	693	0.09	0.06	1.37e-01
EISAI	459	0.01	0.07	9.22e-01
ALFA	383	0.14	0.08	7.83e-02
Janssen Cohort 1	268	0.09	0.10	3.72e-01
Janssen Cohort 2	189	0.10	0.11	3.32e-01

Effect Size [95%-CI]

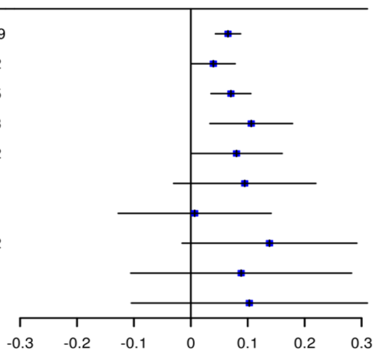

B.

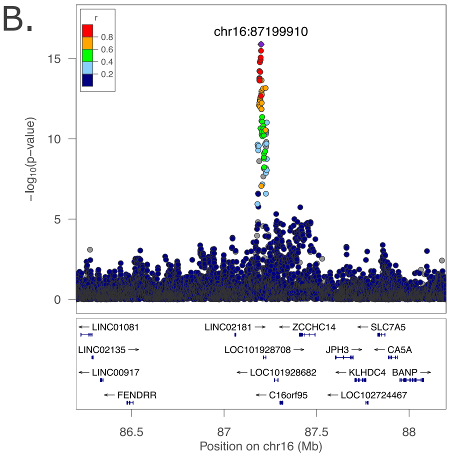

chr16:87199910:G:A

Cohort	N	BETA	SE	P
META	17944	0.08	0.01	7.276e-15
Joint 22 Cohort	6172	0.08	0.02	2.91e-05
EADB	7500	0.09	0.02	9.83e-09
EPAD	1364	0.02	0.03	4.72e-01
ACE	1299	0.10	0.04	1.18e-02
EMIF	693	0.12	0.06	5.21e-02
EISAI	459	0.06	0.06	3.94e-01
Janssen Cohort 1	268	0.17	0.08	4.32e-02
Janssen Cohort 2	189	0.02	0.10	8.43e-01

Effect Size [95%-CI]

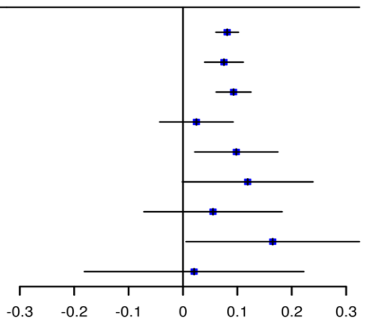

Supplementary Figure 7*: Locus zoom and forest plot for significant loci associated with p-tau181 from meta-analysis.* ***(A)*** *Variant rs11045930 located within SLCO1A2 gene region.* ***(B)*** *Variant* ***r****s4843552 located within C16orf95/MAP1LC3B gene region. Forest plot shows the effect size, standard error, p-value and sample size within each cohort included in meta-analyses as well as meta-analysis result. The dot and whiskers represent Effect size and its corresponding 95% confidence interval (L95=lower end, U95=upper end) within each cohort. META represents meta-analysis result from METAL. META represents meta-analysis result from METAL. Source Data for this figure is available on p-tau181 meta-analysis (https://wustl.box.com/s/nmyjzql5awxu7qu57m33rkcvq1w3njj8).*

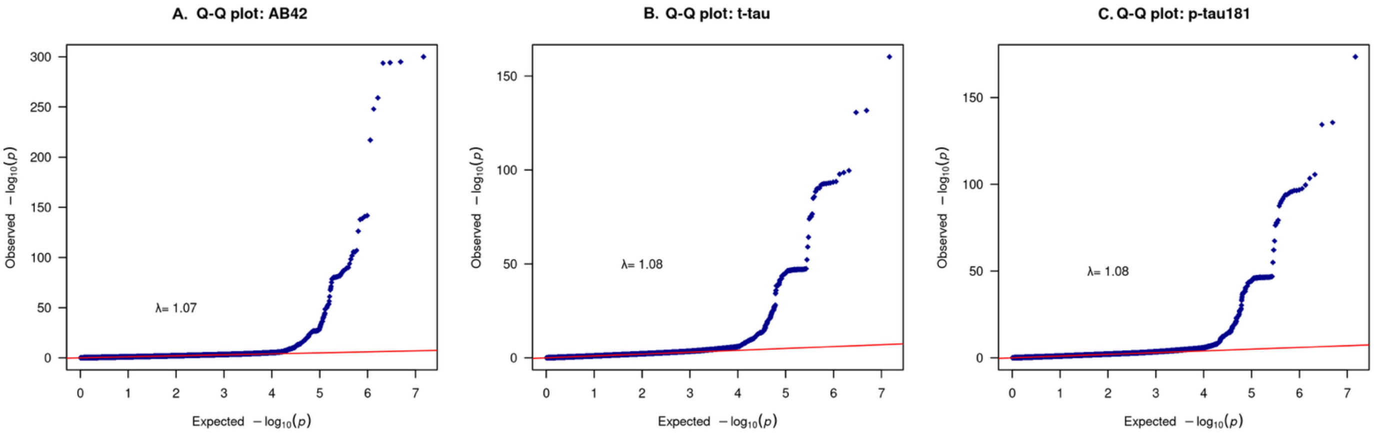

Supplementary Figure 8: *Quantile-Quantile (QQ) plot of observed versus expected –log*$_{10}$*(p-values) for genome-wide association meta-analysis results. Aβ42* ***(A)*** *t-tau**(B)*** *p-tau181**(C).*** *Source Data for this figure is available on AB42 meta-analysis (https://wustl.box.com/s/nfexw54o37smdq84lz1inpqduqcf7ofa), tau meta-analysis (https://wustl.box.com/s/pydeqc87yke2ejvve5mrh9quyaikgq2p) and p-tau181 meta-analysis (https://wustl.box.com/s/nmyjzql5awxu7qu57m33rkcvq1w3njj8).*

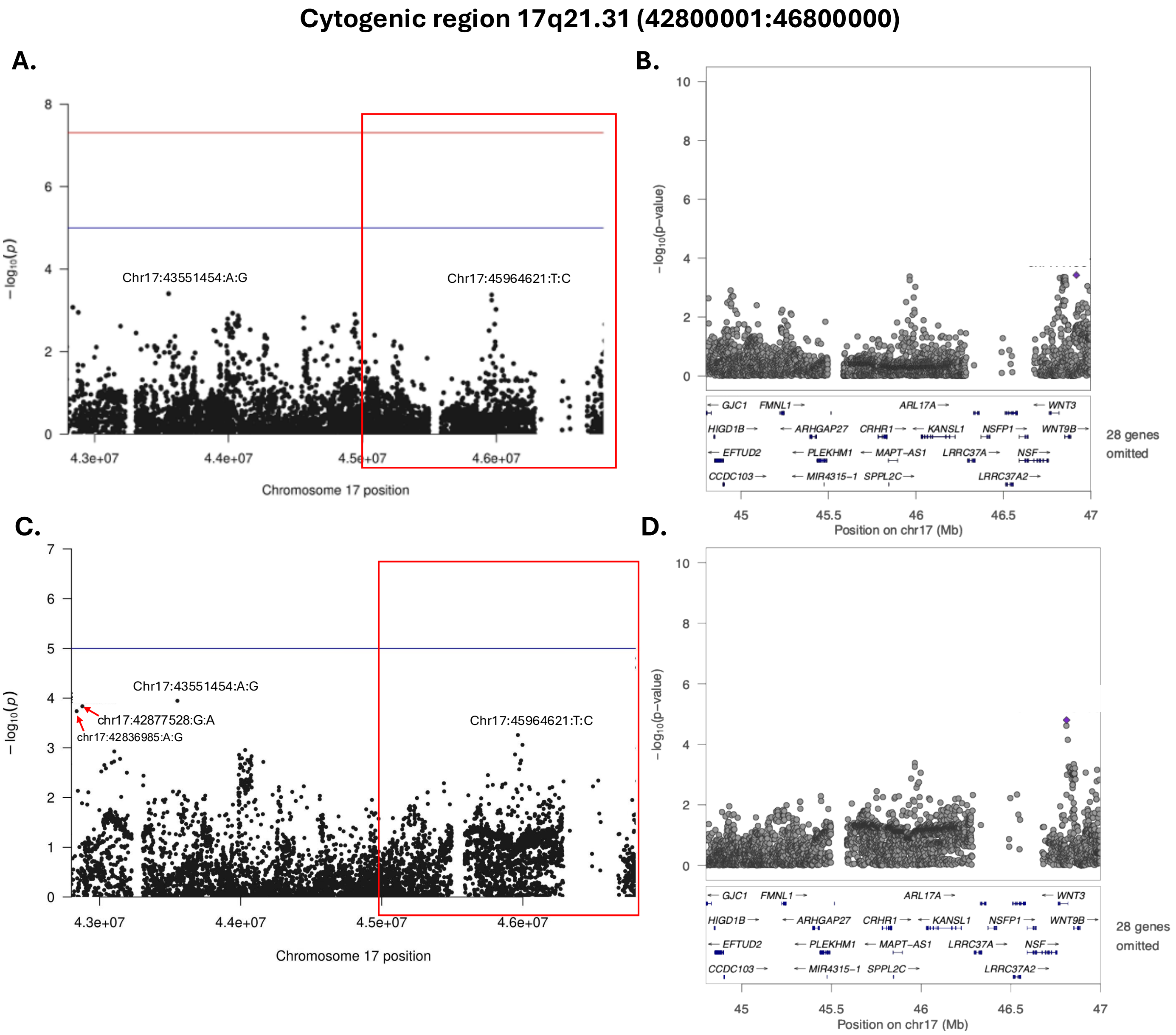

Supplementary Figure 9: *Plot showing association of chromosome 17 inversion locus containing both MAPT haplotypes with t-tau and p-tau. (A-B) Manhattan and locus zoom plot showing association of the region with t-tau. No genome or suggestive significant association observed. Locus zoom covers the area within the red boundary shown in Manhattan plot. (C-D) Manhattan and locus zoom plot showing association of the region with t-tau. No genome or suggestive significant association observed. Locus zoom covers the area within the red boundary shown in Manhattan plot. Source Data for this figure is available on* tau *meta-analysis (https://wustl.box.com/s/pydeqc87yke2ejvve5mrh9quyaikgq2p) and p-tau181 meta-analysis (https://wustl.box.com/s/nmyjzql5awxu7qu57m33rkcvq1w3njj8).*

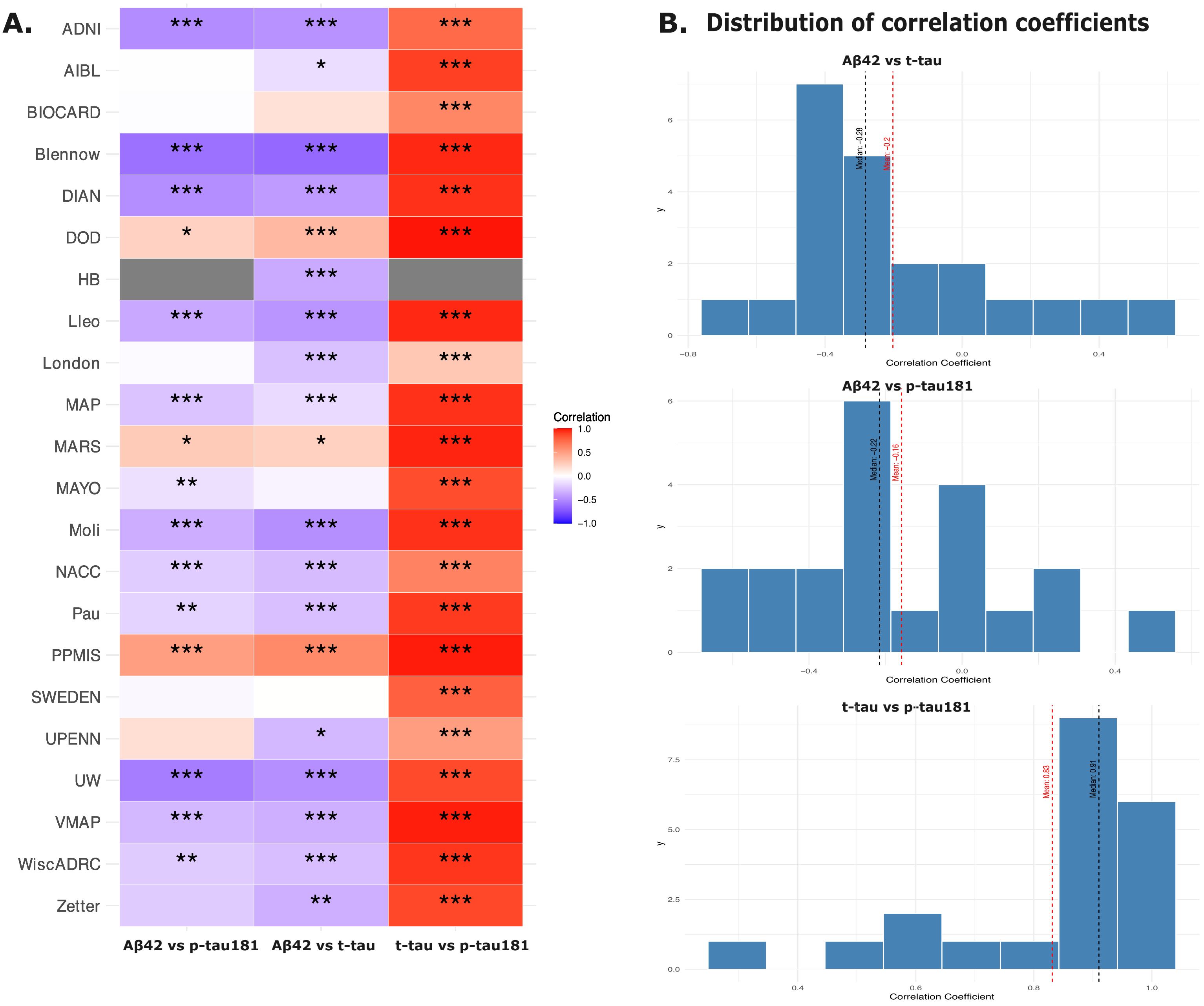

Supplementary Figure 10: *Plot showing phenotypic correlation between biomarkers across different cohorts for which individual level data was available. (A) Heatmap showing correlation per each cohort. "*" represents strength of correlation. "***": p<0.001; "**": p<0.01; "*": p<0.05. Grey tiles represent no available data for comparison (B) Histograms showing distribution of correlation coefficients in each comparison. Black dotted line shows the median and red dotted line represents the mean. Source data are provided as a Source Data file for this figure.*

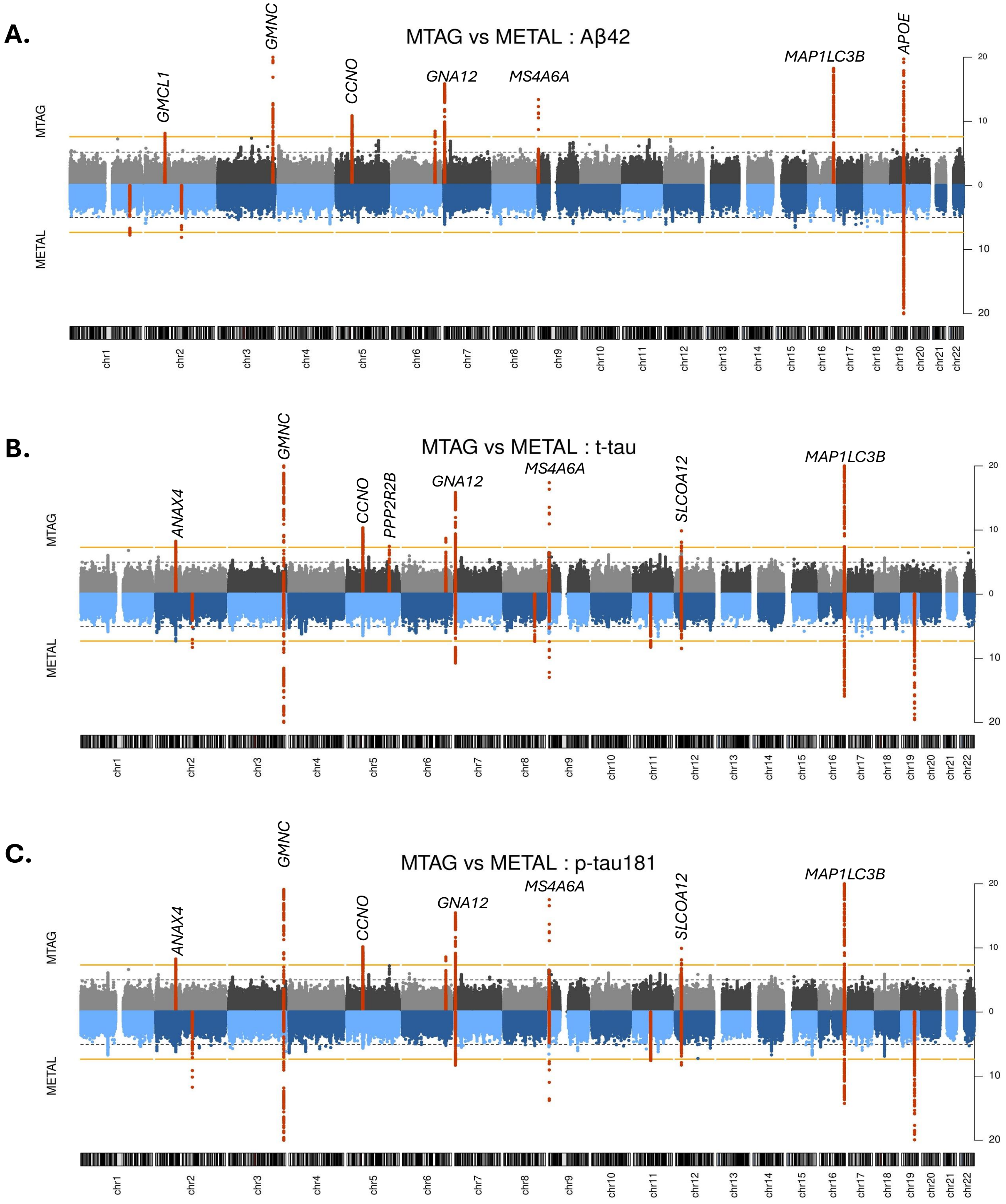

Supplementary Figure 11: *Miami plot showing GWAS results from MTAG vs METAL. The orange line represents genome wide significance. Red highlighted loci are those that passed genome wide significance. Gene names labelled are genes that are either closest or found to be functional for the loci.)* ***(A)*** *Aβ42* ***(B)*** *t-tau* ***(C)*** *p-tau181. Source data for the figure are available at AB42 meta-analysis (https://wustl.box.com/s/nfexw54o37smdq84lz1inpqduqcf7ofa), tau meta-analysis (https://wustl.box.com/s/pydeqc87yke2ejvve5mrh9quyaikgq2p), p-tau181 meta-analysis (https://wustl.box.com/s/nmyjzql5awxu7qu57m33rkcvq1w3njj8 and MTAG (https://wustl.box.com/s/ktydbtjiqnfeta3kjll0ldfa8bywdrzy).*

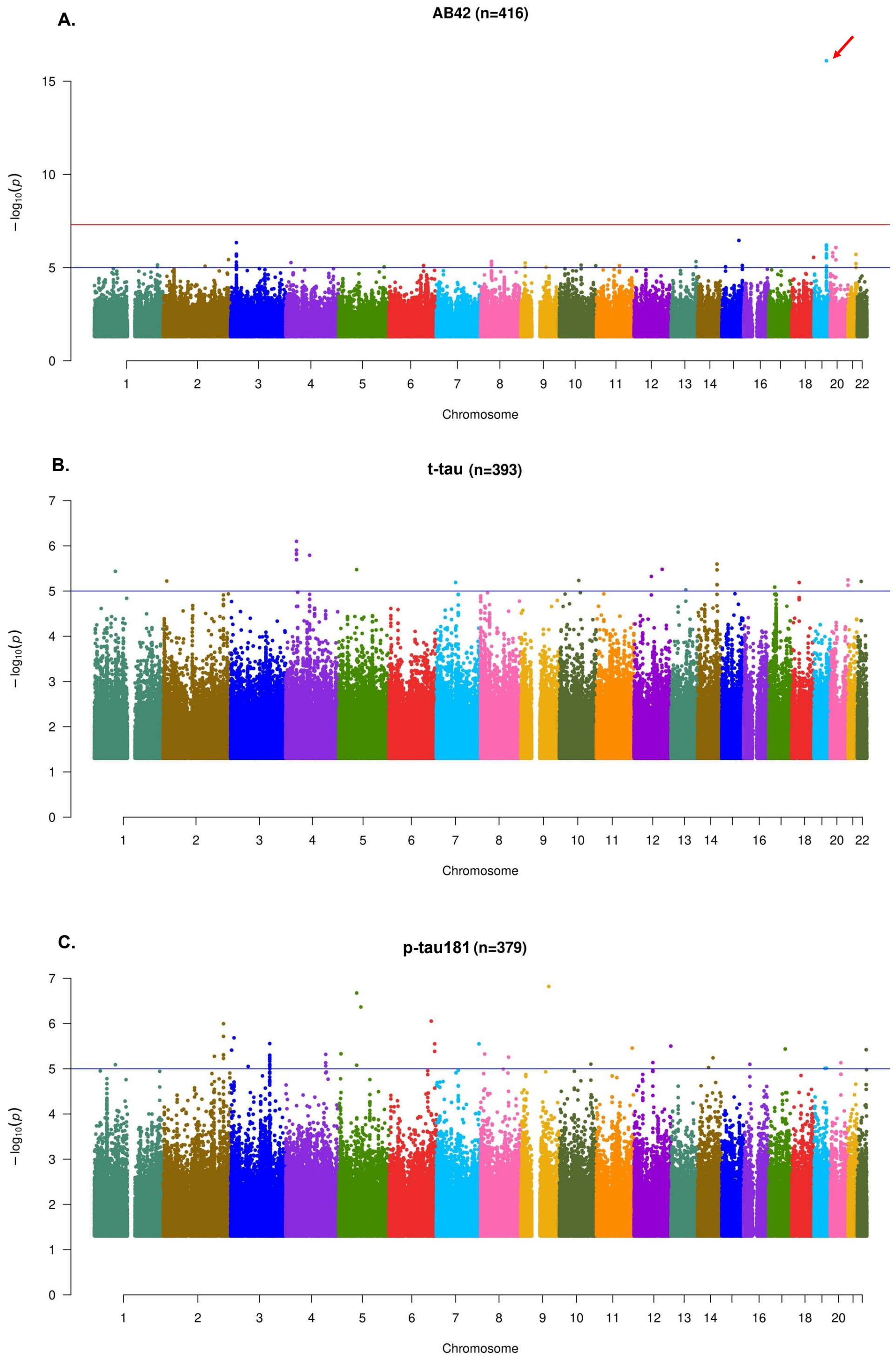

Supplementary Figure 12: *Manhattan plot showing negative log10-transformed p- values from the non-European ancestry GWAS of all three CSF biomarkers. The horizontal lines represent the genome-wide significance threshold, p=5 × 10−8 (orange) and suggestive threshold, p= 1×10−5 (grey).* ***(A)*** *Aβ42* ***(B)*** *t-tau* ***(C)*** *p-tau181. Source data for the figure are available at Aβ42 summary statistics (https://wustl.box.com/s/vyg6jy8utsusfgt0hq982l9sxwelyz6n), tau summary statistics (https://wustl.box.com/s/aij5urrr1p7y6xh9z3bmf3lzycukl3cn) and p-tau181 summary statistics (https://wustl.box.com/s/1k8gnrcr3q0molmni9zmwh7ukofzcmil)*

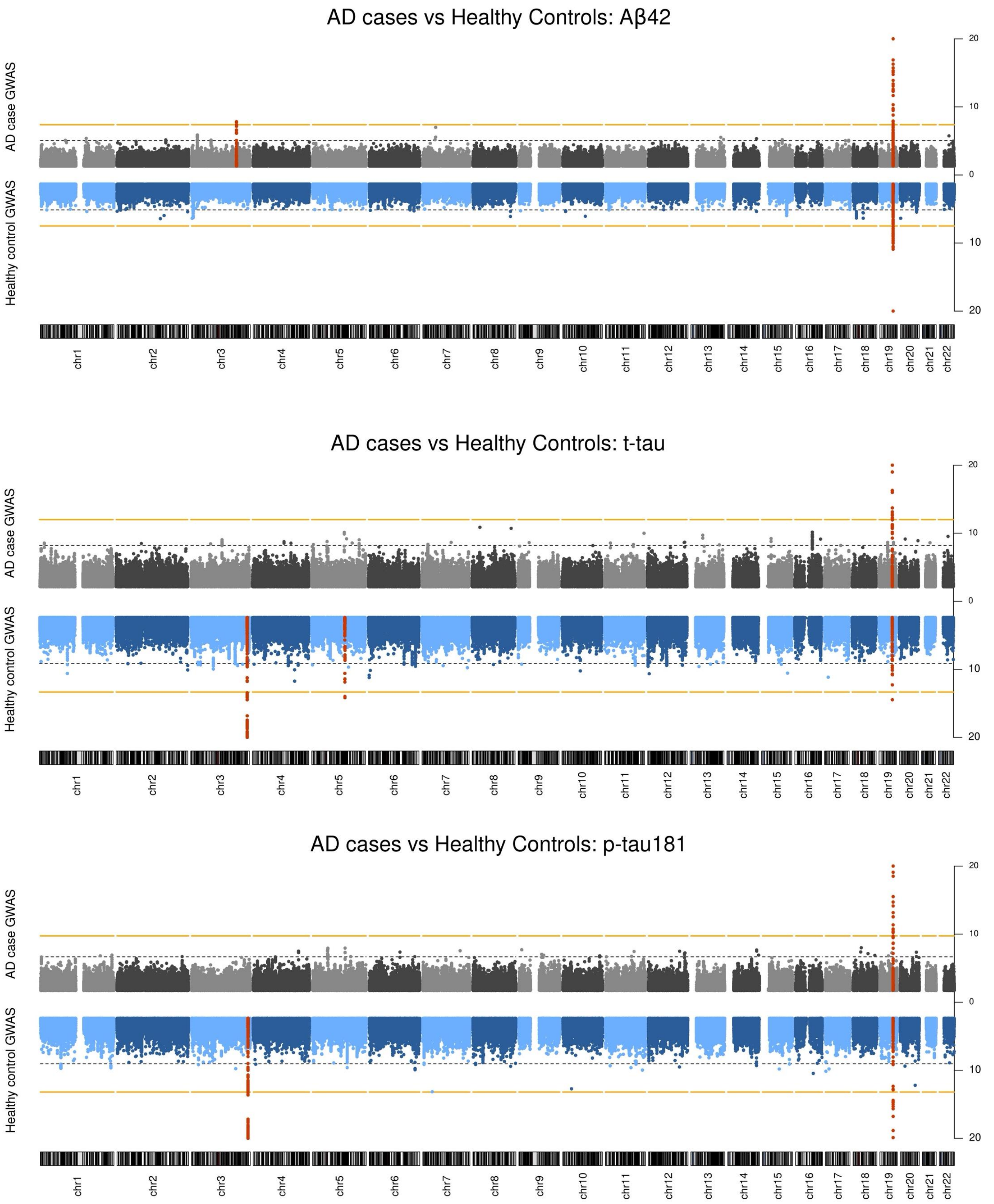

Supplementary Figure 13*: Miami plot showing GWAS results from AD/CO stratified analysis. Plots on top are from AD only GWAS and plot on the bottom are from CO only GWAS. Red highlighted region represents loci that passed genome-wide threshold in corresponding analysis. Source data for the figure are available at control Aβ42 (https://wustl.box.com/s/fe7p346x1b0qbpb8x6ktr3tfe188zv1f), control tau (https://wustl.box.com/s/sw4c8a8oau6toxauwclfireuvb3xqdov), control p-tau181 (https://wustl.box.com/s/nufk3uh6xtqokd24h1hupl7qed5pbuqc), case Aβ42 (https://wustl.box.com/s/g3ouyb6auhbjfdevun4602iv6fth1vf9), case tau (https://wustl.box.com/s/ymjc7jwggdeiwnon6easnmcofdo5h2u7) and case p-tau181 (https://wustl.box.com/s/rdnhg8kn8i1u5p79h2nj2vt0lds5ewrr)*

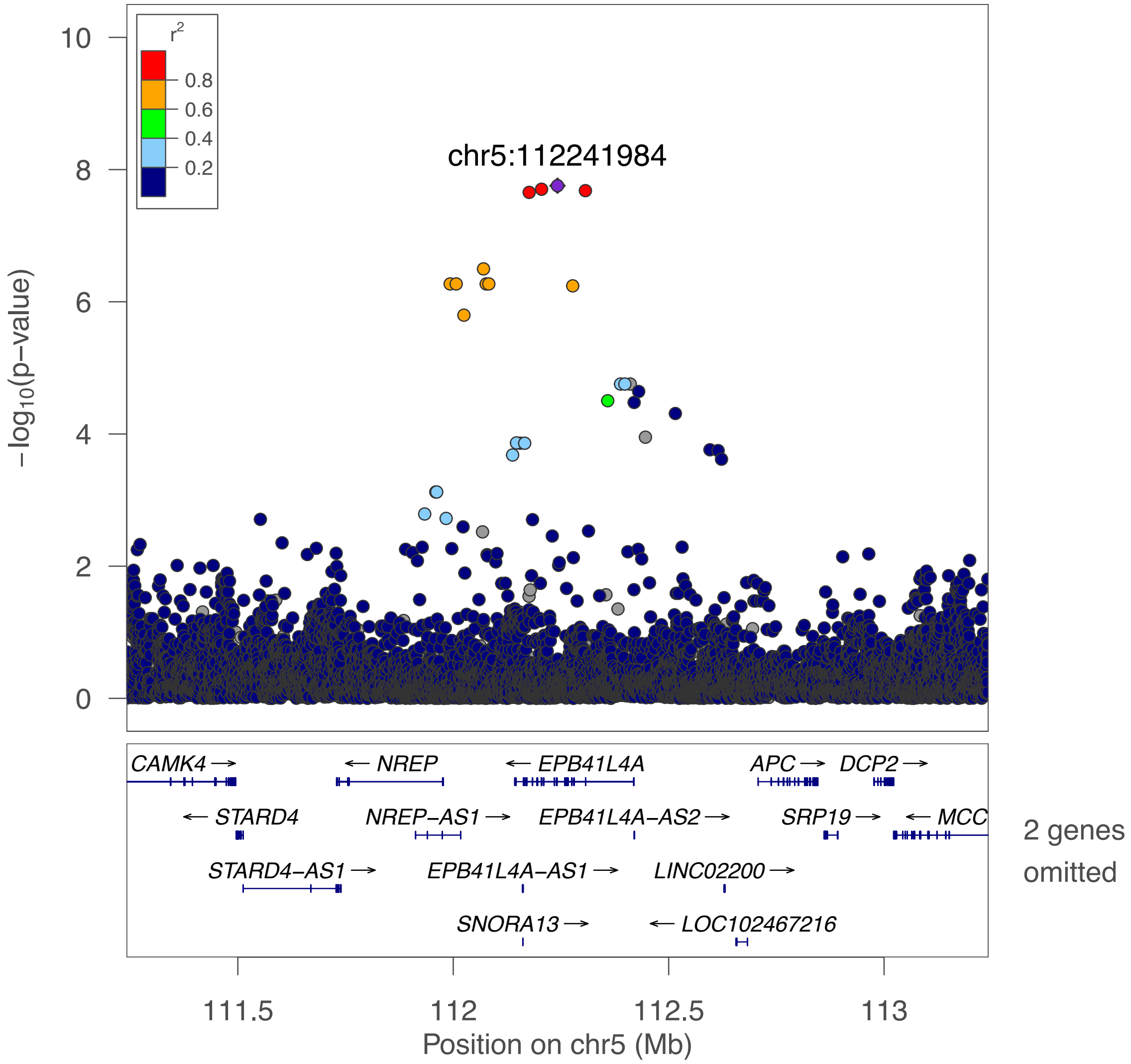

Supplementary Figure 14: *Locus zoom plot showing the chromosome 5 variant associated with t-tau levels in controls. . Source data for the figure is available at* control tau *(https://wustl.box.com/s/sw4c8a8oau6toxauwclfireuvb3xqdov).*

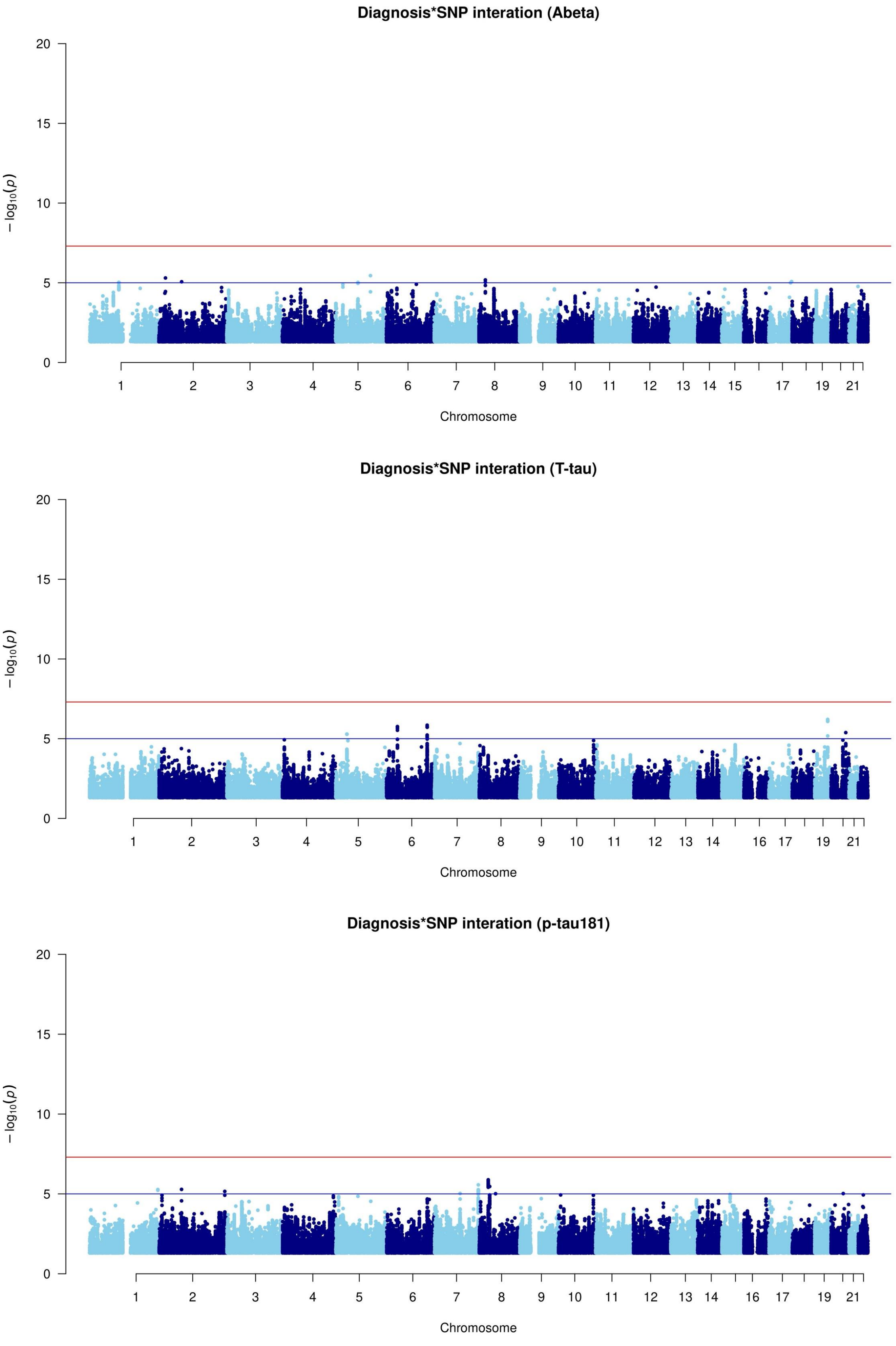

Supplementary Figure 15: *Manhattan plot showing results from interaction term (SNP*Diagnosis) GWAS. Red line represents genome-wide significance threshold. Blue line represents suggestive significance threshold. None of the variants tested passed genome wide significance in the analysis. . Source data for the figure are available at Aβ42 summary statistics (https://wustl.box.com/s/i15maobgqjyki02crje5tdp18m0wadgy), tau summary statistics (https://wustl.box.com/s/5fdxh3ac8v4xgymu61j6xcqx2o0d2o0c) and p-tau181 summary statistics (https://wustl.box.com/s/um5mydm5n78bqk5n6da4kbqrvfhkj4xg).*

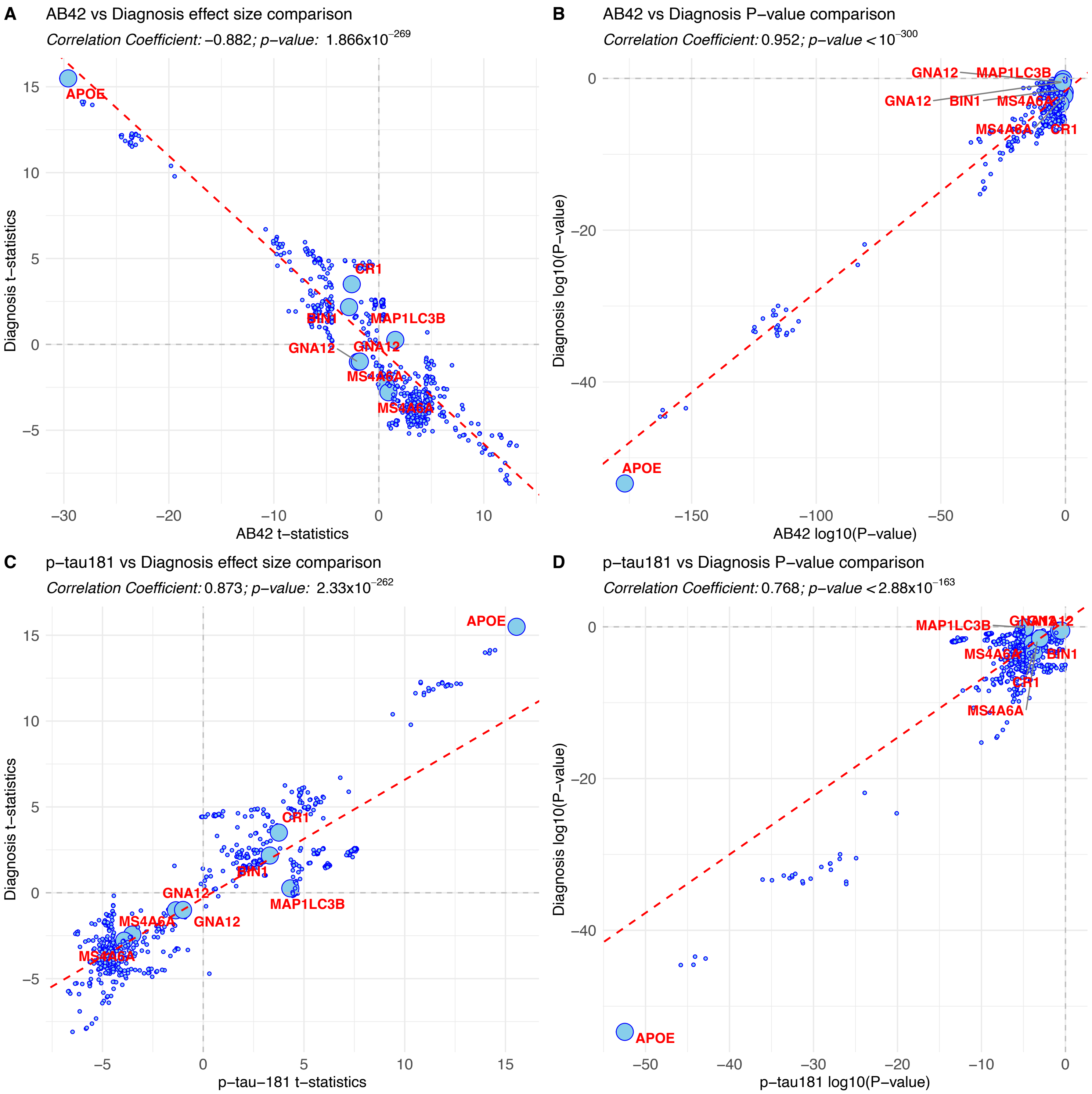

Supplementary Figure 16: *Scatter plot showing correlation between GWAS coefficients.* ***(A)*** *Aβ42 and Diagnosis status t-statistics* ***(B)*** *Aβ42 and Diagnosis status log10 (p-value)* ***(C)*** *p-tau181 and Diagnosis status t-statistics* ***(D)*** *p-tau181 and Diagnosis status log10 (p-value) . Labelled dots show lead variants from meta-analysis that were associated with AD risk. Variants that were nominally significant in either analysis or one of the variants associated with AD risk in meta-analysis was used for comparison. Source data for the figure is available on Github (https://github.com/NeuroGenomicsAndInformatics/GWAS-project) and on Zenodo (https://zenodo.org/records/17780074).*

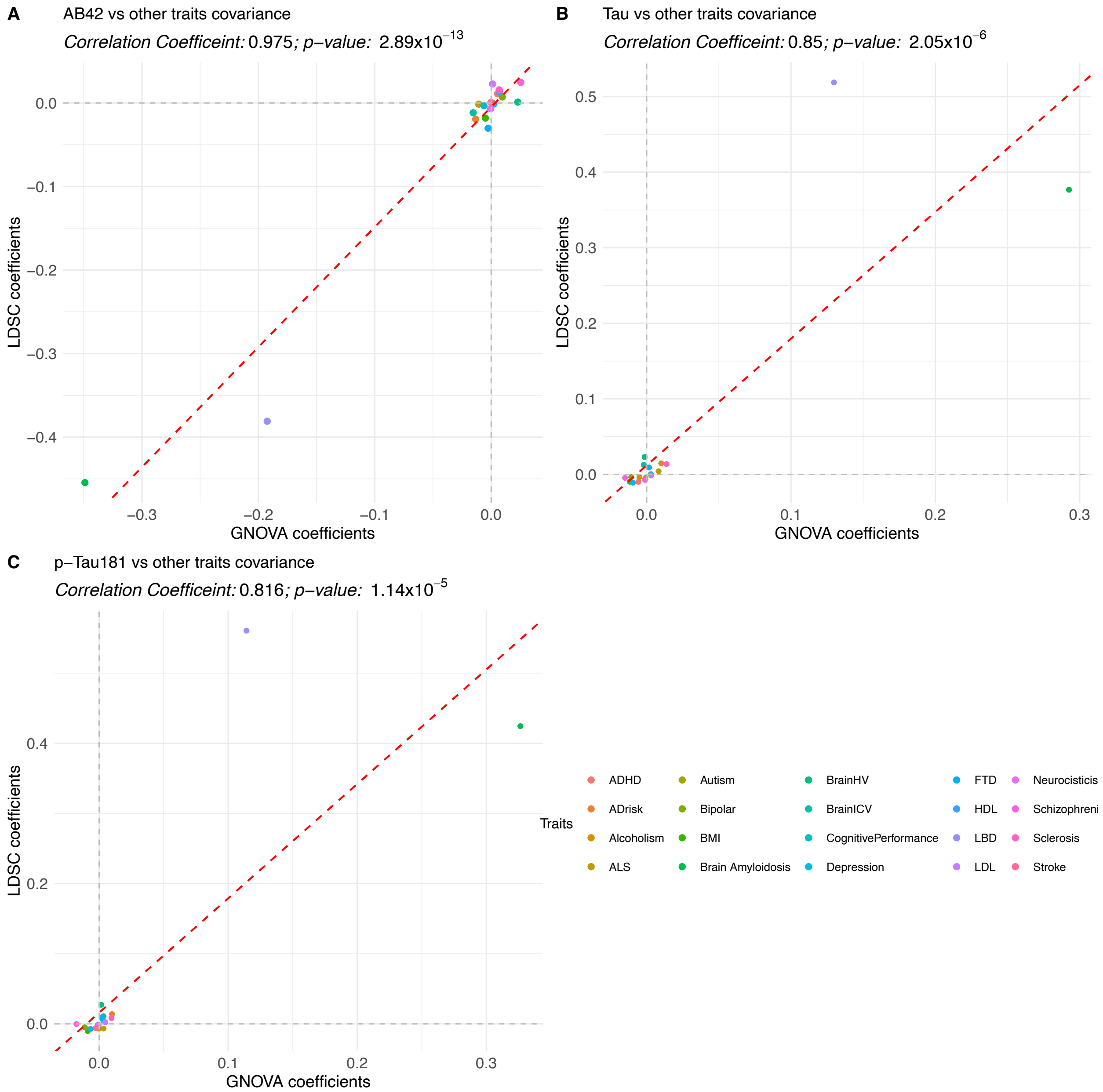

Supplementary Figure 17: *Scatterplot plot showing correlation between genetic covariance estimates from GNOVA and LDSC. Each dot represents a trait. X-axis shows estimates from GNOVA and Y-axis shows estimates from LDSC.* ***(A)*** *Correlation of covariance estimates between traits and Aβ42 (0.975; p:2.89×10^{-13}).* ***(B)*** *Correlation of covariance estimates between traits and t-tau (0.85; p:2.05×10^{-06}).* ***(C)*** *Correlation of covariance estimates between traits and Aβ42 (0.816; p:1.14×10^{-05}). Source data for the figure is provided in Supplementary Data 15.*

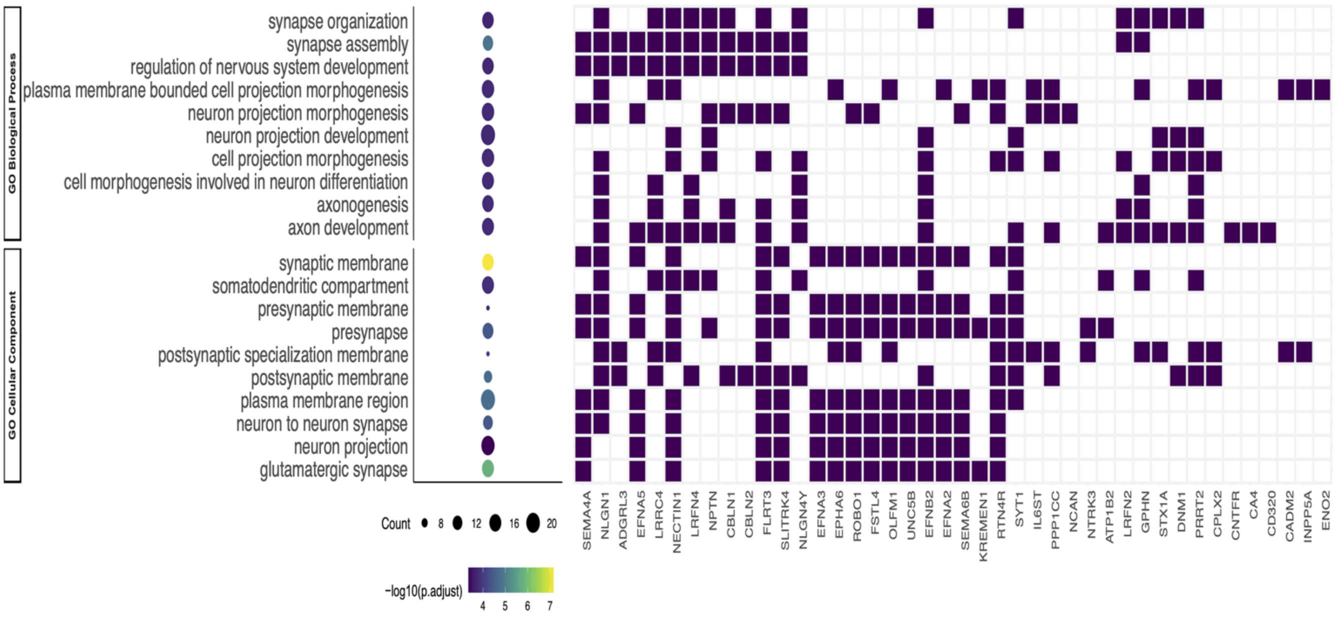

Supplementary Figure 18: *Pathway plot for all trans protein QTLs that colocalize with Chromosome 16 loci. The gene ids associated with these proteins were used for pathway enrichment. Y-axis shows top Gene Ontology (GO) terms that pass FDR <0.05 threshold, in descending order of significance. Color of dot plots show the FDR p value whereas sizes of dots represent the number of genes within the pathway. The corresponding heatmap shows the genes present within each path. Source data for the figure is provided in Supplementary Data 19.*

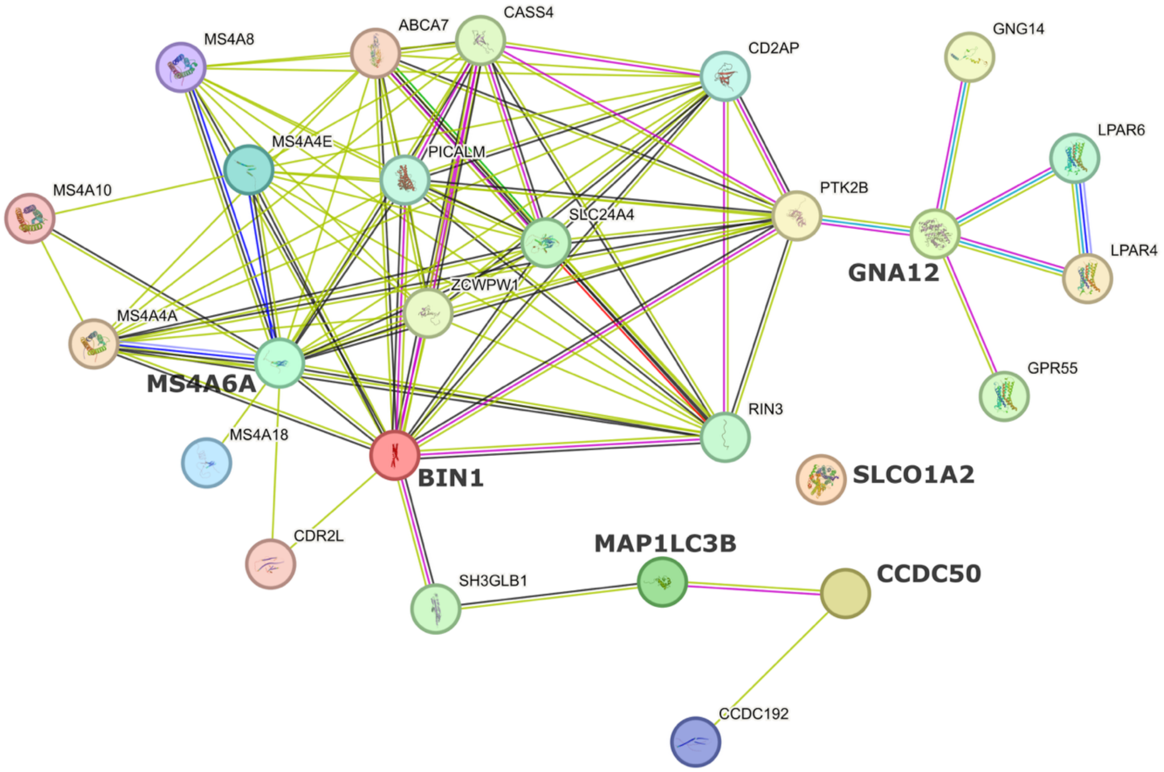

Supplementary Figure 19: *Protein-protein interaction plot showing interaction of protein products of genes nominated as functional in the significant loci identified in meta-analysis. Proteins highlighted bold are those that were nominated by our gene prioritization analysis. Source data for the figure are available in Main Table 2.*

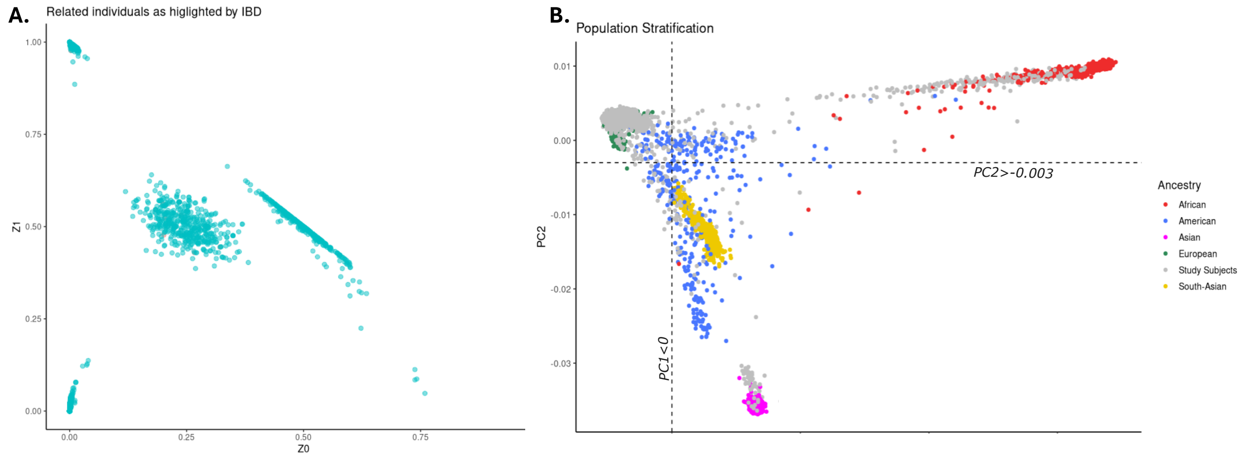

Supplementary Figure 20: (A) *Identity by descent (IBD) plot showing probability of sharing no allele (Z0; x-axis) and one allele (Z1; y-axis) in related individuals. "0" Z0 and Z1 value shows exact duplicates whereas "1" Z1 shows parent-offspring pairs. Only individuals with pihat <0.2 were kept.* ***(B)*** *Genetic principal component analysis (PCA) plot of study samples with 1000 Genomes samples as anchor. Only European ancestry population (PC1<0; PC2>-0.003) population were kept. Source data are provided as a Source Data file for this figure.*

Additional Material

1. Knight ADRC

Charles F. and Joanne Knight Alzheimer Disease Research Center (Knight ADRC), housed at Washington University in St. Louis, is one of 30 ADRCs funded by NIH. The goal of this collaborative research effort is to advance AD research with the goal of treatment or prevention of AD. The subjects included in this study are from the Memory and Aging Project (MAP) supported by Knight ADRC. As part of the project, subjects undergo annual psychometric testing and interviews along with biennial or triennial PET, MRI and CSF collection. Further details on Knight ADRC and MAP can be found at https://knightadrc.wustl.edu/

2. Alzheimer's Disease Neuroimaging Initiative (ADNI):

Data used in the analyses performed in this article were obtained from the Alzheimer's Disease Neuroimaging Initiative (ADNI) database (adni.loni.usc.edu). ADNI was launched in 2003 as a public-private partnership, led by Principal Investigator Michael W. Weiner, MD. The primary goal of ADNI has been to test whether serial magnetic resonance imaging (MRI), positron emission tomography (PET), other biological markers, and clinical and neuropsychological assessment can be combined to measure the progression of mild cognitive impairment (MCI) and early Alzheimer's disease (AD). For up-to-date information, see www.adni-info.org.

3. ADNI Department of Defense studies (ADNI-DOD):

The main objective of the ADNI Department of Defense studies (ADNI-DOD) study is to examine possible connections between Post Traumatic Stress Disorder (PTSD) and/or Traumatic Brain Injury (TBI) and the signs and symptoms of Alzheimer's disease (AD). This initiative is a branch of the larger Alzheimer's Disease Neuroimaging Initiative (ADNI). All participants undergo a comprehensive set of standardized assessments as part of the ADNI protocol. These assessments encompass medical and cognitive evaluations, lumbar puncture, blood tests, magnetic resonance imaging (MRI), and amyloid PET Florbetapir F18 scanning. Before undergoing MRI, lumbar puncture, or PET scans, individuals are queried about any existing medical conditions and their medication usage. For up-to-date information, see www.adni-info.org.

4. Australian Imaging, Biomarkers and Lifestyle (AIBL)

Australian Imaging, Biomarker & Lifestyle Flagship Study of Ageing (AIBL) study was initiated in November 2006 with a prospective longitudinal study design, intending to assess all participants at 18-month intervals[1]. Over 1100 participants, comprising of healthy controls (HC), individuals with mild cognitive impairment (MCI) and Alzheimer's disease (AD) were evaluated over a 4.5-year time span. Data collection was done in two centers with 40% subjects from Perth in Western Australia and 60% from Melbourne, Victoria. Neuroimaging was planned for 25% of each of these groups. However, the actual enrollment in the neuroimaging arm included 177 HC, 57 MCI, and 53 individuals with mild AD, constituting 26% of the entire cohort[2]. All individuals in the study were aged 60 or older, and they were in generally good health, with no prior history of stroke or any other neurological conditions. For those diagnosed with AD, they met the criteria outlined by the National Institute of Neurological and Communicative Disorders–Alzheimer's Disease and Related Disorders (NINCDS-ADRDA) for probable AD, as defined by McKhann et al in 19844. Additionally, these AD patients exhibited a Clinical Dementia Rating (CDR) of 1 or higher. For additional information see https://aibl.org.au/

5. Biomarkers for Older Controls at Risk for Dementia (BIOCARD)
The Biomarkers for Older Controls at Risk for Dementia (BIOCARD) study is a longitudinal study that was initiated in 1995 in the National Institute of Mental Health (NIMH) Intramural Research Program. The study encompassed clinical, neuropsychological, and neuropsychiatric evaluations, neuroimaging, and fluid biomarkers assessment to understand and predict progression from normal cognition to mild cognitive impairment (MCI) and dementia, particularly Alzheimer's disease (AD). Average age at enrollment was 55 years. The study was stopped in 2005, however in 2009 it was re-started at Johns Hopkins University (JHU). Between 1995 and 2005, clinical and cognitive assessment were performed annually and CSF, blood sampling, and magnetic resonance imaging (MRI) were done every 2 year[3]. From 2009 until 2015, participants been annually evaluated for clinical, cognitive assessments and blood sample collection with bi-annual MRI and CSF being restarted since 2015[3].

6. Barcelona-1
Barcelona -1 is a longitudinal observational study consisting of ~300 subjects at baseline carried out in the Memory and Disorder unit at the University Hospital Mutua de Terrassa, Terrassa, Barcelona, Spain. Cases include subjects diagnosed with AD dementia (ADD), non-AD dementias (non-ADD), mild cognitive impairment (MCI), or subjective memory complaints (SMC). Clinical information was collected at baseline as well as longitudinally and lumbar puncture (LP) and amyloid PET were performed if subjects had diagnosis of MCI, early-onset dementia (<65 years), or dementia with atypical clinical feature[4].

7. Dominantly Inherited Alzheimer Network (DIAN)
The Dominantly Inherited Alzheimer Network (DIAN), led by Washington University School of Medicine in St. Louis, is focused on the study of Autosomal Dominant AD (ADAD). It is a family-based long-term observational study with standardized clinical and cognitive testing, brain imaging, and biological fluid collection (blood, cerebrospinal fluid) from subjects with the intent of identifying changes in pre-symptomatic and symptomatic gene carriers who are expected to develop AD. Since the focus of this study is on ADAD, which has an early age of onset compared to sporadic AD, the subjects in this cohort are younger on average compared to other cohorts. The data used in this study are from data freeze 15 (DF15). Amyloid imaging data used were processed by the DIAN imaging core. Additional details on DIAN can be found at https://dian.wustl.edu/.

8. Saarland University in Homburg/Saar, Germany (HB)
CSF biomarker data from 107 subjects were collected at Saarland University in Germany. These included demented outpatients who referred to a hospital memory clinic between 1995 and 2001 for diagnostic evaluation[5]. For clinical diagnosis of probable AD, the National Institute of Neurological and Communication Disorders and Stroke/AD and Related Disorders Association criteria were applied[5]. Additional evaluation performed included CSF and blood sample collection, assessment of cognitive impairment, APOE genotyping among others. Age at onset was defined by the appearance of the first clinical symptoms[5]

9. Hospital Sant Pau
The memory unit of Hospital Sant Pau located in Barcelona, Spain, attends more than 2000 patients annually. Routine assessment includes neuropsychological evaluation, CSF and blood sample collection and MRI tests. Additional, PET examinations are conducted in select group of subjects. The subjects included in this study were recruited from the year 2009 to 2016[6]. Additional information on the study can be found at https://santpaumemoryunit.com/

10. London

The CSF biomarker data under the London cohort were from EDAR and DESCRIPA studies. EDAR is a prospective, longitudinal study aimed at examining and evaluating biomarkers of early AD[7,8]. In particular, the study is focused on Aβ oligomers and the effect of genetic variants on these oligomers. More information on the cohort can be found at http://www.edarstudy.eu. DESCRIPA study is also a prospective, multi-center study. Lead by the European AD Consortium, the focus of the study is on collecting data from non-demented subjects to develop screening and diagnostic criteria for AD[8]. Further details of this study can be found in Visser et. al, (2008)[9].

11. Parkinson's disease Movement disorder clinic (MARS)

The CSF biomarker data within the MARS cohort were received from movement disorder clinic at Washington university in St. Louis. The data used in this study comprised of Parkinson's disease cases and healthy controls, however the clinic has other studies focused on Dystonia, Tourette Syndrome or Tics, Huntington Disease, Tremor and other types of movement disorders. At study enrollment, participants complete a comprehensive motor, cognitive and clinical assessment. Longitudinal follow-up is every 1-3 years as long as patient is willing and able to participate[10].

12. Mayo

The CSF biomarker data under the Mayo cohort were collected by biospecimen accessioning and processing core at Mayo Clinic in Rochester, Minnesota. Research core at Mayo Clinic usually serve study groups affiliated with the clinic, however some facilities are available to investigators worldwide. The Proteomics Core in particular offer routine services to identify and quantify proteins. Longitudinal CSF samples were from available from 443 individuals comprising of close to 80% AD cases.

13. Clinic de Barcelona

CSF samples in this cohort were collected from individuals recruited at the Alzheimer's disease and other cognitive disorder unit, from the Hospital Clinic de Barcelona (Barcelona, Spain)[11]. CSF samples were collected from 256 individuals with 36.72% being male. In addition to CSF profiling, assessment of cognitive impairment was also performed as part of data collection. Samples were provided by Dr. Molinuevo.

14. National Alzheimer's Coordinating Center (NACC)

National Alzheimer's Coordinating Center (NACC) is the central data repository for all NIA's Alzheimer's Disease and Research Centers (ADRC) Program, including Mayo ADRC and Knight ADRC. The CSF biomarker data utilized from NACC in this paper were selected after proper IBD to make sure no duplicates or close relative of samples from other ADRC cohort were included in GWAS. Currently, NACC houses data from 47000 participants from across 33 ADRCs and 4 exploratory centers. The data is collected using a prospective, standardized, and longitudinal clinical evaluation of subjects. NACC protocol requires annual follow-up for as long as the participant is able to be involved and is thus longitudinal in nature. CSF as well Imaging data are available as a part of the data repository. However, the data collection schedule and subject enrollment protocol vary by center. For additional information please check this link https://naccdata.org/

15. Parkinson's Progression Markers Initiative (PPMI)

The Parkinson's Progression Markers Initiative (PPMI) is a research initiative designed to accelerate the development of biomarkers for Parkinson's disease (PD). PPMI was launched in 2010 and is sponsored by The Michael J. Fox Foundation for Parkinson's Research (MJFF).

PPMI is a longitudinal study with collaboration among multiple clinical sites and research center. Individuals who are newly diagnosed with PD, as well as those who are at risk for developing the disease (such as close relatives of PD patients) and healthy control subjects are eligible to enroll in the study. Data from imaging studies, blood and CSF samples as well as genetic information are available to eligible researchers. For more information on PPMI please visit https://www.ppmi-info.org/

16. Skåne University Hospital, Sweden (SWEDEN)
The samples included in this cohort were evaluated at memory disorder unit at Skåne University Hospital. Of the total 315 subjects included in this study, 100% of them were diagnosed as having AD. Details of sample collection in this cohort have been described previously[12]. Briefly, at the thorough physical, neurological, and psychiatric examination, as well as a clinical interview focusing on cognitive symptoms are performed as baseline visit. Furthermore, cognitive tests, analysis of APOE genotype, and imaging of the brain were also done. Additional information on the center can be found at https://vard.skane.se/en/skane-university-hospital/

17. Perelman School of Medicine at the University of Pennsylvania (UPENN)
The CSF biomarker data used in this study were received from the University of Pennsylvania's Alzheimer's Disease Research Center. Participants should be of age 55 years or older to be enrolled in the cohort. Annual visit includes cognitive testing, neurological exam, blood samples and interview. MRI and PET scan data is available for a subset of the samples. The longitudinal biomarker data used in this study were from 182 unique participants and were measured using Luminex platform.

18. University of Washington (UW)
The UW Alzheimer's Disease Research Center, located in Seattle, is part of a one of the NIH funded ADRCs focused on Alzheimer's disease and related dementia. The center conducts yearly visits during which participants are required to complete tests of their memory and thinking and have a brief physical and neurological examination. Blood draw is usually part of the annual visits but CSF collection is voluntary. For additional information on the center please visit https://depts.washington.edu/mbwc/adrc

19. Vanderbilt Memory and Aging Project (VMAP)
Founded in 2012, Vanderbilt Memory and Aging Project (VMAP) is a longitudinal study focused on study of brain aging. As part of the project, physical and frailty examination, fasting blood draw, neuropsychological assessment, echocardiogram, cardiac MRI, CSF collection and brain MRI are usually obtained at baseline visit. Follow-up visits are then scheduled at 18 months, 3 years, 5 years, and so on. To be a part of the study, individuals must be 50+ year old and show no signs of cognitive decline. More details on the project can be found at https://www.vumc.org/vmac/home

20. Wisconsin Alzheimer's Disease Research Center (Wisc ADRC)
The Wisconsin Alzheimer's Disease Research Center housed at University of Wisconsin-Madison is one of the several NIH funded ADRC centers in the USA. Established in 2009, the primary focus of the center is to improve early detection of Alzheimer's disease and finding ways to delay onset and progression. As of 2023, the center has collected data from 1075 core participants of which 601 have undergone CSF collection and 572 have PET scan available. Cognitive assessment is done either annually or at every other year visit. Further detail can be found at https://www.adrc.wisc.edu/

21. Ace Alzheimer Center Barcelona
Ace Alzheimer Center Barcelona also known as Fundació ACE (FACE) is a leading non-profit research and clinical center specializing in Alzheimer's disease and other neurodegenerative disorders[13]. Founded in 1995 and headquartered in Barcelona, this center combines patient clinical diagnosis, day care services, trials unit and research activities. Till date, the center has diagnosed over 30,000 patients, collected 23,000 blood and 2,800 cerebrospinal fluid samples, analyzed 13,000 genetic samples and participated in over 150 clinical trials during its existence4. For more details, visit http://www.fundacioace.com/en

22. ALFA study
The Alzheimer’s and Families (ALFA) project was launched in 2013 by the Barcelona βeta Brain Research[14]. Inclusion criteria was cognitively normal Spanish and/or Catalan-speaking individual between 45 and 74 years that agreed with the study procedures and tests which included interview to assess risk factors, cognitive tests, a blood sample collection, and MRI[15]. Currently the study has data from 2743 participants, 50% of whom are adult children of AD patients. A subset of samples are included in a nested ALFA+ project which is a long term longitudinal study[14]. CSF based biomarker levels and ATN classification are also available for these samples.

23 European Alzheimer’s and Dementia Biobank & H70 cohort
This consortium comprises 20,464 Alzheimer’s disease (AD) cases and 22,244 controls from 15 European countries (Belgium, Bulgaria, Czech Republic, Denmark, Finland, France, Germany, Greece, Italy, Portugal, Spain, Sweden, Switzerland, The Netherlands, and the UK), following rigorous quality control measures. Genotyping was performed at three independent centers located in France, Germany, and the Netherlands, resulting in the establishment of three nodes: EADB-France, EADB-Germany, and EADB-Netherlands. For the current analysis, we included all participants with cerebrospinal fluid (CSF) measurements from the EADB dataset and the H70 Birth Cohort Study. The participants with CSF biomarker data from the H70 cohort were assessed using standardized methods. Alzheimer’s disease diagnosis was based on the National Institute of Neurological and Communication Disorders and Stroke/AD and Related Disorders Association (NINCDS-ADRDA) criteria. All subjects included in the study had genotyping data, clinical dementia phenotypes, and detailed evaluations of neurobiological markers of dementia.
As part of the Gothenburg H70 Birth Cohort Study (n=471, mean age=74 years), Gothenburg, Sweden, and the Clinical AD samples (n=474, mean age=76) from Sweden, CSF biomarker data were measured using standardized methods described previously[16-19]. Alzheimer’s disease was diagnosed according to the National Institute of Neurological and Communication Disorders and Stroke/AD and Related Disorders Association criteria. Subjects have genotyping data, clinical phenotypes of dementia, and detailed assessment of neurobiological markers of dementia.

23. European Medical Information Framework for Alzheimer's Disease

The European Medical Information Framework for Alzheimer's disease (EMIF-AD) is a retrospective study which was established with the aim of accelerating the discovery of novel biomarkers for AD and its underlying pathophysiological mechanisms[20]. Samples used in this project are from the EMIF-AD Multimodal Biomarker Discovery study (EMIF-AD MBD). In total, data (MRI scan, proteomics, genomics, metabolomics) from 1221 participants (n=492 control, n=527 MCI, n=202 AD dementia) from 11 European cohorts were collected and harmonized[21]. Detail genotyping and CSF biomarker details are available in Hong et. al[21]. Additional detail on the EMIF project is available at https://www.emif.eu/.

24. European Prevention of Alzheimer's Dementia

The European Prevention of Alzheimer's Dementia (EPAD) Consortium is a European-based study whose aim is to facilitate a trials-ready population for the prevention of Alzheimer's dementia[22]. Volunteers were aged 50 or older at recruitment (between 2016 and 2020) without a dementia diagnosis (Clinical Dementia Rating scale score <1). Over 2,000 volunteers were recruited from 29 sites across 10 European countries, the majority of whom agreed to donate CSF and blood biosamples for analysis. Genotyping was performed on 2,000 samples via the Illumina Infinium Global Screening Array. After quality control (see Supplementary Table 22), data were available for 1,910 individuals with corresponding CSF and covariate data being present for 1,369 (Aβ42), 1,365 (Tau) and 1,364 (pTau).

25. MISSION-AD

MISSION-AD studies are two global phase 3 studies (E2609-G000-301 or MissionAD1; E2609-G000-302 or MissionAD2) to investigate the efficacy and safety of elenbecestat, an oral β-site APP-cleaving enzyme 1 (BACE-1) inhibitor, as a potential therapy for early AD[23]. The population consisted of subjects with a diagnosis of MCI due to AD and no more than 25% diagnosed as early-stage mild dementia due to AD. The studies terminated early in September 2019, due to an unfavorable benefit-risk profile of elenbecestat and at a time when the development of other BACE inhibitors had also been stopped for several reasons including lack of benefit. At the time of the early termination, the MISSION-AD studies had recruited 2212 randomized subjects from a cohort of 9758 screened subjects. The screening process was performed in 5 sequential tiers, with an amyloid PET scan or a CSF sample taken at tier 5 to determine baseline amyloid burden status of study subjects. This GWAS meta-analysis includes 462 randomized and screen failure subjects from MISSION-AD who have consented to exploratory research and have both CSF samples where biomarkers have been measured and DNA genotyping data available.

26. Janssen Cohort

Janssen cohort included data from two study population: One treated with bapineuzumab and other with BACE1 inhibitor[24-26]. Within the Bapineuzumab cohort, Amyloid PET imaging, CSF biomarkers, or MRI were assessed[25]. Samples within BACE1 cohort were pooled from three separate studies[26]. Additional information about these study population has been presented previously by Salloway et. al. (2014)[25], Liu et. al. (2018)[24] and Li. et. al. (2023)[26].

Note: Samples included in Blennow and Zetter cohorts were provided by Dr. Blennow and Dr. Zetterberg respectively. DNA samples along with CSF biomarker measurement, disease status, gender and age at sample collection information were provided. Additional information about these cohorts is not available at this time.

References:

1. C F, SR R-S, S B, et al. Fifteen Years of the Australian Imaging, Biomarkers and Lifestyle (AIBL) Study: Progress and Observations from 2,359 Older Adults Spanning the Spectrum from Cognitive Normality to Alzheimer's Disease - PubMed. *Journal of Alzheimer's disease reports*. 06/03/2021;5(1)doi:10.3233/ADR-210005
2. CC R, KA E, M R, et al. Amyloid imaging results from the Australian Imaging, Biomarkers and Lifestyle (AIBL) study of aging - PubMed. *Neurobiology of aging*. 2010 Aug;31(8)doi:10.1016/j.neurobiolaging.2010.04.007
3. Koychev I, Vaci N, Bilgel M, et al. Prediction of rapid amyloid and phosphorylated-Tau accumulation in cognitively healthy individuals. *Alzheimer's & Dementia: Diagnosis, Assessment & Disease Monitoring*. 2020/01/01;12(1)doi:10.1002/dad2.12019
4. I Á, M D-F, M A, et al. Added value of cerebrospinal fluid multimarker analysis in diagnosis and progression of dementia - PubMed. *European journal of neurology*. 2021 Apr;28(4)doi:10.1111/ene.14658
5. M R, S W, J D, et al. Tau and Abeta42 protein in CSF of patients with frontotemporal degeneration - PubMed. *Neurology*. 06/11/2002;58(11)doi:10.1212/wnl.58.11.1622
6. I S, I I-G, D A, et al. Diagnostic and Prognostic Value of the Combination of Two Measures of Verbal Memory in Mild Cognitive Impairment due to Alzheimer's Disease - PubMed. *Journal of Alzheimer's disease : JAD*. 2017;58(3)doi:10.3233/JAD-170073
7. Barnett JH, Blackwell A, Scheltens P, et al. O3-02-03: Cognitive function and cognitive change in dementia, mild cognitive impairment, and healthy aging: The EDAR study. *Alzheimer's & Dementia*. 6doi:10.1016/j.jalz.2010.05.399
8. N V, H P, A F, et al. Genetic Risk as a Marker of Amyloid-β and Tau Burden in Cerebrospinal Fluid - PubMed. *Journal of Alzheimer's disease : JAD*. 2017;55(4)doi:10.3233/JAD-160707
9. Visser P, Verhey F, Boada M, et al. Development of Screening Guidelines and Clinical Criteria for Predementia Alzheimer's Disease: The DESCRIPA Study. *Neuroepidemiology*. 2008 Jun 2;30(4)doi:10.1159/000135644
10. MC C, PS M, AJ W, et al. Parkinson disease clinical subtypes: key features & clinical milestones - PubMed. *Annals of clinical and translational neurology*. 2020 Aug;7(8)doi:10.1002/acn3.51102
11. Falcon C, Monté-Rubio GC, Grau-Rivera O, et al. CSF glial biomarkers YKL40 and sTREM2 are associated with longitudinal volume and diffusivity changes in cognitively unimpaired individuals. *NeuroImage: Clinical*. 2019/01/01;23doi:10.1016/j.nicl.2019.101801
12. J H, L M, H Z, E V, K B, O H. Evaluation of CSF biomarkers as predictors of Alzheimer's disease: a clinical follow-up study of 4.7 years - PubMed. *Journal of Alzheimer's disease : JAD*. 2010;21(4)doi:10.3233/jad-2010-100207
13. M B, L T, I H, et al. Design of a comprehensive Alzheimer's disease clinic and research center in Spain to meet critical patient and family needs - PubMed. *Alzheimer's & dementia : the journal of the Alzheimer's Association*. 2014 May;10(3)doi:10.1016/j.jalz.2013.03.006
14. N V-T, P G, B R-F, et al. Genetic characterization of the ALFA study: Uncovering genetic profiles in the Alzheimer's continuum - PubMed. *Alzheimer's & dementia : the journal of the Alzheimer's Association*. 2024 Mar;20(3)doi:10.1002/alz.13537

15. JL M, N G, JD G, et al. The ALFA project: A research platform to identify early pathophysiological features of Alzheimer's disease - PubMed. *Alzheimer's & dementia (New York, N Y)*. 03/03/2016;2(2)doi:10.1016/j.trci.2016.02.003
16. T RS, F A, K B, et al. The Gothenburg H70 Birth cohort study 2014-16: design, methods and study population - PubMed. *European journal of epidemiology*. 2019 Feb;34(2)doi:10.1007/s10654-018-0459-8
17. K B, A W, H A, C S, J S, E V. Tau protein in cerebrospinal fluid: a biochemical marker for axonal degeneration in Alzheimer disease? - PubMed. *Molecular and chemical neuropathology*. 1995 Dec;26(3)doi:10.1007/BF02815140
18. E V, H V, P D, et al. Quantification of tau phosphorylated at threonine 181 in human cerebrospinal fluid: a sandwich ELISA with a synthetic phosphopeptide for standardization - PubMed. *Neuroscience letters*. 05/05/2000;285(1)doi:10.1016/s0304-3940(00)01036-3
19. N A, C H, P D, et al. Cerebrospinal fluid beta-amyloid(1-42) in Alzheimer disease: differences between early- and late-onset Alzheimer disease and stability during the course of disease - PubMed. *Archives of neurology*. 1999 Jun;56(6)doi:10.1001/archneur.56.6.673
20. Bos I, Vos S, Vandenberghe R, et al. The EMIF-AD Multimodal Biomarker Discovery study: design, methods and cohort characteristics. *Alzheimer's Research & Therapy 2018 10:1*. 2018-07-06;10(1)doi:10.1186/s13195-018-0396-5
21. S H, V D, O O, et al. TMEM106B and CPOX are genetic determinants of cerebrospinal fluid Alzheimer's disease biomarker levels - PubMed. *Alzheimer's & dementia : the journal of the Alzheimer's Association*. 2021 Oct;17(10)doi:10.1002/alz.12330
22. Saunders S, Gregory S, Clement MHS, Birck C, Geyten Svd, Ritchie CW. The European Prevention of Alzheimer's Dementia Programme: An Innovative Medicines Initiative-funded partnership to facilitate secondary prevention of Alzheimer's disease dementia. *Frontiers in Neurology*. 2022 Nov 22;13doi:10.3389/fneur.2022.1051543
23. C R, J K, M G, S K, M K. Amyloid and APOE Status of Screened Subjects in the Elenbecestat MissionAD Phase 3 Program - PubMed. *The journal of prevention of Alzheimer's disease*. 2021;8(2)doi:10.14283/jpad.2021.4
24. E L, D W, R S, et al. Biomarker pattern of ARIA-E participants in phase 3 randomized clinical trials with bapineuzumab - PubMed. *Neurology*. 03/06/2018;90(10)doi:10.1212/WNL.0000000000005060
25. S S, R S, NC F, et al. Two phase 3 trials of bapineuzumab in mild-to-moderate Alzheimer's disease - PubMed. *The New England journal of medicine*. 01/23/2014;370(4)doi:10.1056/NEJMoa1304839
26. QS L, S F, J S, J T, G R, GP N. Genome-wide association study of abnormal elevation of ALT in patients exposed to atabecestat - PubMed. *BMC genomics*. 09/01/2023;24(1)doi:10.1186/s12864-023-09625-6
