# Supplementary material for: GWAS meta-analysis of cerebrospinal fluid Alzheimer’s biomarkers reveals loci regulating lipids, brain volume and autophagy
Source: Nat Commun. 2026 Apr 21;17:7385. doi: 10.1038/s41467-026-71682-8 (PMC13402690; doi:10.1038/s41467-026-71682-8)
Supplement: Supplementary file 3 — Description of Additional Supplementary Files [file 41467_2026_71682_MOESM3_ESM.pdf]

Description of Additional Supplementary Files

File name: Supplementary Data 1
Description: Table showing summary statistics of signals significant in European metanalysis in non-European GWAS and previous GWAS for the phenotype; p-values are unadjusted p values from GWAS

File name: Supplementary Data 2
Description: Table showing summary statistics of variants identified in previous GWAS for the phenotypes of interest in current European meta-analysis; p-values are unadjusted p values from GWAS

File name: Supplementary Data 3
Description: Table showing signal identified as significant after pleiotropy adjustment using MTAG and pleioFDR; MTAG and METAL p-values are unadjusted p values where as pleioFDR p value are adjusted for multiple comparisons.

File name: Supplementary Data 4
Description: Colocalization results of MTAG signals with Brain eQTL and cis pQTL resources that had PP.H4>0.8

File name: Supplementary Data 5
Description: Table showing effect size for variants from meta-analysis in AD and CO stratified analysis, "pvalues" are unadjusted p-values from GWAS

File name: Supplementary Data 6
Description: Table showing Z statistics and p-value for each endophenotype and disease diagnosis GWAS

File name: Supplementary Data 7
Description: Gene prioritizations score weighting methodology

File name: Supplementary Data 8
Description: Gene prioritization score for all genes tested

File name: Supplementary Data 9
Description: Colocalization results of top signals with Brain eQTL and cis pQTL resources

File name: Supplementary Data 10
Description: Annotation results of all variants in LD of lead variants in Aβ42 GWAS from FUMA ANNOVAR

File name: Supplementary Data 11
Description: Annotation results of all variants in LD with lead variants in t-tau GWAS from FUMA ANNOVAR

File name: Supplementary Data 12
Description: Annotation results of all variants in LD with lead variants in p-tau181 GWAS from FUMA ANNOVAR

File name: Supplementary Data 13
Description: Trans Colocalization results of top chromosome 16 variant with CSF pQTL resources

File name: Supplementary Data 14
Description: Colocalization results of top variants with AD risk GWAS

File name: Supplementary Data 15
Description: Genetic Covariance Results from GNOVA (with and without APOE gene region); p-value column shows unadjusted p-values and FDR p-value column shows False discovery rate adjusted p-values.

File name: Supplementary Data 16
Description: Table showing Mendelian Randomization (MR) estimates for the exposures on risk of AD; “p-val” column shows unadjusted p-value.

File name: Supplementary Data 17
Description: GWAS Catalogue results of top variants associated with the phenotypes; “P-VALUE” column shows unadjusted p-value from source summary statistics.

File name: Supplementary Data 18
Description: Pathway and disease ontology results of genes associated with phenotypes; “pvalue” is the unadjusted p-value.

File name: Supplementary Data 19
Description: Pathway results of trans pQTL genes colocalizing with chromosome 16 GWAS loci; “pvalue” is the unadjusted p-value.

File name: Supplementary Data 20
Description: Demographic information of individual studies included in EADB cohort

File name: Supplementary Data 21
Description: Demographic information of non-European ancestry individual

File name: Supplementary Data 22
Description: Sample Genotype quality control and analysis detail in each cohort
