# Supplementary material for: GWAS meta-analysis of cerebrospinal fluid Alzheimer’s biomarkers reveals loci regulating lipids, brain volume and autophagy
Source: Nat Commun. 2026 Apr 21;17:7385. doi: 10.1038/s41467-026-71682-8 (PMC13402690; doi:10.1038/s41467-026-71682-8)
Supplement: Supplementary file 26 — Reporting Summary [file 41467_2026_71682_MOESM26_ESM.pdf]

nature portfolio

Corresponding author(s): Carlos Cruchaga

Last updated by author(s): Dec 1, 2025

Reporting Summary

Nature Portfolio wishes to improve the reproducibility of the work that we publish. This form provides structure for consistency and transparency in reporting. For further information on Nature Portfolio policies, see our Editorial Policies and the Editorial Policy Checklist.

Statistics

For all statistical analyses, confirm that the following items are present in the figure legend, table legend, main text, or Methods section.

n/a	Confirmed	
☐	☒	The exact sample size (*n*) for each experimental group/condition, given as a discrete number and unit of measurement
☐	☒	A statement on whether measurements were taken from distinct samples or whether the same sample was measured repeatedly
☐	☒	The statistical test(s) used AND whether they are one- or two-sided<br>*Only common tests should be described solely by name; describe more complex techniques in the Methods section.*
☐	☒	A description of all covariates tested
☐	☒	A description of any assumptions or corrections, such as tests of normality and adjustment for multiple comparisons
☐	☒	A full description of the statistical parameters including central tendency (e.g. means) or other basic estimates (e.g. regression coefficient) AND variation (e.g. standard deviation) or associated estimates of uncertainty (e.g. confidence intervals)
☐	☒	For null hypothesis testing, the test statistic (e.g. *F*, *t*, *r*) with confidence intervals, effect sizes, degrees of freedom and *P* value noted<br>*Give P values as exact values whenever suitable.*
☐	☒	For Bayesian analysis, information on the choice of priors and Markov chain Monte Carlo settings
☒	☐	For hierarchical and complex designs, identification of the appropriate level for tests and full reporting of outcomes
☒	☐	Estimates of effect sizes (e.g. Cohen's *d*, Pearson's *r*), indicating how they were calculated

Our web collection on statistics for biologists contains articles on many of the points above.

Software and code

Policy information about availability of computer code

Data collection	No software was used for data collection in this study.
Data analysis	All data analysis was performed using available software. GWAS was performed using PLINK v2.0 and meta-analysis was performed using METAL v1. Plots were derived in R using the packages "qqman (v0.1.9)", "karyoploteR (v1.30.0)", and "meta (v7.0)", and local plots were generated using LocusZoom (v1.40). GCTA-COJO was used to perform conditional analysis. Variant annotation and gene-based analysis was performed using FUMA. Colocalization was performed using the 'coloc (v5.2.3)' R package. The PheWAS analysis was performed using the 'gwasrapidd (v0.99.17)' R package. To calculate genetic correlation/covariance, GNOVA was used. Pathway analysis was performed using the 'ReactomePA (v1.48.0)' R package, while protein-protein interactions were identified using STRING-db.

For manuscripts utilizing custom algorithms or software that are central to the research but not yet described in published literature, software must be made available to editors and reviewers. We strongly encourage code deposition in a community repository (e.g. GitHub). See the Nature Portfolio guidelines for submitting code & software for further information.

Data

Policy information about availability of data

All manuscripts must include a data availability statement. This statement should provide the following information, where applicable:

- Accession codes, unique identifiers, or web links for publicly available datasets
- A description of any restrictions on data availability
- For clinical datasets or third party data, please ensure that the statement adheres to our policy

The GWAS meta-analysis summary statistics generated in this study have been deposited in the NIAGADS database under accession code NG00191. The files are also available to download from Washington University in St. Louis NeuroGenomics and Informatics Center web portal (https://neurogenomics.wustl.edu/open-science/raw-data/). The raw data from ACE, ALFA+, EADB, EPAD, EMIF, MISSION-AD and Janssen are protected and are not available due to data privacy laws. The processed summary statistics are available to qualified researchers upon request. Please contact individual study for their respective summary statistics access: ACE (http://www.fundacioace.com/en), ALFA+ (https://www.barcelonabeta.org/en/research/alfa), EADB (s.j.vanderlee@amstrdamumc.nl), EPAD (https://ep-ad.org/index.php/open-source-data/), EMIF-AD(https://emif-catalogue.eu), MISSION-AD(Mike_Nagle@eisai.com) and Janssen (qingqin.li@chdifoundation.org).

Research involving human participants, their data, or biological material

Policy information about studies with human participants or human data. See also policy information about sex, gender (identity/presentation), and sexual orientation and race, ethnicity and racism.

Reporting on sex and gender	Sex was not used as a filtering criteria for inclusion in the study. All sexes were used, and sex was included as a covariate in the GWAS models.
Reporting on race, ethnicity, or other socially relevant groupings	Because we are using genomic data, ancestry was used instead of race or ethnicity. Ancestry for each individual was determined based on principal components analysis using the 1000 Genome Project samples as population reference. While we focused on individuals of European ancestry to prevent confounding due to population stratification, secondary analyses were performed using data from individuals of other ancestry groups.
Population characteristics	Because these analyses were largely focused on measurements related to neurodegenerative disease, there is generally a bias towards older and less healthy individuals in many of the cohorts used. Age was used as a covariate in the GWAS models.
Recruitment	Participant recruitment varied by site and cohort. Most cohorts are hospital-derived, so participants are recruited based on presentation to the hospital, frequently due to signs of neurodegenerative disease.
Ethics oversight	Institutional Review Board of Washington University School of Medicine in St. Louis approved this study.

Note that full information on the approval of the study protocol must also be provided in the manuscript.

Field-specific reporting

Please select the one below that is the best fit for your research. If you are not sure, read the appropriate sections before making your selection.

☒ Life sciences ☐ Behavioural & social sciences ☐ Ecological, evolutionary & environmental sciences

For a reference copy of the document with all sections, see nature.com/documents/nr-reporting-summary-flat.pdf

Life sciences study design

All studies must disclose on these points even when the disclosure is negative.

Sample size	Sample size was determined based on maximization of the available data relevant to the phenotypes of interest. Sample sizes in this study are greater than previous analyses, where significant associations were already identified; we expected the increased sample sizes would improve our ability to detect significant associations.
Data exclusions	Individuals with outlier CSF measurements (defined as those outside of quartile 1 (Q1)-1.5*IQR or Q3+1.5*IQR) were removed. Due to issues potentially introduced during GWAS analysis caused by relatedness, individuals were filtered based on identity-by-descent analysis. The main analysis was performed on individuals of European ancestry, determined by principal components analysis.
Replication	Because this is the largest sample size to date for these traits, replication is not simple; however, each GWAS hit was tested for association with relevant disease and imaging phenotypes to ensure their robustness across multiple phenotypes. Of the 20 GWAS hits identified, 17 were associated with at least one of AD risk, AD progression, or amyloid imaging.
Randomization	Our genome-wide association studies test for the effects of alleles, which have been randomized in the population. All association tests were controlled by age, sex, principal components to account for population stratification, cohort, and genotyping method.
Blinding	As all outcomes are quantitative traits and inputs are genotypes, no observational bias can occur and therefore blinding is not relevant. This is not a randomized trial.

Reporting for specific materials, systems and methods

We require information from authors about some types of materials, experimental systems and methods used in many studies. Here, indicate whether each material, system or method listed is relevant to your study. If you are not sure if a list item applies to your research, read the appropriate section before selecting a response.

Materials & experimental systems

n/a	Involved in the study
☒	☐ Antibodies
☒	☐ Eukaryotic cell lines
☒	☐ Palaeontology and archaeology
☒	☐ Animals and other organisms
☒	☐ Clinical data
☒	☐ Dual use research of concern
☒	☐ Plants

Methods

n/a	Involved in the study
☒	☐ ChIP-seq
☒	☐ Flow cytometry
☒	☐ MRI-based neuroimaging

Plants

Seed stocks	NA
Novel plant genotypes	NA
Authentication	NA
