# Supplementary material for: GWAS meta-analysis of cerebrospinal fluid Alzheimer’s biomarkers reveals loci regulating lipids, brain volume and autophagy
Source: Nat Commun. 2026 Apr 21;17:7385. doi: 10.1038/s41467-026-71682-8 (PMC13402690; doi:10.1038/s41467-026-71682-8)
Supplement: Supplementary file 2 — Peer Review File [file 41467_2026_71682_MOESM2_ESM.pdf]

nature portfolio

Peer Review File

GWAS meta-analysis of Cerebrospinal fluid Alzheimer's biomarkers reveal loci regulating lipids, brain volume and autophagy

Corresponding Author: Dr Carlos Cruchaga

This file contains all reviewer reports in order by version, followed by all author rebuttals in order by version.

Version 0:

Reviewer comments:

Reviewer #1

(Remarks to the Author)
This is a large association study that investigates CSF biomarkers. The team pulled together almost 19000 individuals, which enables a deep examination of CSF biomarkers. The group examined associations that were both genetic and phenotypic, looking at AD risk, AD progression, endophenotypes including levels of A , pTau, and 63 traits including brain volume and white matter microstructure. They saw the massive APOE signal, well known for AD. The group identifies many of the classic biomarkers, such as BIN1 and CR1/2; they also observed signals for MSA4, that is linked to sTREM2. Each of these genes have been associated with AD phenotypes in many other studies. Because of the depth of the study, the group was able to identify some novel biomarkers not seen before. In particular, they identified MAP1LC3B and CCDC50. The discovery of MAP1LC3B as a biomarker is striking because this is one of the major proteins mediating autophagy, so it points to the strong role played by autophagy in the pathophysiology of AD. CCDC50 is a protein that has multiple ubiquitination sites, interacts with LC3B and is likely degraded via autophagy, although its function more likely lies in the NFKB/IFN response to dsRNA (released in AD by degenerating mitochondria). CCDC50 has been shown previously to interact with pTau and exhibit a tendency to aggregate. The direct link between autophagy and AD represents a finding that is significant and therefore will be of broad interest to the research community.

Reviewer #2

(Remarks to the Author)
Important study, comments below ...
Please include measures of precision with all effect estimates and P-values.
In the cross ancestry comparison, the comparisons have issues with presentation, not really any such thing as nominal or suggested significance, either its significant after multi-test correction or not. Can't be nominally or suggestively dead ;-) worth deleting or revising.
Additionally, if P is > 0.05 after correction, effect estimates cannot be accurately quantified in terms of directionality so I'd suggest removing that part too.
Please include lambda and LD intercepts in QC section.
Was phenotype included in analyses as covariate for sensitivity or stratification and comparison via I2 for hits at least?
All fixed effect meta-analyses should include I2 or other heterogeneity metrics as standard practice please.
Code, data availability and other FAIR / transparency related information is missing.
... nice work.
-

Reviewer #3

(Remarks to the Author)
I must say this is a really nice paper. The content is important and timely, and the presentation is excellent. Rather unusually for me, I have no negative comments, which reflects the high level of science presented in this manuscript. Publish as is.

Reviewer #4

(Remarks to the Author)
Timsina et al., perform GWAS meta-analysis of CSF biomarkers with 18,948 European individuals and identified 8 novel loci and 4 previously reported loci. This type of study is extremely important because clinical AD GWAS may be biased due to the inclusion of individuals who may later be diagnosed with AD, but here the authors use biological measurements from CSF that are less susceptible to these confounds. GWAS was conducted across multiple cohorts using standard approaches. The author found links between CSF biomarker loci and previous GWAS for AD risk, disease progression, and/or brain amyloidosis. The study also integrated multiple resources to provide functional context for the associations, including GO enrichment analysis and QTL colocalization. This is a highly useful resource that will be broadly valuable to the genetics and AD community. There are several technical points that I think could be improved, and some additional conceptual questions that these data may be helpful to address:
Technical points:
Meta-analysis of the summary statistics across the endophenotypes using METAL is non-standard and does not account for the same samples being used multiple times and the measurements being correlated. Many options exist for such an analysis of pleiotropic effects. Consider using min-P methods, MOSTest, pleiotropic association test, or MTAG: https://www.nature.com/articles/s41467-020-17368-1, https://journals.plos.org/plosgenetics/article?id=10.1371/journal.pgen.1010447.
The genetic correlations with AD risk are surprisingly low values but are significant, for example CSF A42 with AD risk rg=-0.013, pFDR=2.6710-38. Could the authors comment on this, validate with additional genetic correlation approaches, or check for typos.
Please perform a stratified analysis with AD or control groups and SNP*diagnosis interaction to determine if genetic effects are impacted by diagnostic status. The authors found variants not only associated with CSF level of the three biomarkers but also showed significant association with AD risk, disease progression and/or brain amyloidosis. Does this also occur if the data are stratified by AD status or just reflecting the relationship between biomarker measurements and AD status?
Could the author show the statistics of variants identified from previous studies, even if those are not significant in the current study.
Could the author show similar forest plots like Supplementary Figures 1-7 for non-European ancestries.
Although the author acknowledged “Finally, future research to evaluate genetic correlations between A42 and t-tau/p-tau181 and identify CSF A42-independent genetic signals associated with CSF t-tau /p-tau181 will be needed.”, please estimate the phenotypic and genetic correlations of these CSF biomarkers using this dataset.
Uncorrected P-value < 0.05 for pathway enrichment analysis may be too lenient. Some reported “significant” pathways have only one gene overlap, which are often difficult to believe. Please add gene counts in the pathway DB.
Conceptual questions that could be addressed:
Because the authors have separately measured Abeta and Tau CSF biomarkers, it seems possible to use Mendelian Randomization to address some important causal questions in the field. That is does Abeta pathology lead to Tau pathology? Similarly, does Abeta pathology causally contribute to AD risk? Does Tau pathology causally contribute to AD risk? Given the strong debates in the field currently, these MR analyses seem highly relevant.
It is commonly thought that effect sizes for endophenotypes are higher than disease diagnosis. Please compare effect size distributions for these endophenotypes versus AD diseases status, using for example this technique: https://www.nature.com/articles/s41588-018-0193-x.
The chr17 inversion locus contains MAPT, and some previous evidence shows that it does effect MAPT expression, but interestingly the locus does not show up in the GWAS for CSF tau measurements. I wonder if the authors could comment on this, and show the locus to see if it has some nominal level of significance for tau phenotypes.
Minor
Cohorts in Supplementary Table 16 look different from cohorts with summary statistics. Is this correct? And the cohorts are not always imputed to TOPMed GRCh38 as described in the methods. Please clarify.
How were ancestries defined? Please provide a threshold
Line 408: p=5.5247e-16 -> use the same format as other stats; there are also something like 10-08 or 10-8 (number varies)
Line 737: RCh38 -> GRCh38
Line 746 and Supplemental Table 16: MAF of 0.05 % looks too low, is that 5% (=0.05) or indeed 0.0005?
Line 782: toGRCh 37 -> to GRCh37
Check reference to Table S2 in Study description in Additional Material
European Prevention of Alzheimer's Dementia

Version 1:

Reviewer comments:

Reviewer #2

(Remarks to the Author)
Nice effort.

Two quick comments:
1. The GitHub that has the code for this should be provided for FAIR reasons. Not just links to software. This is a pretty comment requirement now.
2. The stratified data in Supp table 5 doesn't make sense to me, maybe I need another coffee. P-values seem inconsistent

with the meta if you just look at CO versus AD. For example, rs6733839 in this table looks impossible. Was this on the full set of results. p = 8.95E-01 meta-analyzed with p = 3.08E-02 should not generally give you a meta p of 5.24E-09 if that includes all the samples in the two subsets. This is confusing and should be clarified.

Good luck.

Reviewer #4

(Remarks to the Author)
The authors addressed all concerns in detail. I strongly support publication. Congratulations on an extremely interesting manuscript.

Open Access This Peer Review File is licensed under a Creative Commons Attribution 4.0 International License, which permits use, sharing, adaptation, distribution and reproduction in any medium or format, as long as you give appropriate credit to the original author(s) and the source, provide a link to the Creative Commons license, and indicate if changes were made.
In cases where reviewers are anonymous, credit should be given to 'Anonymous Referee' and the source.
The images or other third party material in this Peer Review File are included in the article's Creative Commons license, unless indicated otherwise in a credit line to the material. If material is not included in the article's Creative Commons license and your intended use is not permitted by statutory regulation or exceeds the permitted use, you will need to obtain permission directly from the copyright holder.
To view a copy of this license, visit https://creativecommons.org/licenses/by/4.0/

Reviewer comments responses
Thank you to the reviewers for the insightful comments and feedback in bold below. We have added additional clarifications and analyses requested by the reviewers and the detailed responses are below for each point.

Reviewer 1 comments
This is a large association study that investigates CSF biomarkers. The team pulled together almost 19000 individuals, which enables a deep examination of CSF biomarkers. The group examined associations that were both genetic and phenotypic, looking at AD risk, AD progression, endophenotypes including levels of Ab, pTau, and 63 traits including brain volume and white matter microstructure. They saw the massive APOE signal, well known for AD. The group identifies many of the classic biomarkers, such as BIN1 and CR1/2; they also observed signals for MSA4, that is linked to sTREM2. Each of these genes have been associated with AD phenotypes in many other studies. Because of the depth of the study, the group was able to identify some novel biomarkers not seen before. In particular, they identified MAP1LC3B and CCDC50. The discovery of MAP1LC3B as a biomarker is striking because this is one of the major proteins mediating autophagy, so it points to the strong role played by autophagy in the pathophysiology of AD. CCDC50 is a protein that has multiple ubiquitination sites, interacts with LC3B and is likely degraded via autophagy, although its function more likely lies in the NFKB/IFN response to dsRNA (released in AD by degenerating mitochondria). CCDC50 has been shown previously to interact with pTau and exhibit a tendency to aggregate. The direct link between autophagy and AD represents a finding that is significant and therefore will be of broad interest to the research community.
We thank this reviewer for his feedback and nice summary of the manuscript.

Reviewer 2 comments

1. **Please include measures of precision with all effect estimates and P-values.**

Response: We thank the reviewer for highlighting additional statistical measures that may be of interest to readers. As such, we have now added measures of precision along with estimates and p-values wherever applicable.

We have made detailed changes in the manuscript, some examples of which include:

Page 8, line 14: The variant rs6733839[T] in the *BIN1* locus was significantly associated with CSF Aβ42 (Effect=-0.06, SE=0.011, p=8.29×10^{-09}, I^2=0; Table 2; Supplementary Fig. 1).

Page 8, line 17: This variant was also found to influence t-tau and p-tau181 levels in the meta-analysis with consistent direction of effects as in previous studies ($Effect_{t\text{-}tau}$=0.06, $SE_{t\text{-}tau}$=0.010, $p_{t\text{-}tau}$=5.24×10^{-09}, $I^2_{t\text{-}tau}$=48.1; $Effect_{ptau181}$=0.073, $SE_{p\text{-}tau181}$=0.010, $p_{ptau181}$=2.28×10^{-12}, I^2=10.8; Table 2; Supplementary Fig. 2,5; Supplementary Table 1).

Page 15, line 19: Variants near *CR1* associated with Aβ42 was also significantly associated with increased AD risk (OR=1.127, CI=1.104-1.150, p=4.69×10^{-31}; Table 2) and brain amyloidosis (Effect= 0.097, SE= 0.016, p=4.75×10^{-31}; Table 2). Similarly, the lead GWAS variant on chromosome 2 near *BIN1* was also associated with increased AD risk (OR=1.184, CI=1.164-1.203, p=6.48×10^{-90}; Table 2) as well as accelerated AD progression (Effect=1.18×10^{-04}; SE= 4.07×10^{-05}, p=3.89×10^{-03}, Table 2). Lead variants in the *GNA12* locus associated with t-tau and p-tau181 were also significant for increased AD risk (OR=1.020, CI=1.002-1.038, p=2.51×10^{-02} for t-tau variant; OR=1.029; CI=1.011-1.047, p=1.24×10^{-03} for p-tau181 variant; Table 2).

Page 16, line 3: In line with their associations with lower CSF t-tau and p-tau181 levels, both alleles from the analysis were also significantly associated with reduced risk of AD (t-tau variant OR=0.928, CI=0.913-0.943, p=2.08×10^{-19} ; p-tau181 variant OR=0.918, CI=0.903-0.933, p=1.65×10^{-24}; Table 2), with 96% and 99% probability of being the causal variants within the locus for AD risk as well, based on colocalization evidence (Supplementary Table 9).

2. **In the cross-ancestry comparison, the comparisons have issues with presentation, not really any such thing as nominal or suggested significance, either its significant after multi-test correction or not. Can't be nominally or suggestively dead. worth deleting or revising. Additionally, if P is > 0.05 after correction, effect estimates cannot be accurately quantified in terms of directionality, so I'd suggest removing that part too.**

Response: We agree with the reviewer comments that if P>0.05, commenting on directionality does not hold true. We have now removed the comment on directionality of variants between analyses from the section. However, our cross-ancestry analysis is exploratory in nature and includes limited sample size (N=416). Thus, trend of association of well-established signals (eg: *APOE* variant rs429358) and signals observed in main analysis for European population may be of interest to readers and can also act as reference for future studies. Thus, we believe presenting these findings despite not passing genome wide significance threshold serves a valid purpose. In line with the reviewer's comment, we have now modified the section to remove suggestion of nominal significance and have further emphasized the exploratory nature of the analysis.

We have now made the following changes in the manuscript:

Page 11, line 21-30:

The analysis in the non-European population revealed that association of *APOE* locus (rs429358) with Aβ42 is significant (BETA=-0.695, SE=0.079, $p=8.02\times10^{-17}$; Supplementary Table 1, Supplementary Fig. 12). The *APOE* variant, that was associated with both t-tau and p-tau181 in main analysis, did not pass genome wide significance but showed trends toward significant association ($p_{t\text{-}tau}=3.17\times10^{-03}$, $p_{p\text{-}tau181}=6.25\times10^{-04}$). Similarly, the variant rs10779336 in the *CR1* locus on chromosome 1 ($p=1.24\times10^{-02}$ for Aβ42) and the variant rs6733839 in the *BIN1* locus ($p_{t\text{-}tau}= 5.53\times10^{-02}$; $p_{p\text{-}tau181}= 7.42\times10^{-02}$) showed nominal associations ($p<0.05$; Supplementary Table 1). The trend of association in signals observed in this exploratory analysis, despite the limited sample size, suggests potentially meaningful genetic effects in non-European samples as well that need further investigation in larger cohorts.

3. **Please include lambda and LD intercepts in QC section.**

Response: We thank the reviewer for highlighting additional QC metrices that further support the quality of our analysis and data. We have now calculated and presented genomic inflation factor (λ) and LD intercepts for all three biomarker summary statistics from meta-analysis. Additionally, we have also calculated and presented the λ for individual summary statistics from each contributing sites, which are included as supplementary information along with other QC details.

We have now made the following changes in the manuscript to address this point:

Page 7, line 15: The genomic inflation lambda(λ) for the meta-analysis summary statistics was 1.07 to 1.08 (Supplementary Fig. 8). The LD Score regression intercept was approximately equal to the genomic control inflation: 1.04 for Aβ42 and 1.055 for both t-tau and p-tau181, suggesting minimal inflation due to confounding factors.

Page 27, line 29: Genomic inflation was assessed for all contributing summary statistics (Supplementary Table 22). Presence of confounding bias in meta-analysis summary statistics was also evaluated using Linkage Disequilibrium Score Regression (LDSC) command line tool[1].

Supplementary Table 22: Sample Genotype quality control and analysis detail in each cohort

**Cohort**	GWAS Lambda
Knight ADRC	Aβ42: 1.012; t-tau:1.003,p-tau-181: 1.011
EADB	Aβ42: 1.009; t-tau:0.982,p-tau-181: 0.953
EMIF-AD	Aβ42: 1.009; t-tau:0.993,p-tau-181: 0.990
MISSION-AD	Aβ42: 0.999; t-tau:1.006,p-tau-181: 1.001
ACE	Aβ42: 1.000; t-tau:1.011,p-tau-181: 1.007
ALFA	Aβ42: 1.001; t-tau:1.014,p-tau-181: 1.015
EPAD	Aβ42: 0.999, t-tau:0.999,p-tau-181: 1.001
Janssen	Aβ42: 0.992 & 0.997,t-tau:0.999 & 1.011,p-tau-181: 0.993 & 1.022

Note: Only relevant supplementary table 22 columns shown here. Complete table included in manuscript has additional columns.

4. **Was phenotype included in analyses as covariate for sensitivity or stratification and comparison via I2 for hits at least?**

Response: We thank the reviewer for this important question, In the analyses performed in the initial submission we included age and sex as covariates. We did not include disease status in the model.

We understand that the reviewer is asking to perform additional sensitivity analyses by stratifying by disease status.

We have now performed diagnosis stratified GWAS in Alzheimer disease (AD) and healthy control (CO) to see if any meta-analysis findings were driven by either diagnosis group. A GWAS including an interaction with status was also performed to assess effect of the diagnosis status on the biomarkers. These analyses were performed using samples that had raw data and disease status available. In total, 2,365 AD cases and 2,763 controls were available for stratified analysis.

In the AD-specific analyses, significant associations were found for chromosome 19 for Aβ42, t-tau and p-tau181. Within CO, in addition to the chromosome19 association with the three biomarkers, association of *GMNC* locus on chromosome 3 with t-tau and p-tau181 were also identified. Even if this signal is not genome-wide in AD cases, it shows a suggestive association and additional analyses indicate that the effect size of this variant in AD cases or controls is not significantly different.

We also identified some novel loci when stratified by disease status, including a locus on chromosome 3 (rs71306566[C]), in the *AADACL2-AS1* gene region, that showed association with Aβ42 in AD and a locus on chromosome 5 (rs75840268[T]), located near *EPB41L4A* in CO. The rs71306566 variant had consistent direction in cases and controls and was significantly different compared to both meta-analysis ($p<8.32\times10^{-05}$) and CO groups (p=0.0018). This locus did not have any significant effect on AD risk (p= 0.318), progression (p= 0.789) or brain amyloidosis (p=0.107). Similarly, rs75840268 had significantly different effect size between CO and AD (6.44×10^{-06}) as well as with the meta-analysis (p=0.00028) with an opposite direction of effect between AD and CO. This variant showed a nominal association with AD risk (p=0.027) and brain amyloidosis (p=0.01). We did not observe any significant colocalization of this locus with CSF pQTLs or brain eQTLs and therefore could not implicate a functional gene. The rs192462264[A] variant on chromosome 7 was close to genome wide significance in AD but failed to pass the threshold ($p=1.178\times10^{-07}$).

In the SNP-Diagnosis interaction analysis, no variants reached genome wide significance threshold. This finding could be potentially due to limited statistical power to detect minor difference in effect between the diagnosis group or could be because the variants identified in our meta-analysis modulate biomarker levels consistently across both diagnosis group.

Supplementary Table 5: Table showing effect size for variants from meta-analysis in AD and CO stratified analysis

						Meta-analysis		CO-only		AD-only	
**MarkerName**	**rsID**	**Trait**	**EA**	**MAF**	**Closest Gene**	**Effect**	**P**	**Effect**	**P**	**Effect**	**P**
chr1:207625371:T:G	rs10779336	Aβ42	T	0.198	*CR1*	-0.073	**3.49E-08**	-0.011	7.17E-01	-0.05	1.31E-01
chr2:127135234:C:T	rs6733839	Aβ42	T	0.394	*BIN1*	-0.060	**8.29E-09**	-0.031	2.15E-01	-0.049	7.63E-02
chr19:44908684:T:C	rs429358	Aβ42	C	0.228	*APOE*	-0.589	**1.90e-674**	-0.535	**1.17E-61**	-0.476*	**4.26E-64**
chr2:127135234:C:T	rs6733839	t-tau	T	0.394	*BIN1*	0.060	**5.24E-09**	-0.003*	8.95E-01	0.063	3.08E-02
chr3:190953006:C:T	rs35327527	t-tau	T	0.369	*GMNC*	0.151	**3.45E-48**	0.164	**1.13E-11**	0.109	2.36E-04
chr7:2761908:G:A	rs798490	t-tau	A	0.298	*AMZ1-GNA12*	-0.074	**1.87E-11**	-0.057	2.31E-02	-0.049	1.16E-01
chr8:107381731:A:G	rs1654723	t-tau	A	0.411	*ANGPT1*	0.056	**4.41E-08**	0.074	1.75E-03	0.034	2.43E-01
chr9:2197987:A:G	rs57263785	t-tau	G	0.248	*SMARCA2*	0.087	**1.12E-13**	0.065	1.53E-02	0.092	6.32E-03
chr11:60207692:G:A	rs7928895	t-tau	A	0.393	*MS4A4E*	-0.060	**5.35E-09**	-0.032	1.74E-01	-0.081	5.99E-03
chr12:21279895:C:T	rs11045930	t-tau	T	0.288	*SLCO1A2*	0.065	**3.26E-09**	0.034	1.85E-01	0.047	1.35E-01
chr16:87199910:G:A	rs4843552	t-tau	G	0.409	*C16orf95*	0.085	**1.30E-16**	0.099	2.35E-05	0.089	2.19E-03
chr19:44908684:T:C	rs429358	t-tau	C	0.228	*APOE*	0.301	**5.40E-161**	0.164*	1.89E-07	0.192*	**1.82E-10**
chr2:127135234:C:T	rs6733839	p-tau181	T	0.394	*BIN1*	0.073	**2.28E-12**	0.031	2.22E-01	0.081	9.61E-03
chr3:190953006:C:T	rs35327527	p-tau181	T	0.369	*GMNC*	0.153	**1.43E-47**	0.170	**8.77E-12**	0.121	1.22E-04
chr7:2718675:A:G	rs798560	p-tau181	G	0.298	*AMZ1*	-0.065	**5.81E-09**	-0.030	2.41E-01	-0.024	4.80E-01
chr9:2197987:A:G	rs57263785	p-tau181	G	0.248	*SMARCA2*	0.091	**2.06E-14**	0.086	1.83E-03	0.111	2.00E-03
chr11:60254475:G:A	rs1582763	p-tau181	A	0.365	*MS4A4E*	-0.059	**2.94E-08**	-0.057	2.22E-02	-0.073	2.36E-02
chr12:21279895:C:T	rs11045930	p-tau181	T	0.288	*SLCO1A2*	0.065	**6.24E-09**	0.034	1.97E-01	0.017	6.02E-01
chr16:87199910:G:A	rs4843552	p-tau181	G	0.409	*C16orf95*	0.081	**7.28E-15**	0.085	4.38E-04	0.078	1.14E-02
chr19:44908684:T:C	rs429358	p-tau181	C	0.228	*APOE*	0.318	**3.27E-174**	0.220*	**1.00E-11**	0.251*	**4.82E-15**

* Denotes significant difference (p<0.05) between effect sizes compared to meta-analysis examined using t-test of the estimates from each analysis

Supplementary Table 5: showing effect size variants identified in AD and CO stratified analysis

					**AD-only analyses**		**CO-only analyses**		**Meta-analysis**	
	ID	rsID	Phenotype	A1	BETA	P	BETA	P	BETA	P
AD	chr3:151922613:T:C	rs7130656	Aβ42	C	0.247#,*	**1.80E-08**	0.065	9.17E-02	0.063	1.38E-04
AD	chr19:44919689:A:G	rs4420638	Aβ42	G	-0.470*	**4.08E-65**	-0.456	**8.36E-53**	-0.527	**5.00e-537**
AD	chr19:44919689:A:G	rs4420638	t-tau	G	0.212	**6.70E-13**	0.140	**1.23E-06**	0.272	**2.06E-132**
AD	chr19:44919689:A:G	rs4420638	p-tau181	G	0.250	**1.01E-15**	0.170	**8.31E-09**	0.279	**2.39E-136**
CO GWAS	chr19:44908684:T:C	rs429358	Abeta	C	-0.476	**4.26E-64**	-0.527	**1.17E-61**	-0.589	**1.90e-674**
CO GWAS	chr3:190953006:C:T	rs35327527	t-tau	T	0.109	2.36E-04	0.163	**1.13E-11**	0.151	**3.45E-48**
CO GWAS	chr5:112241984:G:T	rs75840268	t-tau	T	-0.202	1.75E-01	0.641#,*	**1.75E-08**	0.189	2.00E-04
CO GWAS	chr19:44888997:C:T	rs6857	t-tau	T	0.195	1.23E-10	0.172*	**1.21E-08**	0.275	**1.04E-74**
CO GWAS	chr3:190953006:C:T	rs35327527	p-tau181	T	0.121	1.22E-04	0.170	**8.77E-12**	0.153	**1.43E-47**
CO GWAS	chr19:44908684:T:C	rs429358	p-tau181	C	0.251	**4.82E-15**	0.220*	**1.00E-11**	0.318	**3.27E-174**

* Denotes significant difference (p<0.05) between effect sizes compared to meta-analysis.
# Denotes significant difference (p<0.05) between effect sizes compared.

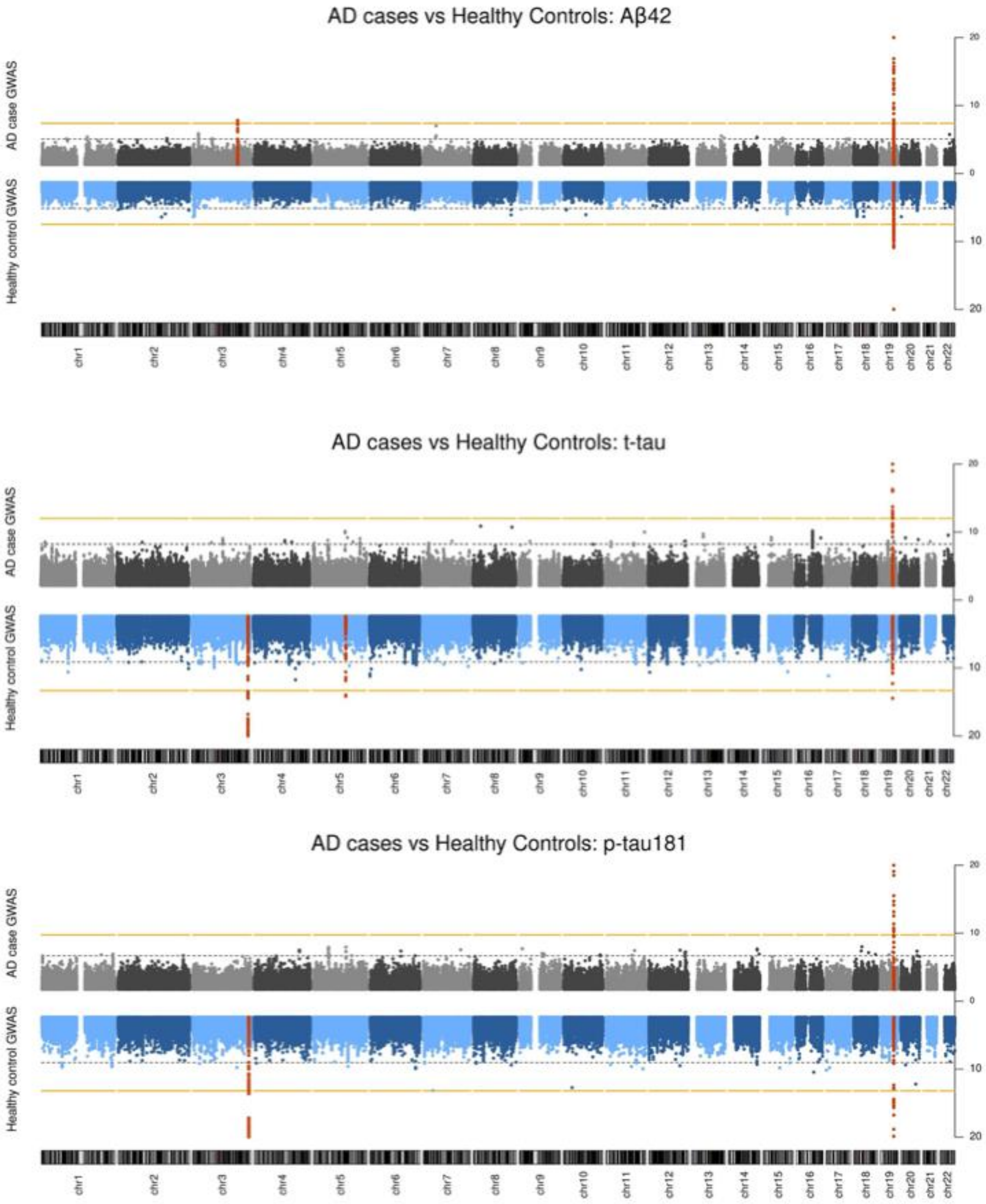

***Reply Figure 1 (Supplementary Figure 13)**: Miami plot showing GWAS results from AD/CO stratified analysis. Plots on top are from AD only GWAS and plot on the bottom are from CO only GWAS. Red highlighted region represents loci that passed genome-wide threshold in corresponding analysis.*

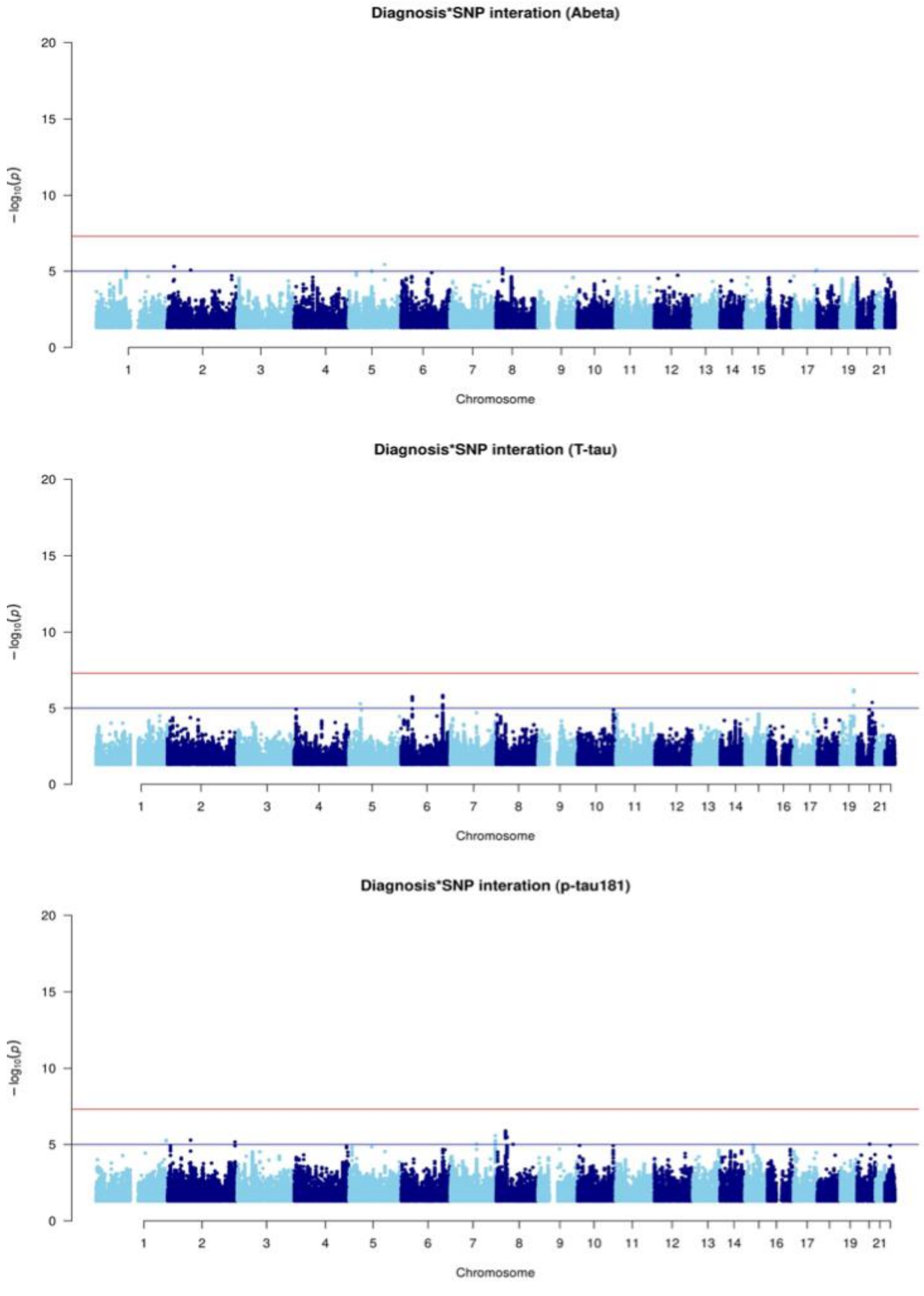

Reply Figure2 (Supplementary Figure 15) : *Manhattan plot showing results from interaction term (SNP*Diagnosis) GWAS. Red line represents genome-wide significance threshold. Blue line represents suggestive significance threshold. None of the variants tested passed genome wide significance in the analysis.*

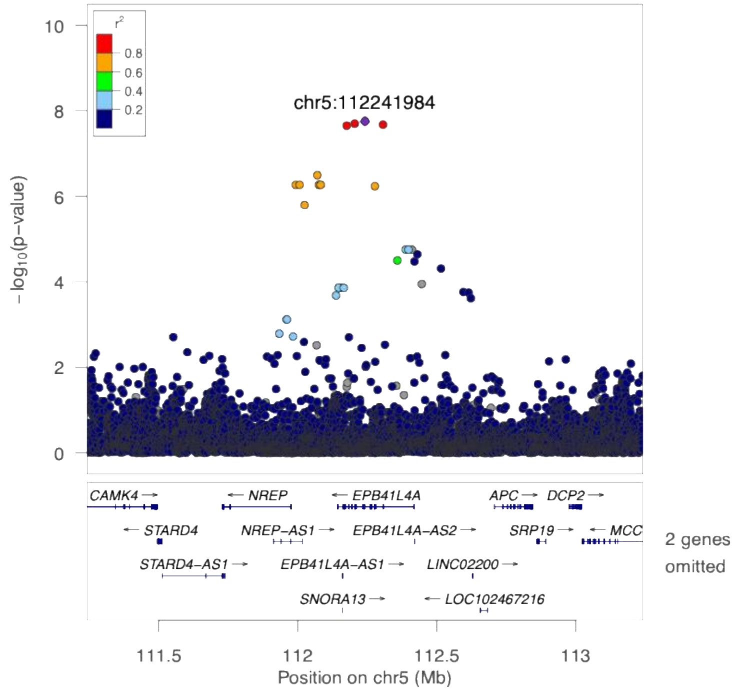

Reply Figure 3 (Supplementary Figure 14) *Locus zoom plot showing the chromosome 5 variant associated with t-tau levels in controls.*

We have now added these analyses as new section in the manuscript:

Page12, line1:

Diagnosis Stratified Analysis

We performed stratified GWAS in Alzheimer disease (AD) and healthy control (CO) to see if any meta-analysis finding were driven by either diagnosis group. A GWAS including an interaction with status was also performed to assess effect of the diagnosis status on the biomarkers.

In the AD-specific analyses, significant associations were found for chromosome 19 for Aβ42, t-tau and p-tau181. Within CO, in addition to the chromosome19 association with the three biomarkers, association of *GMNC* locus on chromosome 3 with t-tau and p-tau181 were also identified (Supplementary Fig. 13, Supplementary Table 5). Even if this signal was not genome-wide in AD cases, it showed a suggestive association and additional analyses indicate that the effect size of this variant in AD cases or controls is not significantly different. We also identified some novel loci when stratified by disease status, including a locus on chromosome 3 (rs71306566[C]), in the *AADACL2-AS1* gene region, that showed association with Aβ42 in AD and a locus on chromosome 5 (rs75840268[T]), located near *EPB41L4A* in CO*.* The rs71306566 variant had consistent direction in cases and controls and was significantly different in AD compared to both meta-analysis ($p=8.32\times10^{-05}$) and CO groups ($p=0.002$; Supplementary Table 5). This locus did not have any significant effect on AD risk ($p= 0.318$), progression ($p= 0.789$) or brain amyloidosis ($p=0.107$). Similarly, rs75840268 had significantly different effect size between CO and AD ($p=6.44\times10^{-06}$) and with the meta-analysis ($p=2.8\times10^{-04}$) with an

opposite direction of effect between AD and CO (Supplementary Fig. 13,14). This variant showed a nominal association with AD risk (p=0.027) and brain amyloidosis (p=0.010). We did not observe any significant colocalization of this locus with CSF pQTLs or brain eQTLs and therefore could not implicate a functional gene. The rs192462264[A] variant on chromosome 7 was close to genome wide significance in AD but failed to pass the threshold ($p=1.178\times10^{-07}$; Supplementary Fig. 13).
In the SNP-Diagnosis interaction analysis, no variants reached genome wide significance threshold (Supplementary Fig. 15). This finding could be potentially due to limited statistical power to detect minor difference in effect between the diagnosis group or could be because the variants identified in our meta-analysis modulate biomarker levels consistently across both diagnosis group.

Page 16, line 21: Chromosome 5 loci associated with t-tau in CO, identified in diagnosis stratified analysis, showed nominal association with AD risk (p=0.027) and brain amyloidosis (p=0.010).

Page 24, line 2: Disease stratified analysis identified a chromosome 5 signal specific to controls that was nominally associated with both AD risk and brain amyloidosis. But due to lack of any colocalization evidence, we were unable to identify functional gene in the locus. However, because this analysis was limited to samples with raw data, future studies with larger sample size may be able to better characterize this locus.

Page 26, line 20: Similarly, diagnosis stratified analyses were performed in AD and CO group using samples that had both raw data and disease status available. In total, 2365 AD cases and 2763 CO were available for the diagnosis stratified analysis.

Page 28, line 12: Diagnosis stratified analysis were performed using samples that had raw data and disease status available. Diagnosis was based on CDR at the time of sample collection or clinically assigned status.

Figures and Tables presented above have now been added as supplementary figures and tables.

5. **All fixed effect meta-analyses should include I2 or other heterogeneity metrics as standard practice please.**

Response: We agree with the reviewer. We have now added I^2 for all signals presented in the paper. The final summary statistics associated with this project will include the heterogeneity measure for all SNPs.

We have now made the following changes in manuscript:

Page 8, line 14: The variant rs6733839[T] in the *BIN1* locus was significantly associated with CSF Aβ42 (Effect=-0.06, SE=0.011, $p=8.29\times10^{-09}$, $I^2=0$; Table 2; Supplementary Fig. 1).

Page 8, line 17: This variant was also found to influence t-tau and p-tau181 levels in the meta-analysis with consistent direction of effects as in previous studies ($Effect_{t\text{-}tau}=0.06$, $SE_{t\text{-}tau}=0.010$, $p_{t\text{-}tau}=5.24\times10^{-09}$, $I^2_{t\text{-}tau}=48.1$; $Effect_{ptau181}=0.073$, $SE_{p\text{-}tau181}=0.010$, $p_{ptau181}=2.28\times10^{-12}$, $I^2=10.8$; Table 2; Supplementary Fig. 2,5; Supplementary Table 1).

Page 8, line 22: The variant rs798490[A] (MAF=0.298), located in the 7p22.3 region near *GNA12* and *AMZN1*, was associated with t-tau (Effect=-0.074, SE=0.011, $p=1.87\times10^{-11}$, $I^2=0$, Supplementary Fig. 2).

Page 8, line 26: An additional novel locus on 9p24.3 (rs57263785[G] near *SMARCA2*) showed significant associations with both t-tau and p-tau181 ($Effect_{t\text{-}tau}=0.087$, $SE_{t\text{-}tau}=0.012$, $p_{t\text{-}tau}=1.12\times10^{-13}$, $I^2_{t\text{-}tau}=0$; $Effect_{p\text{-}tau181}=0.091$, $SE_{p\text{-}tau181}=0.012$, $p_{p\text{-}tau181}=2.06\times10^{-14}$, $I^2_{p\text{-}tau181}=0$; Table 2, Supplementary Fig. 3, 6).

Page 9, line 4: Another novel association for both t-tau and p-tau181 was identified on chromosome 12 (rs11045930[T] within *SLCO1A2;* $Effect_{t\text{-}tau}=0.065$, $SE_{t\text{-}tau}=0.011$, $p_{t\text{-}tau}=3.26\times10^{-09}$, $I^2_{t\text{-}tau}=0$; $Effect_{p\text{-}tau181}=0.065$, $SE_{p\text{-}tau181}=0.011$, $p_{p\text{-}tau181}=6.24\times10^{-09}$, $I^2_{p\text{-}tau181}=0$; Supplementary Fig. 4,7). Finally, a novel genetic association on 8q23.1 near *ANGPT1* and unique to t-tau was also identified (rs1654723[A]: Effect=0.056, $SE_{t\text{-}tau}=0.010$, $p= 4.41\times10^{-08}$, $I^2_{t\text{-}tau}=0$, MAF=0.411; Supplementary Fig. 3).

6. **Code, data availability and other FAIR / transparency related information is missing**

Response: We have now added following statement to clarify availability of data and codes.

GWAS meta-analysis summary statistics will be freely available through the GWAS Catalog (https://www.ebi.ac.uk/gwas/) and through the NeuroGenomics & Informatics (NGI) Center website (https://neurogenomics.wustl.edu/).

Individual-level data for the following cohorts can be requested through these resources:

1. **ADNI and ADNI DOD:** CSF proteomic & genomic data (https://adni.loni.usc.edu/)
2. **DIAN:** CSF proteomic & genomic data (https://dian.wustl.edu/our-research/for-investigators/diantu-investigator-resources/dian-tu-biospecimen-request-form/)
3. **Knight-ADRC:** CSF & plasma proteomic & genomic data (https://knightadrc.wustl.edu/data-request-form/)
4. **PPMI:** CSF proteomic & genomic data (https://www.ppmi-info.org/access-data-specimens/download-data)
5. **MARS:** Participants from the WashU Movement Disorder Clinic have not given consent for individual-level data sharing.
6. **Barcelona-1:** Data from the Barcelona-1 cohort is not publicly available due to GDPR restrictions.
7. **AIBL:** Data from this cohort can be requested at https://aibl.org.au/
8. BIOCARD: Data for samples included in this cohort can be obtained from https://biocard.pathology.jhu.edu/about-the-biocard-study/
9. **ALFA+:** All requests for raw and analysed data and materials will be promptly reviewed by the Barcelonaβeta Brain Research Center scientific committee to verify whether the request is subject to any intellectual property or confidentiality obligations. Bulk Anonymized data can be shared by request from any qualified investigator for the sole purpose of replicating procedures and results presented in the article, providing data transfer is in agreement with EU legislation.
10. **FACE:** Data from the FACE cohort is not publicly available due to GDPR restrictions. Phenotypic data for samples included in this study are available upon request to qualified researchers.
11. **MISSION-AD:** Genotype data are not available due to data sharing restrictions. Summary statistics used in this project and phenotype data are available upon reasonable request to qualified researchers.
12. **EADB:** In accordance with GDPR restrictions, individual-level data from EADB is not available publicly. Please contact s.j.vanderlee@amstrdamumc.nl.
13. **EMIF:** In accordance with EU law and participant privacy, clinical individual-level data from EMIF-AD is not available publicly but can be obtained via EMIF-AD (https://emif-catalogue.eu; http://www.emif.eu/about/emif-ad).
14. **EPAD:** Data used in this study can be downloaded or requested from https://ep-ad.org/index.php/open-source-data/

15. **Janssen:** Part of the data used in this project is available to download from Vivli.com. Data not available to download form the site are available upon request on a case-by-case basis.
    All other cohorts not listed individually were downloaded from NIAGADS repository.

We used publicly available software for all analyses in this study. This included: PLINK 2 version 2.00a3.6LM for all single variant analysis, METAL (https://csg.sph.umich.edu/abecasis/metal/download/, for meta-analysis, mtag (Multi-Trait Analysis of GWAS; https://github.com/JonJala/mtag) for pleiotropy analysis, TwoSampleMR R package (version 0.5.8) to perform MR and the coloc R package (version 5.2.2) to perform colocalization. Pathway analysis was performed using the clusterProfiler (version 4.8.2), org.Hs.eg.db (version 3.17.0), DOSE (version 3.26.1) and ReactomePA (version 1.44.0) R packages. LD Score Regression (LDSC; https://github.com/bulik/ldsc) and GNOVA (https://github.com/xtonyjiang/GNOVA) were used for genetic covariance test. Variant annotation and gene-based analysis were performed using ANNOVAR and MAGMA as part of FUMA pipeline (https://fuma.ctglab.nl/) .

We have now added this code availability statement to **page 39** in the main manuscript.

Reviewer 4 comments

1. **Meta-analysis of the summary statistics across the endophenotypes using METAL is non-standard and does not account for the same samples being used multiple times and the measurements being correlated. Many options exist for such an analysis of pleiotropic effects. Consider using min-P methods, MOSTest, pleiotropic association test, or MTAG: https://www.nature.com/articles/s41467-020-17368-1, https://journals.plos.org/plosgenetics/article?id=10.1371/journal.pgen.1010447.**

Response: We thank the reviewer for this very important question and recommendations for alternative tools. While we agree with need for accounting pleiotropic effects, the concerns on use of METAL for meta-analysis does not hold true in context of our study design. Here, METAL was implemented independently within each biomarker and not across the traits. So, while same individuals contributed to the three biomarkers, each analysis was carried out separately as independent tests. Meta-analysis was used to combine data across cohorts (not traits), which is a standard use case of METAL in GWAS[2-4]. Therefore, concerns regarding correlation between biomarkers or repeated use of samples across traits are not applicable to our study design. Further, we verified that there was no sample overlap between contributing cohorts (through identity-by-descent) prior to analysis, thus, within each biomarker sample duplication is not a potential concern.

In any case and in order to further address the reviewer's comment, we have now performed pleiotropy accounted analysis between the three biomarkers using MTAG. Because MTAG is susceptible to increased false positives when SNPs do not have a homogenous effect across traits being compared, as is the case for our phenotypes of interest, we applied an orthogonal pairwise conjunctional false discovery rate (FDR) analysis using a previously established approach[5,6]. A signal was deemed to be a true signal if it passed two sets of criteria: 1. Genome wide significant in MTAG 2. Passes conjunctional FDR $p<0.05$ for pleiotropy with either of the remaining two biomarker. Using these criteria, we identified seven genome-wide significant loci for Aβ42, nine for t-tau and eight for p-tau181. Association of chromosome 3,7,9,12 and 16 were found for both t-tau and p-tau181. These loci were consistent with association identified in METAL meta-analysis. Association of *APOE* with CSF Aβ42 was also identified but its association with t-tau and p-tau was not observed using MTAG. This could potentially be due to the very significant association of this locus with Aβ42 ($p=10^{-600}$ vs $p=10^{-100}$ in t-tau/p-tau181 from METAL) resulting in MTAG treating it as trait specific association or because the effect of *APOE* on t-tau and p-tau181 is not independent of Aβ42. However, previous research has shown that although part of *APOE*-t-tau/ptau181 interaction is mediated through Aβ42, some variants within the region affect tau/ptau-181 independently[7]. Associations of chromosome 7,9 and 16 with Aβ42 were novel but were same as those identified for t-tau and p-tau-181. These findings suggest that there is some interaction between Aβ42 and t-tau/ptau181 in these loci.

In addition to these known association, there were some novel loci identified by MTAG. These included: a locus on chromosome 2 near *GMCL1,* a locus on chromosome 5 near *MTREX* and a locus on chromosome 6 near *PLEKHG1* associated with Aβ42, t-tau and p-tau181. An additional locus near *PPP2R2B* in chromosome 5 was associated with t-tau only. For loci that were unique to MTAG analysis, we investigated the effect of the lead variant in these loci on other AD related traits and conducted follow-up colocalization analysis for any loci that showed significant effect. The Chromosome 6 variant and the chromosome 5 variant associated with t-tau only were not associated with any of the AD related traits. However, both chromosome 2 and 5 variants were nominally associated with brain amyloidosis (p_{chr2}=0.023; 0.016; p_{chr5}=0.022). Colocalization analyses were then performed for these two loci with CSF pQTLs and brain eQTLs to identify potential functional genes. Chromosome 2 locus colocalized with eQTLs for either *GMCL1*, SNRNP27 or *ANXA4* depending on brain region. In addition, associations with t-tau and p-tau181 also colocalized with CSF pQTL for *ANXA4.* Because *ANXA4* had colocalization evidence from both GTEx v8 Brain eQTL and CSF pQTL for tau-181 and ptau-181, we nominate the gene as most likely functional gene for the locus. *ANAX* family of genes have been shown to increase in brain of AD patients and play a role in Aβ clearance and also contribute to the localization of tau in the axon[8,9]. We did not observe colocalization of Aβ42 association with pQTL for *ANXA4* (PP.H4=0.536), thus *GMCL1* is most likely the functional for Aβ42 association at chromosome 2 based on evidence of colocalization from GTEx v8 Brain eQTL and MetaBrain. Overexpression of *GMCL1* has been linked to certain type of cancers however there is no direct evidence linking it to AD[10]. In light of these findings, we hypothesize that the chromosome 2 locus has trait specific regulatory mechanisms. Similarly, chromosome 5 locus colocalized with brain eQTLs for *CCNO* and *MCIDAS* gene. In tau and ptau-181 colocalization was observed in both GTEx v8 Brain eQTL and MetaBrain eQTL but in Aβ42 this was only found in GTEx v8 Brain eQTL. *CCNO* had evidence from both GTEx and MetaBrain; thus, *CCNO* is likely the functional of the two genes. The *CCNO* gene has been reported to control other genes in AKT signaling cascade which is an important disease mechanism in AD[9,11].

Overall, after accounting for pleiotropy between the biomarkers, we observed signals previously identified in our meta-analysis as well as few novel signals that point to additional disease mechanism.

Supplementary Table 3: Table showing signal identified as significant after pleiotropy adjustment

							**pleioFDR**			**METAL meta-analysis**	
**Marker Name**	**rsID**	**Pheno**	**CHR**	**Mtag_ beta**	**mtag_ pval**	**pleioFDR_ Aβ vs Tau**	**pleioFDR_ Aβ vs pTau**	**pleioFDR_ Tau vs pTau**	**Meta_ Abeta**	**Meta_ Tau**	**Meta_ pTau**
chr2:69830316:T:G	rs3732266	Aβ42	2	-0.05	**1.53E-08**	**1.02E-02**	**6.40E-03**		8.54E-05		
chr5:55370326:A:G	rs6450304	Aβ42	5	0.09	**3.52E-11**	**3.38E-04**	**3.53E-04**		1.01E-05		
chr6:150720216:C:T	rs9383542	Aβ42	6	-0.05	**7.22E-09**	**2.33E-02**	**2.49E-02**		9.74E-04		
chr7:2763403:A:G	rs798486	Aβ42	7	-0.07	**5.66E-16**	**8.71E-05**	**9.14E-05**		**2.37E-06**		
chr9:2197987:A:G	rs57263785	Aβ42	9	0.07	**1.32E-13**	**3.08E-02**	**3.31E-02**		1.32E-03		
chr16:87190687:A:G	rs12711473	Aβ42	16	0.07	**2.77E-18**	**1.25E-04**	**1.31E-04**		**3.49E-06**		
chr19:44906745:G:A	rs769449	Aβ42	19	-0.21	**6.76E-85**	**1.77E-28**	**1.76E-28**		**8.39E-301**		
chr2:69841503:G:T	rs2872075	t-tau	2	-0.050	**5.77E-09**	1.45E-01		**9.75E-05**		**2.33E-06**	
chr3:190951729:G:A	rs9877502	t-tau	3	0.140	**7.22E-51**	1.66E-01		**1.44E-28**		**4.68E-48**	
chr5:147030865:C:T	rs78652028	t-tau	5	0.170	**3.48E-08**	1.77E-01		**1.09E-03**		**6.16E-07**	
chr5:55385798:C:T	rs62360531	t-tau	5	0.100	**4.61E-11**	**8.64E-04**		**1.12E-04**		**1.38E-06**	
chr6:150697773:T:G	rs6940540	t-tau	6	-0.060	**1.98E-09**	**4.72E-02**		**1.94E-04**		**1.03E-06**	
chr7:2761908:G:A	rs798490	t-tau	7	-0.080	**1.42E-16**	**1.91E-04**		**8.23E-07**		**1.87E-11**	
chr9:2197987:A:G	rs57263785	t-tau	9	0.090	**4.25E-18**	**3.08E-02**		**3.03E-11**		**1.12E-13**	
chr12:21282196:T:C	rs2417972	t-tau	12	0.070	**1.33E-10**	4.22E-01		**1.29E-06**		**3.81E-09**	
chr16:87199910:G:A	rs4843552	t-tau	16	0.090	**1.93E-21**	**3.78E-03**		**2.24E-12**		**1.30E-16**	
chr2:69841503:G:T	rs2872075	p-tau181	2	-0.050	**5.83E-09**		0.145	**9.75E-05**			**1.07E-06**
chr3:190951729:G:A	rs9877502	p-tau181	3	0.140	**1.50E-51**		0.166	**1.44E-28**			**1.82E-47**
chr5:55385798:C:T	rs62360531	p-tau181	5	0.100	**7.37E-11**		**8.64E-04**	**1.12E-04**			**2.98E-06**
chr6:150697773:T:G	rs6940540	p-tau181	6	-0.060	**2.82E-09**		**0.047**	**1.94E-04**			**6.81E-09**
chr7:2761908:G:A	rs798490	p-tau181	7	-0.080	**3.93E-16**		**1.91E-04**	**8.23E-07**			**8.95E-09**
chr9:2197987:A:G	rs57263785	p-tau181	9	0.090	**3.50E-18**		**0.031**	**3.03E-11**			**2.06E-14**
chr12:21279895:C:T	rs11045930	p-tau181	12	0.070	**1.26E-10**		0.490	**6.00E-07**			**6.24E-09**
chr16:87199910:G:A	rs4843552	p-tau181	16	0.080	**2.93E-21**		**3.7E-03**	**2.24E-12**			**7.28E-15**

p-values highlighted bold are those that passed significance threshold in corresponding analysis

Supplementary Table 4: Colocalization results of MTAG signals with Brain eQTL and cis pQTL resources that had PP.H4>0.8

nsnps	PP.H0.abf	PP.H1.abf	PP.H2.abf	PP.H3.abf	PP.H4.abf	Tissue	Symbol	Locus_CHR	Locus_BP	Phenotype	Reference
135	6.09E-05	0.011	9.85E-04	0.184	0.804	Anterior_cingulate_cortex_BA24	SNRNP27	chr2	69830316	Ab42	GTEx
5	0.036	0.039	0.004	0.004	0.918	Caudate_basal_ganglia	ANXA4	chr2	69830316	Ab42	GTEx
129	1.44E-05	0.003	2.37E-04	0.056	0.941	Cerebellar_Hemisphere	GMCL1	chr2	69830316	Ab42	GTEx
210	1.94E-10	4.78E-08	1.71E-04	0.041	0.959	Cerebellum	GMCL1	chr2	69830316	Ab42	GTEx
198	3.24E-07	7.90E-05	4.70E-04	0.114	0.886	Cerebellum	SNRNP27	chr2	69830316	Ab42	GTEx
133	8.32E-08	2.04E-05	1.95E-04	0.047	0.953	Cortex	GMCL1	chr2	69830316	Ab42	GTEx
17	0.001	0.029	4.44E-04	0.009	0.96	Frontal_Cortex_BA9	GMCL1	chr2	69830316	Ab42	GTEx
191	1.25E-09	3.01E-07	3.87E-04	0.093	0.907	Frontal_Cortex_BA9	SNRNP27	chr2	69830316	Ab42	GTEx
218	2.93E-09	7.23E-07	1.11E-04	0.027	0.973	Hippocampus	SNRNP27	chr2	69830316	Ab42	GTEx
23	6.32E-05	0.011	1.02E-04	0.017	0.972	Hypothalamus	GMCL1	chr2	69830316	Ab42	GTEx
375	4.15E-10	1.02E-07	8.57E-05	0.02	0.98	cerebellum	GMCL1	chr2	69830316	Ab42	MetaBrain
4	0.013	0.024	0.002	0.002	0.959	Anterior_cingulate_cortex_BA24	GMCL1	chr2	69841503	Tau	GTEx
135	8.17E-06	0.009	1.32E-04	0.148	0.843	Anterior_cingulate_cortex_BA24	SNRNP27	chr2	69841503	Tau	GTEx
5	0.007	0.047	0.002	0.009	0.935	Caudate_basal_ganglia	GMCL1	chr2	69841503	Tau	GTEx
5	6.50E-04	0.04	7.73E-05	0.004	0.955	Caudate_basal_ganglia	ANXA4	chr2	69841503	Tau	GTEx
129	3.50E-06	0.004	5.76E-05	0.064	0.932	Cerebellar_Hemisphere	GMCL1	chr2	69841503	Tau	GTEx
210	3.48E-11	5.86E-08	3.07E-05	0.051	0.949	Cerebellum	GMCL1	chr2	69841503	Tau	GTEx
198	7.94E-08	1.08E-04	1.15E-04	0.156	0.843	Cerebellum	SNRNP27	chr2	69841503	Tau	GTEx
133	1.61E-08	2.61E-05	3.77E-05	0.06	0.94	Cortex	GMCL1	chr2	69841503	Tau	GTEx
17	9.58E-04	0.048	3.28E-04	0.015	0.936	Frontal_Cortex_BA9	GMCL1	chr2	69841503	Tau	GTEx
191	2.75E-10	3.73E-07	8.54E-05	0.115	0.885	Frontal_Cortex_BA9	SNRNP27	chr2	69841503	Tau	GTEx
2	0.033	0.038	0.002	0.002	0.925	Hippocampus	GMCL1	chr2	69841503	Tau	GTEx
218	1.03E-09	1.74E-06	3.93E-05	0.065	0.935	Hippocampus	SNRNP27	chr2	69841503	Tau	GTEx
23	1.95E-05	0.017	3.13E-05	0.026	0.957	Hypothalamus	GMCL1	chr2	69841503	Tau	GTEx
86	2.66E-05	0.021	1.99E-04	0.155	0.824	Hypothalamus	SNRNP27	chr2	69841503	Tau	GTEx
373	5.64E-11	9.47E-08	1.20E-05	0.019	0.981	cerebellum	GMCL1	chr2	69841503	Tau	MetaBrain
4288	4.94E-69	8.33E-66	6.27E-05	0.105	0.895	CSF-cis	ANXA4	chr2	69841503	Tau	CSF pQTL
13	0.088	0.082	0.011	0.01	0.809	Amygdala	SNRNP27	chr2	69841503	pTau	GTEx
4	0.012	0.024	0.002	0.002	0.96	Anterior_cingulate_cortex_BA24	GMCL1	chr2	69841503	pTau	GTEx
135	8.03E-06	0.009	1.30E-04	0.145	0.846	Anterior_cingulate_cortex_BA24	SNRNP27	chr2	69841503	pTau	GTEx
5	0.008	0.047	0.002	0.009	0.934	Caudate_basal_ganglia	GMCL1	chr2	69841503	pTau	GTEx
5	6.99E-04	0.04	8.32E-05	0.004	0.955	Caudate_basal_ganglia	ANXA4	chr2	69841503	pTau	GTEx
129	3.53E-06	0.004	5.80E-05	0.064	0.932	Cerebellar_Hemisphere	GMCL1	chr2	69841503	pTau	GTEx
210	3.52E-11	5.80E-08	3.11E-05	0.05	0.95	Cerebellum	GMCL1	chr2	69841503	pTau	GTEx
198	7.87E-08	1.06E-04	1.14E-04	0.153	0.847	Cerebellum	SNRNP27	chr2	69841503	pTau	GTEx
133	1.61E-08	2.56E-05	3.78E-05	0.059	0.941	Cortex	GMCL1	chr2	69841503	pTau	GTEx
17	9.18E-04	0.048	3.15E-04	0.016	0.935	Frontal_Cortex_BA9	GMCL1	chr2	69841503	pTau	GTEx
191	2.70E-10	3.63E-07	8.40E-05	0.112	0.888	Frontal_Cortex_BA9	SNRNP27	chr2	69841503	pTau	GTEx
2	0.031	0.039	0.002	0.002	0.926	Hippocampus	GMCL1	chr2	69841503	pTau	GTEx
218	1.04E-09	1.71E-06	3.95E-05	0.064	0.936	Hippocampus	SNRNP27	chr2	69841503	pTau	GTEx

23	1.98E-05	0.017	3.18E-05	0.026	0.957	Hypothalamus	GMCL1	chr2	69841503	pTau	GTEx
86	2.58E-05	0.02	1.93E-04	0.152	0.827	Hypothalamus	SNRNP27	chr2	69841503	pTau	GTEx
373	5.73E-11	9.41E-08	1.21E-05	0.019	0.981	cerebellum	GMCL1	chr2	69841503	pTau	GTEx
4288	5.29E-69	8.74E-66	6.73E-05	0.11	0.89	CSF-cis	ANXA4	chr2	69841503	pTau	CSF pQTL
18	7.74E-05	0.129	1.61E-05	0.026	0.845	Caudate_basal_ganglia	CCNO-DT	chr5	55370326	Ab42	GTEx
22	5.54E-05	0.082	2.00E-05	0.029	0.889	Putamen_basal_ganglia	MCIDAS	chr5	55370326	Ab42	GTEx
18	9.66E-05	0.132	2.00E-05	0.026	0.842	Caudate_basal_ganglia	CCNO-DT	chr5	55385798	Tau	GTEx
22	7.32E-05	0.084	2.64E-05	0.029	0.887	Putamen_basal_ganglia	MCIDAS	chr5	55385798	Tau	GTEx
336	9.30E-04	1.02E-04	0.157	0.016	0.826	cerebellum	CCNO	chr5	55385798	Tau	MetaBrain
18	1.26E-04	0.131	2.60E-05	0.026	0.843	Caudate_basal_ganglia	CCNO-DT	chr5	55385798	pTau	GTEx
22	9.47E-05	0.084	3.42E-05	0.029	0.887	Putamen_basal_ganglia	MCIDAS	chr5	55385798	pTau	GTEx
336	9.65E-04	9.41E-05	0.163	0.015	0.821	cerebellum	CCNO	chr5	55385798	pTau	MetaBrain

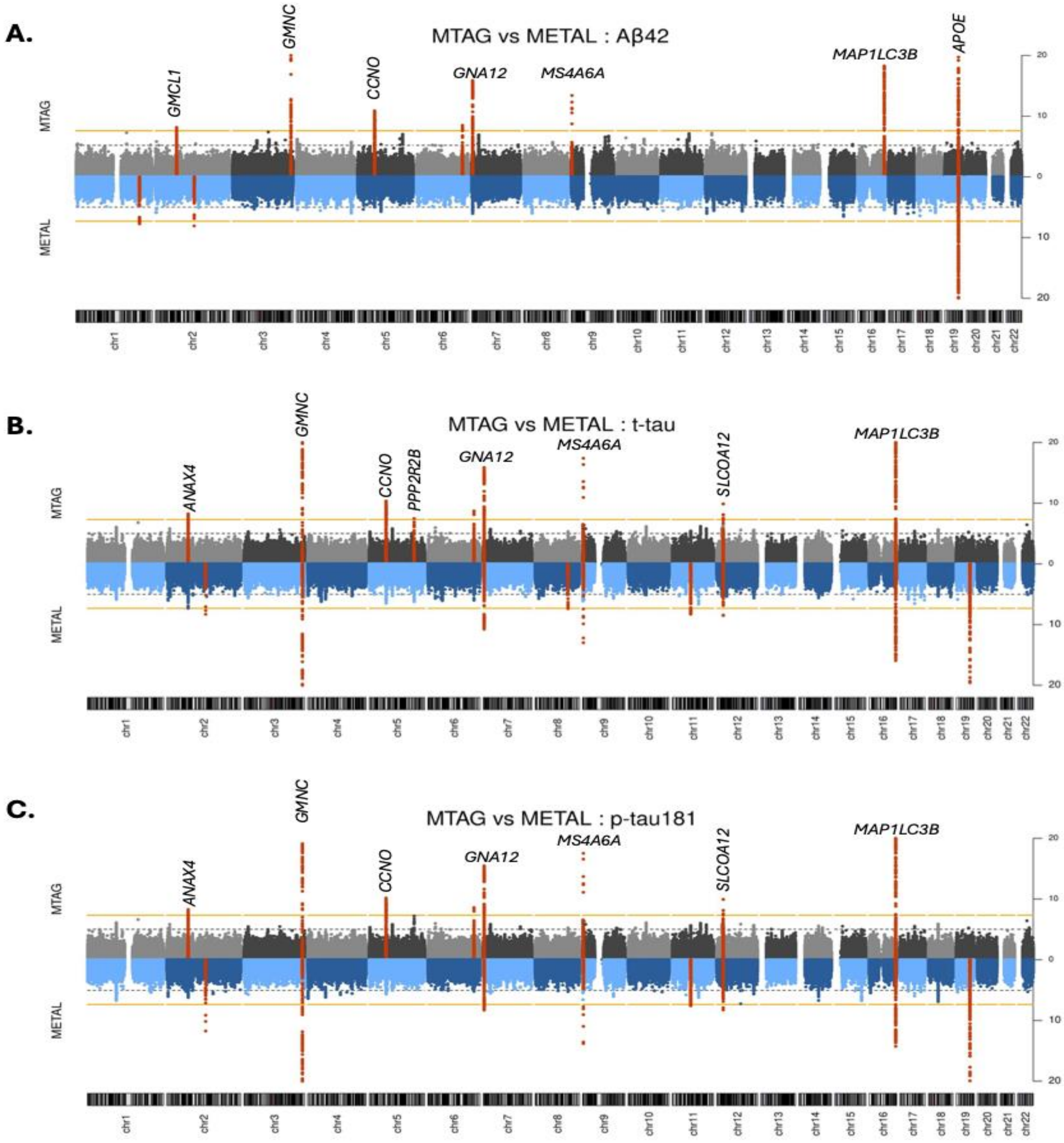

Reply Figure 4 (Supplementary Fig. 11): *Miami plot showing GWAS results from MTAG vs METAL. The orange line represents genome wide significance. Red highlighted loci are those that passed genome wide significance. Gene names labelled are genes that are either closest or found to be functional.*

We have now made changes in manuscript to add these new analyses:

Page 7, line 2: After adjusting for pleiotropy between the traits, many of the same genetic loci were identified suggesting robust association.

Page 9, line 15:
Shared genetic influences across traits using pleiotropy accounted analysis
We then analyzed all three biomarkers together using Multi-Trait Analysis of GWAS (MTAG) to identify additional signals that may have been missed by single trait GWAS[12]. Orthogonal pairwise conjunctional false discovery rate (pleioFDR) based approach was also used in combination with MTAG to identify true pleiotropic signals[5,6].

Page 9, line 29:
We identified seven genome-wide significant loci for Aβ42, nine for t-tau and eight for p-tau181 after combining results from both MTAG and pleio-FDR (Supplementary Table 3, Supplementary Fig. 11). Association of chromosome 3,7,9,12 and 16 were found for both t-tau and p-tau181. These loci were consistent with association identified in meta-analysis. Association of *APOE* with CSF Aβ42 was also identified but its association with t-tau and p-tau was not observed using MTAG. This could potentially be due to the very significant association of this locus with Aβ42 ($p=10^{-600}$ vs $p=10^{-100}$ in t-tau/p-tau181 from METAL) resulting in MTAG treating it as trait specific association or because the effect of *APOE* on t-tau and p-tau181 is not independent of Aβ42. However, previous research has shown that although part of *APOE*-t-tau/ptau181 interaction is mediated through Aβ42, some variants within the region affect tau/ptau-181 independently[7]. Associations of chromosome 7,9 and 16 with Aβ42 were novel but were same as those identified for t-tau and p-tau-181. pleio-FDR showed significant pleiotropy of these loci between Aβ42 and t-tau and ptau181 (chromosome 7 $p_{A\beta42vst\text{-}tau}=8.71\times10^{-05}$, $p_{A\beta42vspTau181}=9.14\times10^{-05}$; chromosome 9 $p_{A\beta42vst\text{-}tau}=0.031$, $p_{A\beta42vspTau181}=0.033$; chromosome 16 $p_{A\beta42vst\text{-}tau}=1.25\times10^{-04}$, $p_{A\beta42vspTau181}=1.31\times10^{-04}$; Supplementary Table 3). These findings suggest that there is some interaction between Aβ42 and t-tau/ptau181 in these loci.
In addition to these known association, there were some novel loci identified by MTAG. These included: a locus on chromosome 2 near *GMCL1,* a locus on chromosome 5 near *MTREX* and a locus on chromosome 6 near *PLEKHG1* associated with Aβ42, t-tau and p-tau181. An additional locus near *PPP2R2B* in chromosome 5 was associated with t-tau only. For loci that were unique to MTAG, we investigated the effect of the lead variant in these loci on other AD related traits using publicly available resources and conducted follow-up colocalization analysis for any loci that showed significant effect[3,4,13]. The Chromosome 6 variant associated with all three biomarkers and the chromosome 5 variant associated with t-tau only were not associated with any of the AD related traits. However, both chromosome 2 and 5 variants were nominally associated with brain amyloidosis ($p_{chr2}=0.023$; 0.016; $p_{chr5}=0.022$). Colocalization analyses were then performed for these two loci with CSF protein quantitative trait loci (pQTLs) and brain expression quantitative trait loci (eQTLs) to identify potential functional genes (Supplementary Table 4). Chromosome 2 locus colocalized with eQTLs for either *GMCL1*, *SNRNP27* or *ANXA4* depending on brain region. In addition, associations with

t-tau and p-tau181 also colocalized with CSF pQTL for *ANXA4* (Supplementary Table 4). Because *ANXA4* had colocalization evidence from both GTEx v8 Brain eQTL and CSF pQTL for tau-181 and ptau-181, we nominate the gene as most likely functional gene for the locus. *ANAX* family of genes have been shown to increase in brain of AD patients and play a role in Aβ clearance and also contribute to the localization of tau in the axon[8,9]. We did not observe colocalization of Aβ42 association with pQTL for *ANXA4* (PP.H4=0.536), thus *GMCL1* is most likely the functional for Aβ42 association at chromosome 2 based on evidence of colocalization from GTEx v8 Brain eQTL and MetaBrain. Overexpression of *GMCL1* has been linked to certain type of cancers however there is no direct evidence linking it to AD[10]. In light of these findings, we hypothesize that the chromosome 2 locus has trait specific regulatory mechanisms. Similarly, chromosome 5 locus colocalized with brain eQTLs for *CCNO* and *MCIDAS* gene. In t-tau and ptau-181, colocalization was observed in both GTEx v8 Brain eQTL and MetaBrain eQTL but in Aβ42 this was only found in GTEx v8 Brain eQTL. *CCNO* had evidence from both GTEx and MetaBrain; thus, *CCNO* is likely the functional of the two genes. The *CCNO* gene has been reported to control other genes in AKT signaling cascade which is an important disease mechanism in AD[9,11].
Overall, after accounting for pleiotropy between the biomarkers, we observed signals previously identified in our meta-analysis as well as few novel signals that point to additional disease mechanism.

Page 16, line 19: As mentioned in earlier sections, ANAX4/ GMCL1 and CCNO loci, identified in pleiotropy adjusted analysis, were nominally associated with brain amyloidosis (pchr2=0.023; 0.016; pchr5=0.022).

Page 21, line 2:
Majority of these loci were also observed in cross-trait comparison accounting for pleiotropy between the biomarkers.

Page 21, line 15: Although we identified APOE to be associated with all three biomarkers in our meta-analysis, cross- trait analysis showed it to be Aβ42 specific. However, previous research has shown that although part of APOE-t-tau/ptau181 interaction is mediated through Aβ42, some variants within the region affect tau/ptau-181 independently.

Page 22 , line 24: The *GNA12*, *MS4A6A* and *MAP1LC3B* loci were also found to be shared with Aβ42 via cross-trait analysis, likely pointing to an interaction between the biomarker at these loci.

Page 23, line 31: In addition to all these, other potential disease mechanisms, highlighted by cross-trait analysis, included aggregation of tau in axons and disturbances in the AKT signaling cascade mediated by ANAX4 and CCNO gene20,22

Page 29, line 5:
Cross- trait analysis and pleiotropy check:

To further identify shared and trait specific signals, we applied Multi-Trait Analysis of GWAS (MTAG) using publicly available MTAG tool[14]. Meta-analysis summary statistics for each trait was used as input. rsIDs were used as SNP identifiers for the analysis. Because, MTAG assumes homogenous effects of genetic variants across trait, which is not the case in our biomarkers, we employed an orthogonal pairwise conjunctional false discovery rate analysis using previously established apprach[15,16]. A signal was considered true positive if it met two sets of criteria: (1) it had to pass genome wide significance in MTAG and (2) pass conjunctional FDR p<0.05 for pleiotropy with either of the remaining two biomarker.

Tables and figures presented above have been added as supplementary figure and table now.

2. **The genetic correlations with AD risk are surprisingly low values but are significant, for example CSF A42 with AD risk rg=-0.013, pFDR=2.6710-38. Could the authors comment on this, validate with additional genetic correlation approaches, or check for typos.**

Response: The coefficients presented here were the genetic covariance which is an unstandardized form of genetic correlation. Because these values are unstandardized, they are affected by scale of estimates in underlying GWAS summary statistics. However, when sample size is large, as in case of AD risk GWAS, even small covariance can be estimated with high precision by GNOVA[14]. In comparison, the genetic correlation for these same set of phenotypes is -0.69 using GNOVA and -0.4454 using LDSC. To confirm our covariance estimates, we have now run covariance test with LDSC for all phenotypes. Particularly for AD risk the covariance estimate from LDSC were -0.019, 0.014 and 0.0139 for Aβ42, t-tau and p-tau181 respectively. The overall correlation between the tools was 0.975 for Aβ42, 0.85 for t-tau and 0.816 for p-tau181.

We have now made the following changes to the manuscript:

Page 17, line 1: As additional check, these estimates were also confirmed using Linkage Disequilibrium Score Regression (LDSC; Supplementary Fig. 10).

Page 17, line 23: We observed significant and strong correlation between estimates from GNOVA and LDSC with coefficients being 0.975 ($p=2.9\times10^{-13}$), 0.85 ($p=2.05\times10^{-6}$), and 0.816 ($p=1.14\times10^{-05}$) for Aβ42, t-tau and p-tau181 respectively (Supplementary Fig. 15). When these comparisons were repeated without including the *APOE* gene region similar strong and significant correlation were observed ($r_{\beta42}=0.86$, $p_{A\beta42}=1.04\times10^{-06}$; $r_{t\text{-}tau}=0.741$, $p_{t\text{-}tau}=1.83\times10^{-04}$; $r_{p\text{-}tau181}=0.701$ $p_{p\text{-}tau181}=5.77\times10^{-04}$)

Page 32, line 11: Findings from GNOVA were further verified using complimentary LDSC tool.

The figure below has now been included as **Supplementary Fig. 17.**

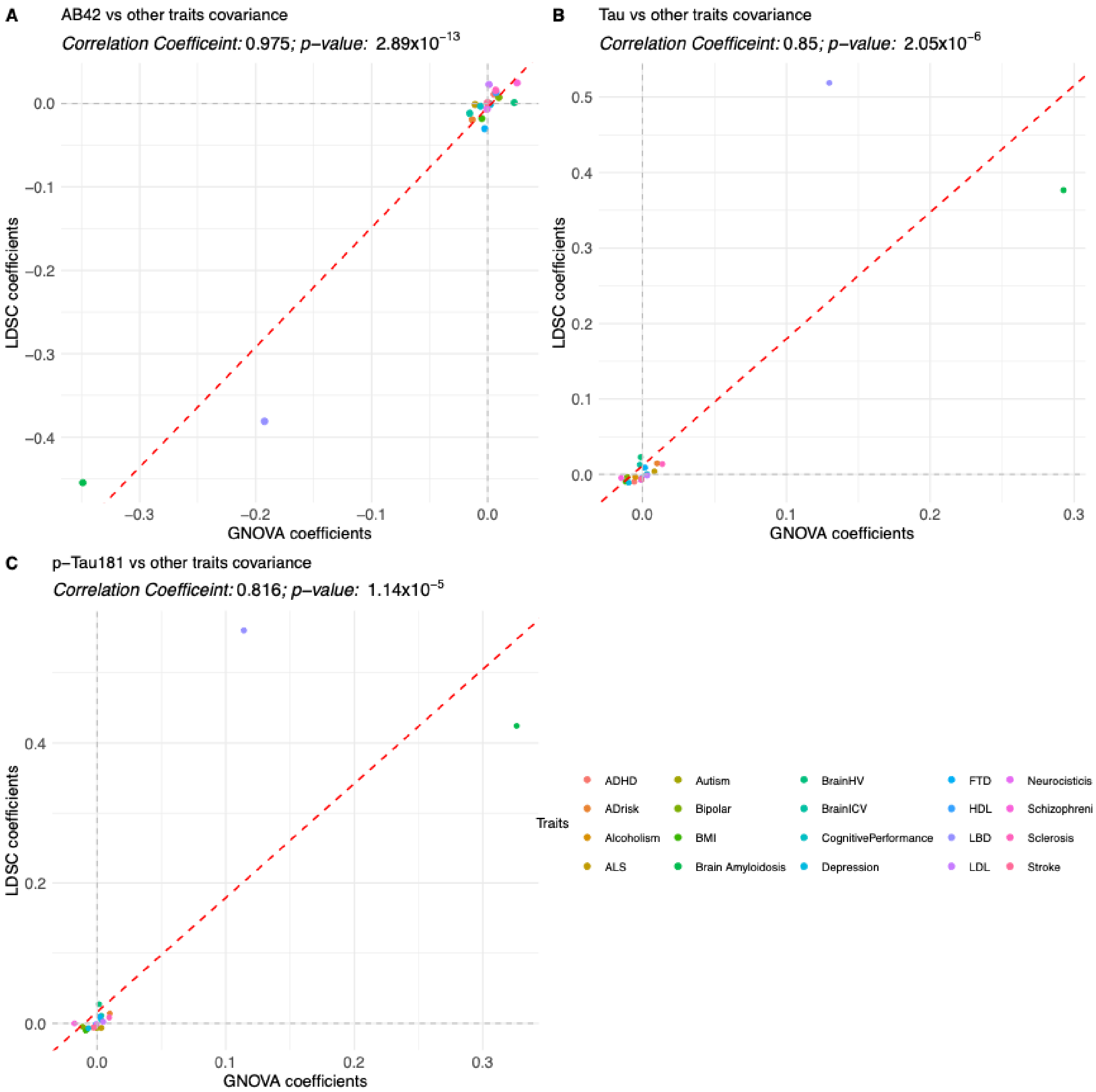

Reply Figure 5 (Supplementary Fig.17): *Scatterplot plot showing correlation between genetic covariance estimates from GNOVA and LDSC. Each dot represents a trait. X-axis shows estimates from GNOVA and Y-axis shows estimates from LDSC.* ***(A)*** *Correlation of covariance estimates between traits and Aβ42 (0.975; p:2.89×10^{-13}).* ***(B)*** *Correlation of covariance estimates between traits and t-tau (0.85; p:2.05×10^{-06}).* ***(C)*** *Correlation of covariance estimates between traits and Aβ42 (0.816; p:1.14×10^{-05}).*

3. **Please perform a stratified analysis with AD or control groups and SNP*diagnosis interaction to determine if genetic effects are impacted by diagnostic status. The authors found variants not only associated with CSF level of the three biomarkers but also showed significant association with AD risk, disease progression and/or brain amyloidosis. Does this also occur if the data are stratified by AD status or just reflecting the relationship between biomarker measurements and AD status?**

Response: Please see reply to reviewer 2, question 4 for a detailed point by point answer to this question.

4. **Could the author show the statistics of variants identified from previous studies, even if those are not significant in the current study.**

Response: Previous GWAS study for our phenotype of interest identified 2 significant genomic loci for Aβ42 and four for p-tau181[2]. All these previously identified genomic loci have also been replicated in our study. We have now added a comparison table showing variants identified in the previous study and their estimates from current study as supplementary table 1. Although, the findings for t-tau have been mentioned to have been highly correlated with-ptau181 in the previous study, summary statistics is only available for Aβ42 and p-tau181 publicly.

We have now added the requested information to supplementary file 1 and made following changes in manuscript:

Page 7, line 24: The rs10779336[T] variant in the CR1 gene region was associated with CSF Aβ42 levels and was in high linkage disequilibrium (LD) with the lead variant from Jansen et. al. study (rs4844610[A], r2=0.95, Supplementary Table 1,2).

Page 8, line 4: Lead variants reported in the previous Jansen *et. al.*[2] study all showed a consistent direction of effect and were significant in our analysis (Supplementary Table 2).

Supplementary Table 2:

Supplementary Table 2: Table showing summary statistics of variants identified in previous GWAS for the phenotypes of interest in current European meta-analysis

					Previous GWAS		Current Meta-analysis	
**rsID**	**Chr**	**Pos**	**Phenotype**	**A1**	**Z**	**p-value**	**Beta**	**P-value**
rs4844610	1	207,629,207	Aβ42	A	−6.01	1.84E−09	-0.0707	4.80E-08
rs429358	19	44,908,684	Aβ42	C	-41.68	1.00E−379	-0.5889	1.90E-674
rs744373	2	127,137,039	p-tau181	G	6.15	7.88E−10	0.0677	8.62E-10
rs9877502	3	190,951,729	p-tau181	A	12.66	9.65E−37	0.1516	1.82E-47
rs4843559	16	87,191,825	p-tau181	G	7.47	8.03E−14	0.0773	5.98E-13
rs429358	19	44,908,684	p-tau181	C	20.65	9.59E−95	0.3184	3.27E-174

5. **Could the author show similar forest plots like Supplementary Figures 1-7 for non-European ancestries.**

Response: Unlike European meta-analysis, non-European samples used in this study were only available as part of the joint dataset, obtained by combining phenotypes as continuous measure using z-scores. Thus, effect sizes for this analysis consists of a single, pooled measure and separate estimates by study or cohort is not available for the samples. Thus, forest plot is not applicable in context of non-European ancestry GWAS.

6. **Although the author acknowledged "Finally, future research to evaluate genetic correlations between A42 and t-tau/p-tau181 and identify CSF A42-independent genetic signals associated with CSF t-tau /p-tau181 will be needed.", please estimate the phenotypic and genetic correlations of these CSF biomarkers using this dataset.**

Response: As per reviewer's request we have now calculated phenotypic correlation between the biomarkers using all available individual level raw data and genotypic correlation using meta-analysis summary statistics. Because standardized scores were calculated for each cohort independently prior to joint analysis, phenotypic correlation were checked within each cohort. Individual level data were unavailable for studies included in meta-analysis due to data sharing restrictions. Genetic correlations were tested using LDSC tool.

We observed significant negative correlation of Aβ42 with both t-tau and p-tau. The median phenotypic correlation between the traits pair were -0.28 and -0.22 respectively. This is consistent with expected direction of these biomarker changes in CSF in disease condition. Similarly, median phenotypic correlation between t-tau and p-tau181 were 0.91 with an average of 0.83.

Genotypic correlation between Aβ42 and t-tau/p-tau were not significant ($p_{A\beta42\ vs\ t\text{-}tau}=0.25$ and $p_{A\beta42\ vs\ p\text{-}tau181}=0.27$). Correlation measures the sum total of shared architecture across the entire genome; thus, we observed this non-significant correlation despite our pleiotropy analysis showing that multiple pleiotropic signals between the traits. Correlation between t-tau and p-tau181 was highly significant ($p_{t\text{-}tau\ vs\ p\text{-}tau181}=6.99\times10^{-303}$). Correlation coefficient was out of bounds at 1.07. This is a known behavior of LDSC when there is a high sample overlap between traits and an expected high correlation.

Overall, we observed significant phenotypic correlations between the traits and significant genotypic correlation between t-tau and p-tau181.

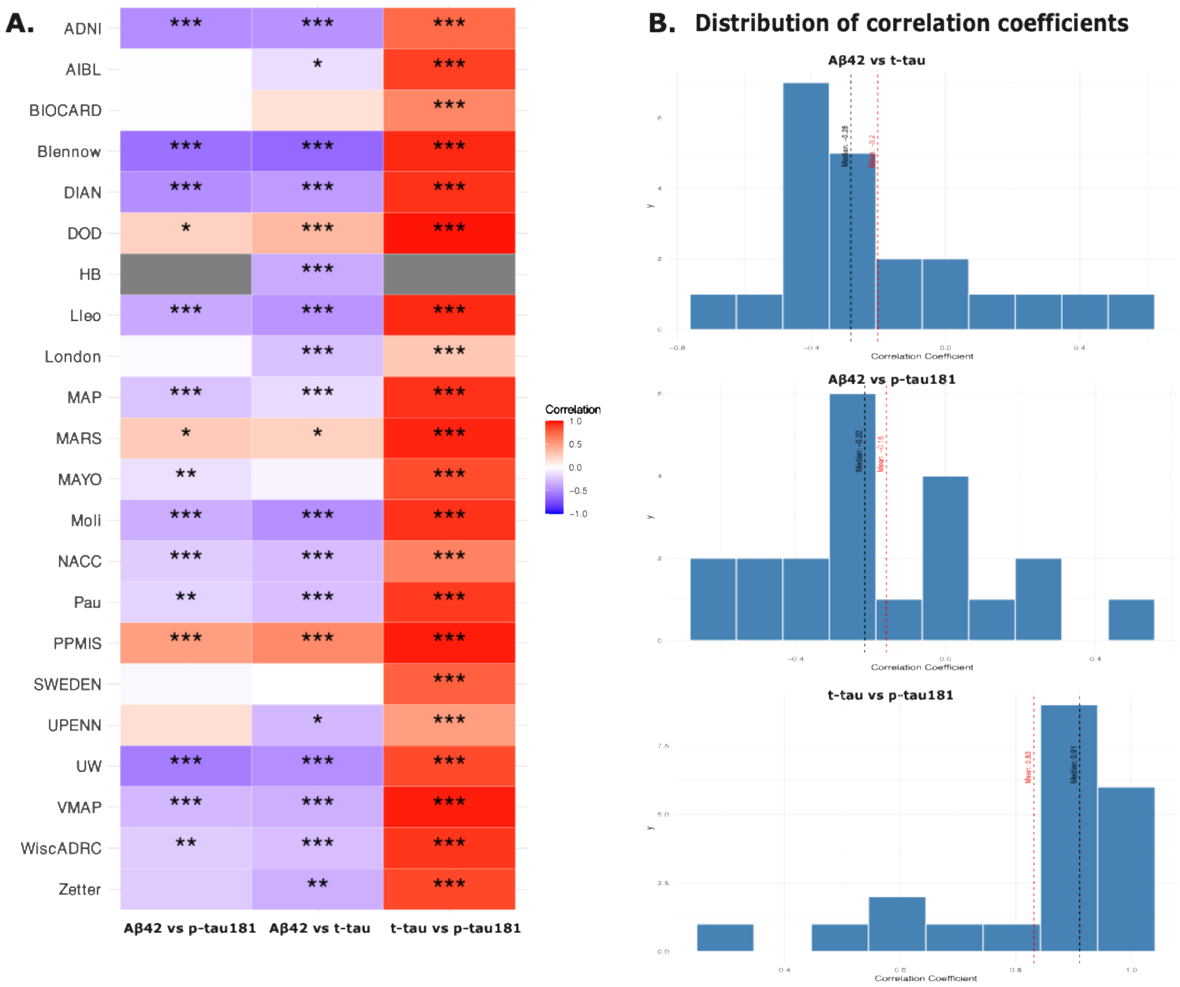

Reply Figure 6 (Supplementary Figure 10) *:Plot showing phenotypic correlation between biomarkers across different cohorts for which individual level data was available. (A) Heatmap showing correlation per each cohort. "*" represents strength of correlation. "***": p<0.001; "**": p<0.01; "*": p<0.05. . Grey tiles represent no available data for comparison (B) Histograms showing distribution of correlation coefficients in each comparison. Black dotted line shows the median and red dotted line represents the mean.*

We have now made following changes in the manuscript to include these comparisons:

Page 9, line 21: Prior to pleiotropy test, analysis of genetic correlation between the traits showed a highly significant genetic correlation between t-tau and p-tau181 ($p=6.99\times10^{-303}$), however, correlation of these two traits with Aβ42 was not significant ($p_{A\beta42\ vs\ t\text{-}tau}=0.25$ and $p_{A\beta42\ vs\ p\text{-}tau181}=0.27$).

Page 9, line 24: Phenotypic correlation, assessed using available individual level data, were in expected direction with a significant negative correlation of Aβ42 with both t-tau and p-tau (Supplementary Fig. 10). The median phenotypic correlation between the traits pair were -0.28 and -0.22 respectively. Similarly, we observed significant positive phenotypic correlation between t-tau and p-tau181 with a median correlation of 0.91 and an average of 0.83 (Supplementary Fig. 10).

The plot above has been now added as Supplementary figure 10.

7. **Uncorrected P-value < 0.05 for pathway enrichment analysis may be too lenient. Some reported "significant" pathways have only one gene overlap, which are often difficult to believe. Please add gene counts in the pathway DB.**

Response: Our pathway analysis is exploratory in nature and thus we applied a lenient threshold to avoid exclusion of potentially relevant signals. Despite having used a lenient threshold the top pathways associated with t-tau and p-tau that have been highlighted in plots and in text passed FDR p<0.05 threshold. However, to maintain consistency across pathway analysis, we presented the lenient threshold throughout. The limited gene overlap seen in some pathways could be a function of the limited sets of genes available as input ($N_{A\beta42}$=55; N_{Tau}=107; $N_{p\text{-}tau181}$=85) or a similarly limited background gene set associated with those pathways. To ensure transparency and reproducibility we reported any pathway that passed our relatively lenient threshold. As per reviewer's suggestion we have now added the background gene to the supplementary table 18.

8. **Because the authors have separately measured Abeta and Tau CSF biomarkers, it seems possible to use Mendelian Randomization to address some important causal questions in the field. That is does Abeta pathology lead to Tau pathology? Similarly, does Abeta pathology causally contribute to AD risk? Does Tau pathology causally contribute to AD risk? Given the strong debates in the field currently, these MR analyses seem highly relevant.**

Response: Our pleiotropy analyses show that the three biomarkers analyzed here are highly pleiotropic in nature. The core assumptions of Mendelian randomization (MR), i.e. variants influence outcome through exposure of interest, is not valid in our case as same variants may be influencing t-tau and p-tau independent of Aβ42. Thus, validity of any MR results would be questionable at best.

We have now performed MR of Aβ42, t-tau and p-tau181 with AD risk[3] as outcome. MR was performed using "TwoSampleMR" package and traits were analyzed using five methods; MR Egger, Weighted Median, Inverse Variance Weighted (IVW), Simple Mode, and Weighted Mode. All five methods identified a significant negative association between Aβ42 and AD risk indicating that higher levels of Aβ42 are causally associated with a reduced risk of Alzheimer's disease (see table below). Specifically, the IVW method demonstrated an effect size of -1.806 and a highly significant p-value ($p = 1.14\times10^{-16}$). On the other hand, t-tau and p-tau181 did not have any significant association with AD risk in any of the methods tested. Additionally, pleiotropy check done as part of the MR pipeline did not show any significant pleiotropy between the biomarkers and AD risk ($p_{A\beta42}$=0.379; p_{Tau}=0.380; $p_{p\text{-}tau181}$=0.520) showing that these results are not biased due to genetic pleiotropy. Overall, our results from MR indicate that while CSF Aβ42 is directly linked to AD risk, CSF t-tau and p-tau181 do not directly influence risk of AD disease.

We have now made following changes in main manuscript to reflect the new analysis done.

Page 18, line 4:

CSF Aβ42 is causally associated with increased risk of AD

Many of our lead variants from meta-analysis were also significantly associated with AD risk, so we sought to investigate causal link between the three biomarkers and disease risk.

Mendelian randomization (MR) analyses were performed using the biomarkers as exposure and AD risk[3] as outcome variable. MR was performed using five methods; MR Egger, Weighted Median, Inverse Variance Weighted (IVW), Simple Mode, and Weighted Mode. All five methods identified a significant negative association between Aβ42 and AD risk indicating that higher levels of Aβ42 are causally associated with a reduced risk of Alzheimer's disease (Supplementary Table 15). Specifically, the IVW method demonstrated an effect size of -1.806 and a highly significant p-value ($p = 1.14\times10^{-16}$). On the other hand, t-tau and p-tau181 did not have any significant association with AD risk in any of the methods tested. Additionally, pleiotropy check done as part of the MR pipeline did not show any significant pleiotropy between the biomarkers and AD risk ($p_{A\beta42}$=0.379; p_{Tau}=0.380; $p_{p\text{-}tau181}$=0.520) showing that these results are not biased due to genetic pleiotropy. Overall, our results from MR indicate that while CSF Aβ42 is directly linked to AD risk, CSF t-tau and p-tau181 do not directly influence risk of AD disease.

Page 21, line 8: In fact, evidence from MR analysis showed that CSF Aβ42 is associated with risk of AD.

Page 28, line 5:

We used "TwoSampleMR" package in R to perform Mendelian Randomization (MR) between Aβ42, pTau, tau, and AD risk[3,15]. For each biomarker, variants were mapped to rsIDs and imported using read_exposure_data(). Variants with p-values < 5×10-8 were selected for each and clumped using default parameters using clump_data().AD riskGWAS summary statistics were extracted for overlapping variants (based on rsID) using read_outcome_data() and effects were harmonized across exposure and outcome using harmonise_data(). MR was performed using mr() using five different methods: MR Egger, Weighted Median, Inverse Variance Weighted, Simple Mode, and Weighted Mode, with inverse-variance weighted statistics being prioritized. To test for horizontal pleiotropy, the significance of the MR-Egger intercept was calculated for each comparison.

Following table has now been added as **supplementary table 16.**

Supplementary Table 16: Table showing MR estimates for the exposures on risk of AD

Exposure Variable	Outcome variable	method	nsnp	b	se	pval
Aβ42	AD risk	MR Egger	5	-1.477	0.386	3.14E-02
Aβ42	AD risk	Weighted median	5	-1.804	0.137	2.22E-39
Aβ42	AD risk	Inverse variance weighted	5	-1.806	0.218	1.14E-16
Aβ42	AD risk	Simple mode	5	-1.764	0.204	9.75E-04
Aβ42	AD risk	Weighted mode	5	-1.798	0.186	6.43E-04
T-tau	AD risk	MR Egger	9	-0.360	0.825	6.76E-01
T-tau	AD risk	Weighted median	9	0.061	0.050	2.26E-01
T-tau	AD risk	Inverse variance weighted	9	0.356	0.286	2.13E-01
T-tau	AD risk	Simple mode	9	0.019	0.057	7.51E-01
T-tau	AD risk	Weighted mode	9	0.028	0.044	5.36E-01
p-tau181	AD risk	MR Egger	8	-0.197	0.952	8.43E-01
p-tau181	AD risk	Weighted median	8	0.066	0.049	1.77E-01
p-tau181	AD risk	Inverse variance weighted	8	0.411	0.311	1.86E-01
p-tau181	AD risk	Simple mode	8	-0.032	0.058	6.01E-01
p-tau181	AD risk	Weighted mode	8	0.020	0.043	6.56E-01

9. **It is commonly thought that effect sizes for endophenotypes are higher than disease diagnosis. Please compare effect size distributions for these endophenotypes versus AD diseases status, using for example this technique: https://www.nature.com/articles/s41588-018-0193-x.**

Response:

To address reviewer's question on whether endophenotypes have higher effect size compared to diagnosis based GWAS. We ran identical GWAS models in same set of samples (N=5,128 [AD=2,365; CO=2,763]) to ensure that the statistics are free of methodological differences. Because our biomarkers were z-scored, we calculated t-statistics for each regression coefficient to make them directly comparable. Overall, the effect size across endophenotype and diagnosis appear to be highly correlated (Reply figure 7). However, we also observed that for variants associated with genes that are known to directly influence the endophenotypes of interest, these associations showed up to 20-fold increase in magnitude of effect when compared to disease GWAS. For example, variant in *APOE* gene which is a direct regulator of Aβ42 levels had a z-score of -29.587 compared to 15.589 in diagnosis-based analysis with a p-value in order of 10^{-177} compared to 10^{-54}. Similarly, *MS4A6A* variant had 1.5 times higher effect size in t-tau and p-tau181 compared to diagnosis. *MAP1LC3B* variant which is associated with t-tau and p-tau181 had almost 20 times higher effect on these endophenotypes compared to diagnosis status. So, although effect sizes were highly correlated and comparable between endophenotypes and diagnosis status, we found that depending on involvement of variants in mechanism relevant to the endophenotypes, these effects are significantly increased. This highlights the potential of endophenotype based study to better capture the biological underpinnings of complex disease more robustly which would otherwise be diluted when focusing on diagnosis status only.

Supplementary Table 6: Table showing Z statistics and p-value for each endophenotype and disease diagnosis GWAS

ID	rsID	Nominated gene	Z_ab	p_AB	Z_tau	p_Tau	Z_pTau	p_pTau	Z_diagnosis	p_diagnosis
chr1:207625371:T:G	rs10779336	CR1	-2.580	0.010	3.038	0.002	3.753	1.77E-04	3.505	4.56E-04
chr2:127135234:C:T	rs6733839	BIN1	-2.840	0.005	2.153	0.031	3.304	0.001	2.171	0.030
chr7:2761908:G:A	rs798490	GNA12	-1.807	0.071	-2.775	0.006	-1.003	0.316	-0.999	0.318
chr7:2718675:A:G	rs798560	GNA12	-1.999	0.046	-2.562	0.010	-1.359	0.174	-1.018	0.308
chr11:60207692:G:A	rs7928895	MS4A6A	0.698	0.486	-3.879	1.06E-04	-3.520	4.35E-04	-2.431	0.015
chr11:60254475:G:A	rs1582763	MS4A6A	0.913	0.361	-3.910	9.34E-05	-3.928	8.68E-05	-2.787	0.005
chr16:87199910:G:A	rs4843552	MAP1LC3B	1.568	0.117	5.095	3.62E-07	4.321	1.59E-05	0.259	0.796
chr19:44908684:T:C	rs429358	APOE	-29.587	1.51E-177	13.744	3.26E-42	15.551	3.40E-53	15.489	4.14E-54

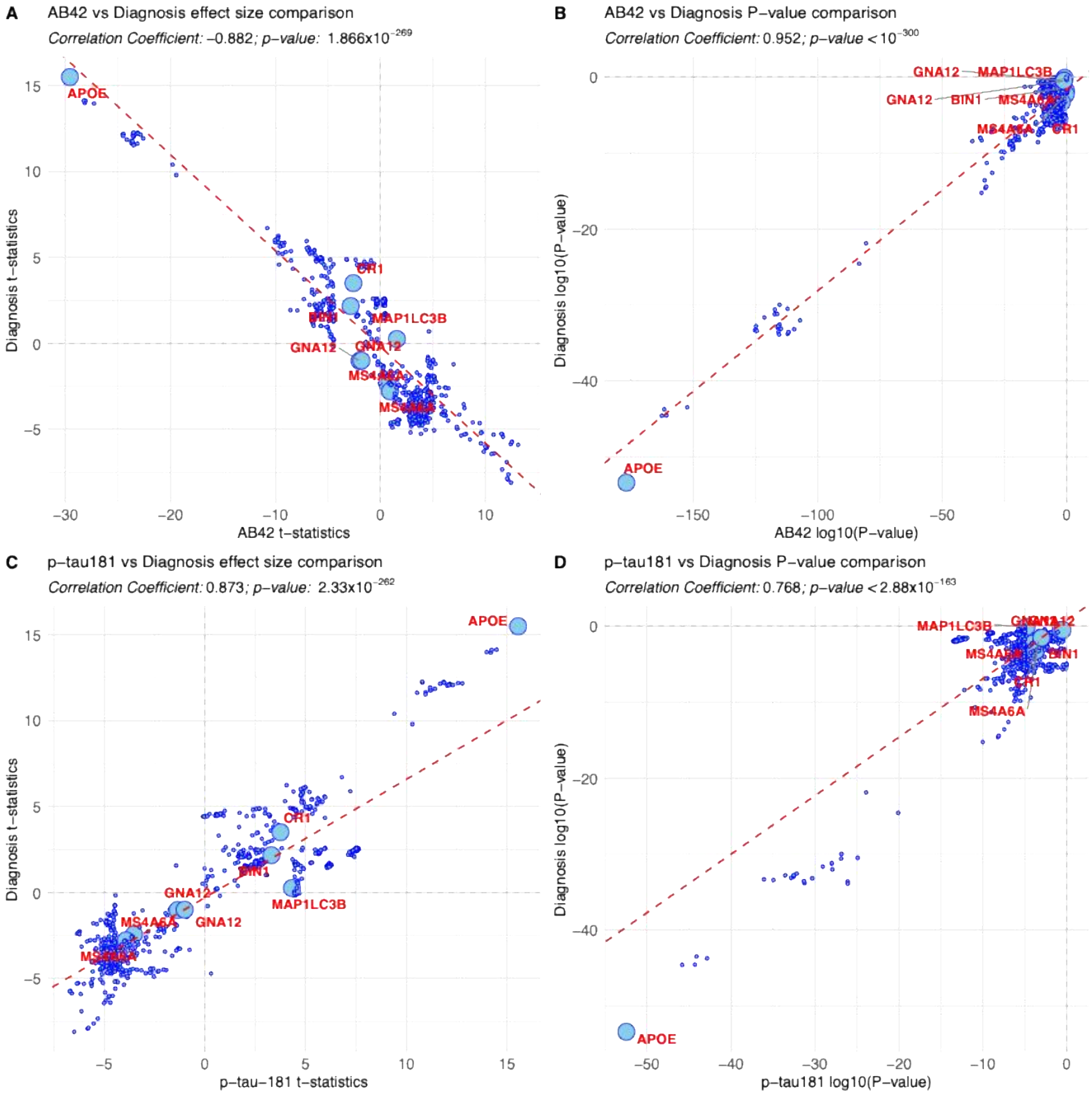

Reply Figure 7 (Supplementary Figure16): *Scatter plot showing correlation between GWAS coefficients.* ***(A)*** *Aβ42 and Diagnosis status t-statistics* ***(B)*** *Aβ42 and Diagnosis status log10 (p-value)* ***(C)*** *p-tau181 and Diagnosis status t-statistics* ***(D)*** *p-tau181 and Diagnosis status log10 (p-value) . Labelled dots show lead variants from meta-analysis that were associated with AD risk. Variants that were nominally significant in either analysis or one of the variants associated with AD risk in meta-analysis was used for comparison.*

We have now made following changes in the manuscript

Page 13, line1:

Comparison between endophenotypes versus disease status as phenotype of interest

Because endophenotypes are thought to have a higher effect size compared to disease status, we also performed sensitivity analysis by running identical GWAS model in same

set of samples (N=5,128 [AD=2,365; CO=2,763]) to ensure that the statistics are free of methodological differences. Because our biomarkers were z-scored, we calculated t-statistics for each regression coefficient to make them directly comparable. Overall, the effect size across endophenotype and diagnosis appear to be highly correlated (Supplementary Fig. 16). However, we also observed that for variants associated with genes that are known to directly influence the endophenotypes, these association showed up to 20-fold increase in magnitude of effect when compared to disease GWAS (Supplementary Table 6). For example, variant in *APOE* gene which is a direct regulator of Aβ42 levels had a z-score of -29.587 compared to 15.589 in diagnosis-based analysis with a p-value in order of 10^{-177} compared to 10^{-54}. Similarly, *MS4A6A* variant had 1.5 times higher effect size in t-tau and p-tau181 compared to diagnosis. *MAP1LC3B* variant which is associated with t-tau and p-tau181 had almost 20 times higher effect on these endophenotypes compared to diagnosis status. So, although effect sizes were highly correlated and comparable between endophenotypes and diagnosis status, we found that depending on involvement of variants in mechanism relevant to the endophenotypes, these effects are significantly increased. This highlights the potential of endophenotype based study to capture the biological underpinnings of complex disease more robustly which would otherwise be diluted when focusing on diagnosis status only.

Page 21, line 1: Compared to diagnosis based GWAS, we found that endophenotype based study captures the biology of the disease more robustly.

The figure and tables above have been added to supplementary materials.

10. The chr17 inversion locus contains MAPT, and some previous evidence shows that it does effect MAPT expression, but interestingly the locus does not show up in the GWAS for CSF tau measurements. I wonder if the authors could comment on this and show the locus to see if it has some nominal level of significance for tau phenotypes.

Response: We have now visualized the p-values of all available variants in the 17q21.31 cytogenic region that contains both H1 and H2 *MAPT* haplotype. Further, *MAPT* gene region (chr17:45894554-46028334; ±1Mb) was further evaluated using locus zoom. The most significant variant associated with t-tau in this region was chr17:43551454:A:G (BETA=-0.114, $p=3.94\times10^{-04}$) followed by variant chr17:45964621:T:C (BETA=0.053, $p=4.20\times10^{-04}$). None of the variants passed the genome wide ($<5\times10^{-08}$) or the suggestive ($<1\times10^{-04}$) significance threshold. Both these variants were also one of the most significant variants associated with p-tau181within the cytogenic region ($Beta_{chr17:43551454:A}$=-0.129, $p_{chr17:43551454:A}=1.13\times10^{-04}$; $Beta_{chr17:45964621:T:C}$=0.053, $p_{chr17:45964621:T:C}=4.16\times10^{-04}$) with two additional variant chr17:42877528:G:A (BETA=0.111, $p=1.46\times10^{-04}$) and chr17:42836985:A:G (BETA=-0.109, $p=1.83\times10^{-04}$) showing similar levels of significance. Variant chr17:46810843:G:A, just outside this cytogenic region, had $p=1.58\times10^{-05}$ for its association with p-tau181 and was just below the nominal significance.

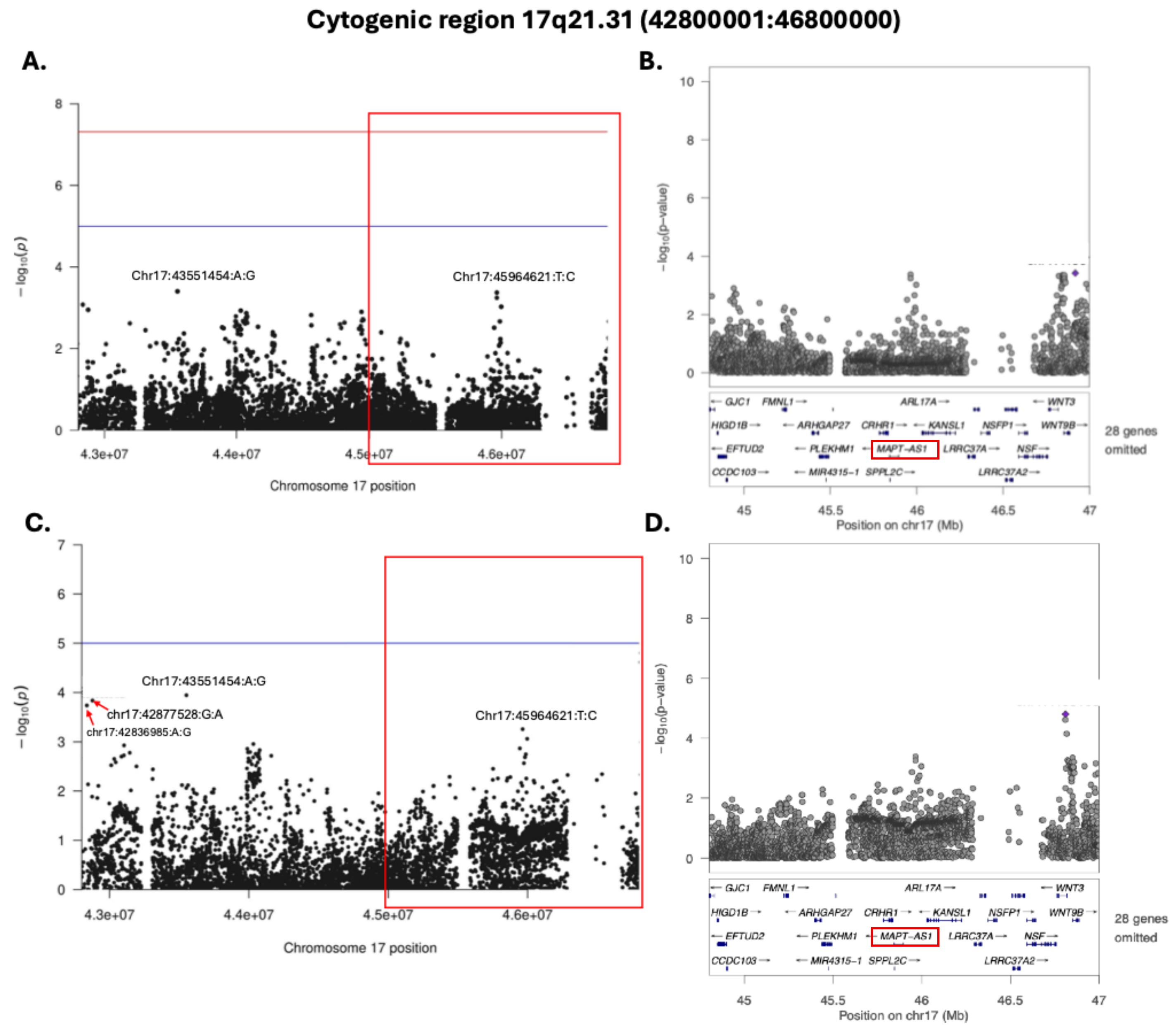

Reply Figure 8 (Supplementary Figure 9): *Plot showing association of chromosome 17 inversion locus containing both MAPT haplotypes with t-tau and p-tau. (A-B) Manhattan and locus zoom plot showing association of the region with t-tau. No genome or suggestive significant association observed. Locus zoom covers the area within the red boundary shown in Manhattan plot. (C-D) Manhattan and locus zoom plot showing association of the region with t-tau. No genome or suggestive significant association observed. Locus zoom covers the area within the red boundary shown in Manhattan plot.*

We have now added this figure as supplementary figure and made following comments in manuscript:

Page 9, line 11: The chromosome 17 inversion locus that contains *MAPT* gene, that encodes tau, did not have any association with tau or ptau-181 (Supplementary Fig. 9).

11. **Cohorts in Supplementary Table 16 look different from cohorts with summary statistics. Is this correct? And the cohorts are not always imputed to TOPMed GRCh38 as described in the methods. Please clarify.**

Response: Cohort 1-22 (ADNI to Zetter) presented in table 1 were analyzed as one singular study by combining the biomarker values using standardized z-scores, calculated in each cohort individually after Quality Control (QC). The genotype data were also QCed individually and merged as one. These analyses were performed at Washington University in St. Louis Charles F. and Joanne Knight Alzheimer Disease Research Center (Knight ADRC). Thus, in supplementary table 16 (current supplementary table 17) the genotype QC detail has been presented as one as “Knight ADRC”.

Summary statistics which were provided as GRCh37 were lifted over to GRCh38 prior to meta-analysis.

We have now included clarifying statements in the manuscript:

Page 25, line 30: A total of 6,785 participants across 22 studies were combined into a single dataset using a z-score based data standardization (mean 0 and variance 1 within each cohort) and analyzed at Washington university in St. Louis Charles F. and Joanne Knight Alzheimer Disease Research Center (Knight ADRC).

Page 27, line 20: For the 22 studies, analyzed at Knight ADRC, that were included in the joint analysis, QC was performed in each study individually and then combined for downstream analysis.

Page 27, line 27: When summary statistics file, from contributing sites, were provided in GRCh37 genomic coordinates, the standalone Linux-based UCSC LiftOver program was used to lift them over to GRCh38 prior to analysis.

12. How were ancestries defined? Please provide a threshold
Response: Ancestries were based on genetic principal component 1 and 2 calculated using samples from 1000 genomes project as anchor. Based on visual inspection of PCA plot an PC1<0 and PC2>-0.003 were used to select European samples. The population stratification PCA plot is presented as supplementary figure 13 and the cut-off have been added to the plot now.

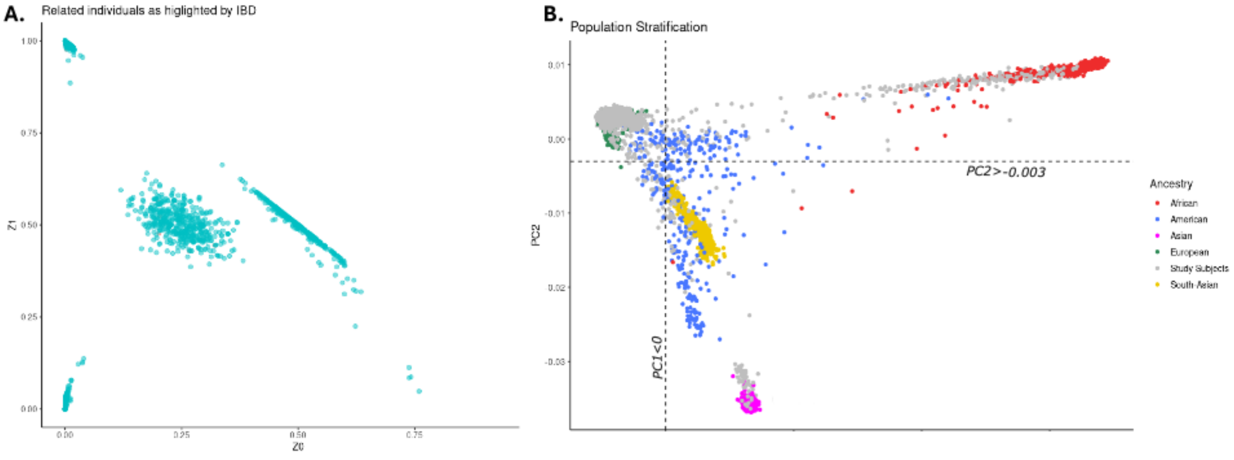

***Reply Figure 9 (Supplementary Fig.19)**: **(A)** Identity by descent (IBD) plot showing probability of sharing no allele (Z0; x-axis) and one allele (Z1; y-axis) in related individuals. "0" Z0 and Z1 value shows exact duplicates whereas "1" Z1 shows parent-offspring pairs. Only individuals with pihat <0.2 were kept. **(B)** Genetic principal component analysis (PCA) plot of study samples with 1000 Genomes samples as anchor. Only European ancestry population (PC1<0; PC2>-0.003) population were kept.*

13. Line 408: p=5.5247e-16 -> use the same format as other stats; there are also something like ×-08 or 10-8 (number varies)
Response: We thank the reviewer for pointing out these inconsistencies. We have now proofread the manuscript and ensured a consistent numbering format throughout.

14. Line 737: RCh38 -> GRCh38
Response: We have now edited the manuscript and reformatted the typo.

Page 27, line 17: Following this, imputation was performed using the TOPMed Imputation Server (https://imputation.biodatacatalyst.nhlbi.nih.gov/; GRCh38 Version R2 reference panel).

15. Line 746 and Supplemental Table 16: MAF of 0.05 % looks too low, is that 5% (=0.05) or indeed 0.0005?
Response: We used a MAF threshold of 0.0005 (i.e. 0.05%) to include rare variants like TREM2 p.R47H variants which have these level of allele frequency[16].

16. Line 782: toGRCh 37 -> to GRCh37

Response: We have now edited the manuscript and reformatted the typo.

Page 29, line 18: variant positions were lifted over from GRCh38 to GRCh37 at the time of this analysis.

17. **Check reference to Table S2 in Study description in Additional Material European Prevention of Alzheimer's Dementia**

Response: We have now corrected the typo to redirect to the correct supplementary table.

1. Bulik-Sullivan BK, Loh PR, Finucane HK, et al. LD Score regression distinguishes confounding from polygenicity in genome-wide association studies. *Nat Genet*. Mar 2015;47(3):291-5. doi:10.1038/ng.3211
2. IE J, SJ vdL, D G-F, et al. Genome-wide meta-analysis for Alzheimer's disease cerebrospinal fluid biomarkers - PubMed. *Acta neuropathologica*. 2022 Nov;144(5)doi:10.1007/s00401-022-02454-z
3. Bellenguez C, Küçükali F, Jansen IE, et al. New insights into the genetic etiology of Alzheimer's disease and related dementias. *Nature Genetics 2022 54:4*. 2022-04-04;54(4)doi:10.1038/s41588-022-01024-z
4. M A, DB A, P G, et al. Large multi-ethnic genetic analyses of amyloid imaging identify new genes for Alzheimer disease - PubMed. *Acta neuropathologica communications*. 04/26/2023;11(1)doi:10.1186/s40478-023-01563-4
5. Andreassen OA, Thompson WK, Schork AJ, et al. Improved detection of common variants associated with schizophrenia and bipolar disorder using pleiotropy-informed conditional false discovery rate. *PLoS Genet*. Apr 2013;9(4):e1003455. doi:10.1371/journal.pgen.1003455
6. Nordengen K, Cappelletti C, Bahrami S, et al. Pleiotropy with sex-specific traits reveals genetic aspects of sex differences in Parkinson's disease. *Brain*. Mar 1 2024;147(3):858-870. doi:10.1093/brain/awad297
7. C C, JS K, O H, et al. GWAS of cerebrospinal fluid tau levels identifies risk variants for Alzheimer's disease - PubMed. *Neuron*. 04/24/2013;78(2)doi:10.1016/j.neuron.2013.02.026
8. Ries M, Loiola R, Shah UN, Gentleman SM, Solito E, Sastre M. The anti-inflammatory Annexin A1 induces the clearance and degradation of the amyloid-beta peptide. *J Neuroinflammation*. Sep 2 2016;13(1):234. doi:10.1186/s12974-016-0692-6
9. Gauthier-Kemper A, Suarez Alonso M, Sundermann F, et al. Annexins A2 and A6 interact with the extreme N terminus of tau and thereby contribute to tau's axonal localization. *J Biol Chem*. May 25 2018;293(21):8065-8076. doi:10.1074/jbc.RA117.000490
10. Kito Y, Gonza Lez-Robles TJ, Kaisari S, et al. GMCL1 Controls 53BP1 Stability and Modulates Paclitaxel Sensitivity in Cancer. *bioRxiv*. Mar 19 2025;doi:10.1101/2025.03.18.643855
11. Jiang J, Yu W, Fang T, et al. Cyclin O promotes lung cancer progression and cetuximab resistance via cell cycle regulation and CDK13 interaction. *J Thorac Dis*. Apr 28 2023;15(4):2167-2183. doi:10.21037/jtd-23-437
12. Turley P, Walters RK, Maghzian O, et al. Multi-trait analysis of genome-wide association summary statistics using MTAG. *Nat Genet*. Feb 2018;50(2):229-237. doi:10.1038/s41588-017-0009-4
13. Euesden J, Ali M, Robins C, et al. Patient stratification by genetic risk in Alzheimer's disease is only effective in the presence of phenotypic heterogeneity. *PLOS ONE*. Jan 9, 2025;20(1)doi:10.1371/journal.pone.0310977
14. Lu Q, Li B, Ou D, et al. A Powerful Approach to Estimating Annotation-Stratified Genetic Covariance via GWAS Summary Statistics. *The American Journal of Human Genetics*. 2017/12/07;101(6)doi:10.1016/j.ajhg.2017.11.001

15. Hemani G, Zheng J, Elsworth B, et al. The MR-Base platform supports systematic causal inference across the human phenome. *Elife*. May 30 2018;7doi:10.7554/eLife.34408
16. Korvatska O, Leverenz JB, Jayadev S, et al. R47H Variant of TREM2 Associated With Alzheimer Disease in a Large Late-Onset Family: Clinical, Genetic, and Neuropathological Study. *JAMA Neurol*. Aug 2015;72(8):920-7. doi:10.1001/jamaneurol.2015.0979

Reviewer comments responses

Thank you to the reviewers for the additional comments in bold below. We have provided additional clarifications requested by the reviewers.

1. **The GitHub that has the code for this should be provided for FAIR reasons. Not just links to software. This is a pretty comment requirement now.**

Reply: Thank you to the reviewer for pointing out this requirement. The summary statistics file generated during the study are now available at 1) NeuroGenomics and Informatics Center online repository (https://neurogenomics.wustl.edu/open-science/raw-data/), 2) the NIH-approved repository: NIAGADS accession number NG00191, and 3) the GWAS catalog (accession number: Pending).

We have now provided details of codes/scripts used in this project in this Github repository (https://github.com/NeuroGenomicsAndInformatics/GWAS-project). The repository is also citable through Zenodo (https://doi.org/10.5281/zenodo.17780074).

The link to github has also been added to code availability statement in the main manuscript.

We have added the following statement to Data availability and code availability section in the main manuscript:

Page 33, line 5: The GWAS meta-analysis summary statistics generated in this study have been deposited in the NIAGADS database under accession code NG00191 and GWAS catalog (accession code TBD). The files are also available to download from Washington University in St. Louis NeuroGenomics and Informatics Center web portal (https://neurogenomics.wustl.edu/open-science/raw-data/).

Page 33, line 29: Detailed code used in this project is available on github (https://github.com/NeuroGenomicsAndInformatics/GWAS-project) and on Zenodo (https://zenodo.org/records/17780074).

2. The stratified data in Supp table 5 doesn't make sense to me, maybe I need another coffee. P-values seem inconsistent with the meta if you just look at CO versus AD. For example, rs6733839 in this table looks impossible. Was this on the full set of results. p = 8.95E-01 meta-analyzed with p = 3.08E-02 should not generally give you a meta p of 5.24E-09 if that includes all the samples in the two subsets. This is confusing and should be clarified.

Reply: Thank you to the reviewer for the question. The case control study only includes samples that had disease status information available. More specifically, of the 18,948 samples included in meta-analysis, 2,365 AD cases and 2,763 CO were available for the diagnosis stratified analysis. Given this large difference in number of samples in AD/CO analysis and main meta-analysis, we see the difference in statistical significance between these analyses.

We have added the following points in main text to clarify this difference in sample size :

Page 26, line 20: Similarly, diagnosis stratified analyses were performed in AD and CO group using samples that had both raw data and disease status available. In total, 2,365 AD cases and 2,763 CO were available for the diagnosis stratified analysis.
